# Endogenous auditory and motor brain rhythms predict individual speech tracking

**DOI:** 10.1371/journal.pbio.3003924

**Published:** 2026-07-31

**Authors:** Christina Lubinus, Anne Keitel, Jonas Obleser, David Poeppel, Johanna M. Rimmele

**Affiliations:** 1 Department of Cognitive Neuropsychology, Max-Planck-Institute for Empirical Aesthetics, Frankfurt am Main, Germany; 2 Cooperative Brain Imaging Center, Goethe University Frankfurt, Frankfurt am Main, Germany; 3 Psychology, University of Dundee, Dundee, United Kingdom; 4 Department of Psychology, University of Luebeck, Lübeck, Germany; 5 Center for Brain, Behavior, and Metabolism, University of Luebeck, Lübeck, Germany; 6 Department of Psychology, New York University, New York, New York, United States of America; Max Planck Institute for Psycholinguistics, NETHERLANDS, KINGDOM OF THE

## Abstract

Slow, endogenous brain rhythms in the auditory cortex are hypothesized to track acoustic amplitude modulations during speech comprehension. Temporal predictions from the motor system are thought to enhance this tracking. However, direct evidence for the involvement of endogenous auditory and motor brain rhythms is lacking. Combining magnetoencephalographic recordings with behavioral data, we here show that endogenous peak frequencies of individuals’ resting-state theta rhythm in superior temporal gyrus predict speech tracking during comprehension. Importantly, endogenous rates of speech motor areas predicted auditory-cortical speech tracking only in individuals with high auditory–motor synchronization profiles. Higher rates in the supplementary motor area and lower rates in inferior frontal gyrus predicted stronger tracking. These findings provide support for oscillatory accounts of auditory–motor interactions during speech perception. Behaviorally, higher auditory–motor synchronization was related to higher comprehension, with effects of the spontaneous speech motor production rate only in high synchronizers. Working memory capacity predicted speech comprehension performance only in individuals with low auditory–motor synchronization profiles. No significant relationship between the neural data and behavioral readouts was observed. The findings highlight differential speech processing preferences across individuals, with an auditory–motor route related to enhanced comprehension performance.

## Abstract 2

Endogenous brain rhythms are thought to support speech comprehension via auditory and motor interactions, but direct evidence for their role remains limited. This study shows that theta frequencies in superior temporal gyrus predict speech tracking, with motor rhythms enhancing tracking only in individuals with high auditory–motor synchronization.

## Introduction

Verbal communication, by requiring the matching of acoustic and articulatory representations, exemplifies action-perception interactions in humans essential for everyday behavior [1]. In particular, speech production and perception are intricately interwoven. The perception of speech may rely on the identification of intended vocal tract gestures [2], allowing for the prediction of the speech signal, as considered crucial in vocal learning [3]. Others assume a more moderate role of the motor system, such that it is aiding speech perception particularly in demanding listening situations [4,5]. During speech production, the motor system engages in operations requiring precise timing [6,7], with these motor areas possibly being similarly recruited during speech perception [6,8,9]. Specifically, the supplementary motor area (SMA) and (parts of the) inferior frontal gyrus (IFG) are assumed to generate temporal predictions about upcoming sensory events [10–15]. The SMA and IFG take different roles during speech perception, with the SMA receiving input from subcortical loops serving mostly inhibitory input and the IFG more closely linked to auditory cortex processing in the temporal lobe [9,16–18]. Based on the literature, on the one hand, and on our goal to focus on higher-order speech processing and sequencing [19–21], on the other, we operationalized speech motor areas as comprising the pars triangularis of the IFG as well as SMA, while not including for these analyses the region of precentral gyrus implicated in speech motor control [21,22].

A prominent neural oscillatory account of speech perception proposes that slow endogenous brain rhythms in auditory cortex allow for speech segmentation by aligning their neural excitability phase to the speech acoustics (speech tracking; [23–28]). Additionally, neural oscillations from cortical motor areas may be involved in speech perception to various degrees. Slow and fast neural oscillations are argued to aid temporal predictions from motor areas through coupling with auditory areas [10,11,29,30]. The mechanisms of auditory–motor interactions during speech (and auditory) perception, however, are not fully understood, and the involvement of neural oscillations is controversially discussed [31–33]. A crucial characteristic of neural oscillations is that they reflect endogenous brain rhythms observed in the absence of external stimulation. Here, we put a neural oscillatory framework of auditory–motor interactions to a rigorous test by investigating whether individuals’ peak frequencies of endogenous rhythms of auditory and motor cortical areas (observed during resting-state) and their coupling strength predict auditory cortical tracking during speech comprehension.

Oscillatory speech perception models propose that endogenous theta rhythms in auditory cortex synchronize to temporal fluctuations in the speech signal (i.e., the amplitude envelope) and thereby segment it into syllable-sized chunks [24–27,34,35]. This brain-to-speech alignment is most pronounced in the theta range (~5 Hz), declining at higher syllabic rates [36], as speech comprehension also decreases (for non-speech see: [37–41]). Given the observation of endogenous theta brain rhythms in auditory cortex [42–44] and the optimal speech processing in this range, the preferred frequencies of neuronal populations in auditory cortex in the theta range have been proposed to constrain the temporal granularity of perception [26,34,44–48]. The hypothesized connection between endogenous theta rhythms and speech processing, a fundamental aspect of oscillatory theories, however, has been rarely investigated directly, i.e., by relating endogenous and functional processing within individuals [49,50]. Such research may be hindered by the difficulty of quantifying individual endogenous brain rhythms in auditory cortex in the theta range [46]. While it is relatively straightforward to identify individual peak frequencies in some cases (e.g., posterior alpha peak frequency) because those can be detected in average power spectra, this is typically more challenging in most other frequency bands and areas. We here use spectral-fingerprinting of resting-state brain activity [46] to comprehensively identify individual auditory spectral peaks in a completely data-driven way. Specifically, we use time-resolved clustering procedures of normalized data that result in several frequency peaks per individual, which reflect endogenous rhythmic activity.

According to recent work, not only auditory processing but also auditory–motor coupling has an optimal range (~4.5 Hz [29,51]), hinting at the involvement of motor cortex oscillations in the auditory–motor interaction. Computational modeling supports an oscillatory account of auditory–motor coupling during speech perception, assuming oscillators with slightly higher preferred frequencies in the auditory than the motor system [3,29]. Behavioral and computational studies have shown a relation between preferred (spontaneous) motor production rates and the ability to synchronize to sound at different rates in music and sound sequences (for a neural approach see: [52–55]). In the context of dynamical systems theory, the *Arnold tongue* phenomenon describes how an oscillator’s ability to track a stimulus depends on its preferred frequency and the frequency and intensity of an external stimulation [56]. However, how this phenomenon transfers to the preferred frequencies of two oscillatory systems (auditory and motor) and their coupling strength to shape our ability to track speech acoustics is unknown. In a recent behavioral study [41], we approached this question, demonstrating superior speech comprehension in individuals with higher auditory–motor synchronization and higher preferred motor rates. The spontaneous speech synchronization (SSS) test was used to behaviorally estimate auditory–motor cortex coupling strength [57]. The preferred endogenous motor rates were quantified by the spontaneous rhythmic speech production rates of an individual, like the spontaneous tapping measure typically used in the field of dynamic attending [53,58–62].

Here, we use a behavioral and MEG approach in a relatively large sample, combining spectral fingerprinting of individuals’ MEG resting-state brain activity with MEG data recorded during a speech intelligibility task (see Fig 3A). As a speech intelligibility task, we used a *sentence repetition task* to estimate individual’s speech comprehension performance. Additional behavioral tasks (Fig 3A, 3C, and 3E; and the digit-span to access working memory capacity) were conducted. First, we aim to test the neural oscillatory approach of speech segmentation by quantifying predictive effects of individual endogenous theta frequencies in auditory cortex on speech tracking and comprehension. Second, to characterize the conjectured neural oscillatory approach of auditory–motor interactions, we expect predictive effects of individual theta frequencies in motor cortices and of auditory–motor coupling strength. Our behavioral findings replicate and extend our previous behavioral study [41], by showing that higher preferred auditory rates and stronger auditory–motor synchronization predict speech comprehension. In contrast, higher spontaneous speech motor production rates only predicted comprehension in parts of the population (high audio-motor synchronizers), with low synchronizers instead showing predictive effects of their working memory capacity. No detectable relationship between the neural data and behavioral readouts was observed. Importantly, the neural results show that only in individuals with high auditory–motor synchronization profiles, the endogenous frequencies of cortical speech motor areas in SMA and IFG predicted speech tracking in auditory cortices, STG and HG, respectively. In contrast, endogenous auditory frequencies of STG (but not HG) were predictive of speech tracking across the population. Our study provides evidence for a neural oscillatory account that highlights alternative speech processing routes across individuals.

**Fig 1:**
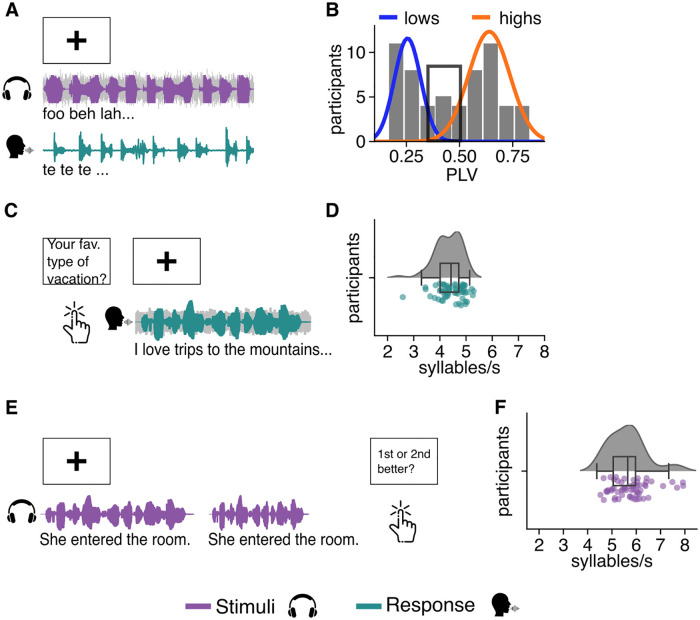
Experimental paradigms and behavioral data. **A.** We measured auditory–motor synchronization using the (explicit) SSS-test [63], wherein participants whisper the syllable/te/ (green speech signal) while listening to a syllable train (purple speech signal) embedded in noise (grey signal). They are instructed to synchronize their motor output to the auditory input. **B.** The histogram illustrates audio-motor synchronization (measured as PLV). Colored lines represent fitted normal distribution, obtained by a Gaussian mixture model. The black box indicates participants that were excluded from the group analysis, as no unique group affiliation could be determined (i.e., probability of 0.5). **C.** To quantify the motor rate, we conducted a speech motor production task. Upon being prompted with a question/statement, participants spoke freely (green speech signal) for 30 **s.** To control for auditory feedback, noise was presented while participants spoke (grey signal). **E.** In the preferred auditory rate preference task, participants indicated which speech rates they liked better. To this end, two versions of a sentence, differing in their syllabic rates, were presented on each trial and participants chose the sentence (i.e., syllabic rate) they preferred. **D, F.** Density and dot plots visualize the motor production rate (**D**) and preferred auditory rate (**F**) of participants. All icons, the “Headphones” by Dong Gyu Yang, “speaking” by Ainul Muttaqin and the “Click” by Wahyu Prihantoro, are taken from The Noun Project used under the Creative Commons Attribution 3.0 license https://creativecommons.org/licenses/by/3.0/. The underlying numerical data shown in B, D, F are available at https://doi.org/10.17605/OSF.IO/SNDPE.

**Fig 2:**
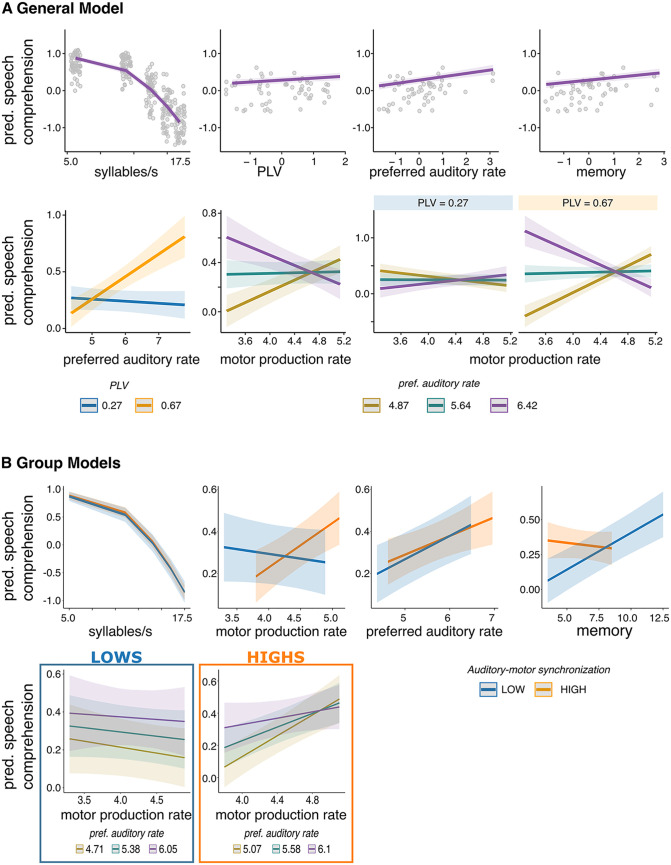
Main and interaction effects predicting speech comprehension in all participants (A) and in high vs. low synchronizers (B). **A.** The general GLMM revealed a negative main effect of syllabic rate (Hz; row 1, column 1) and positive main effects of PLV (row 1, column 2), the preferred auditory rate (Hz; row 1, column 3), and memory (row 1, column 4). The raw data for each participant (dots) were scaled and plotted with the model predictions. For the speech comprehension performance values were averaged across trials for each participant. We further observed interaction effects (right column) between preferred motor rate and PLV (row 2, column 1), preferred auditory rate and motor production rate (row 2, column 2), and a three-way interaction of preferred auditory rate (in Hz), motor production rate (in Hz) and PLV (row 2, column 3–4) **B.** Results from GLMMs computed separately for low (blue) and high (orange,) synchronizers. In low synchronizers, speech comprehension was predicted by the preferred auditory rate and working memory. In contrast, for high synchronizers preferred auditory rate, motor production rate, and their interaction predicted speech comprehension. For optimal comparison of effects between high and low synchronizers, all main effects of the group model are visualized in one panel. The underlying numerical data are available at https://doi.org/10.17605/OSF.IO/SNDPE.

## Results

### Speech comprehension predicted by behavioral auditory and motor parameters

In a behavioral session, participants (*N* = 57) completed behavioral tasks assessing their spontaneous speech motor production rate (henceforth *motor production rate*) (Fig 1C), preferred auditory rate (Fig 1E), and auditory–motor synchronization (Fig 1A). Participants exhibited an average motor production rate of *M* = 4.24 syllables/s (SD = 0.51, Fig 1D). Their preferred auditory rate was about one syllable/s faster than the motor rate with the average at *M* = 5.61 syllables/s (SD = 0.78, Fig 1F). Consistent with previous work [3,41,57], auditory–motor synchronization, measured using the SSS-test, was consistent with a bimodal distribution (Fig 1B). In this sample, *N* = 27 participants were classified as high synchronizers and *N* = 21 as low synchronizers. *N* = 10 participants were excluded from consecutive Group analyses because they were not clearly classified as High/Low synchronizers, while all participants were entered in the General Models.

**Fig 3:**
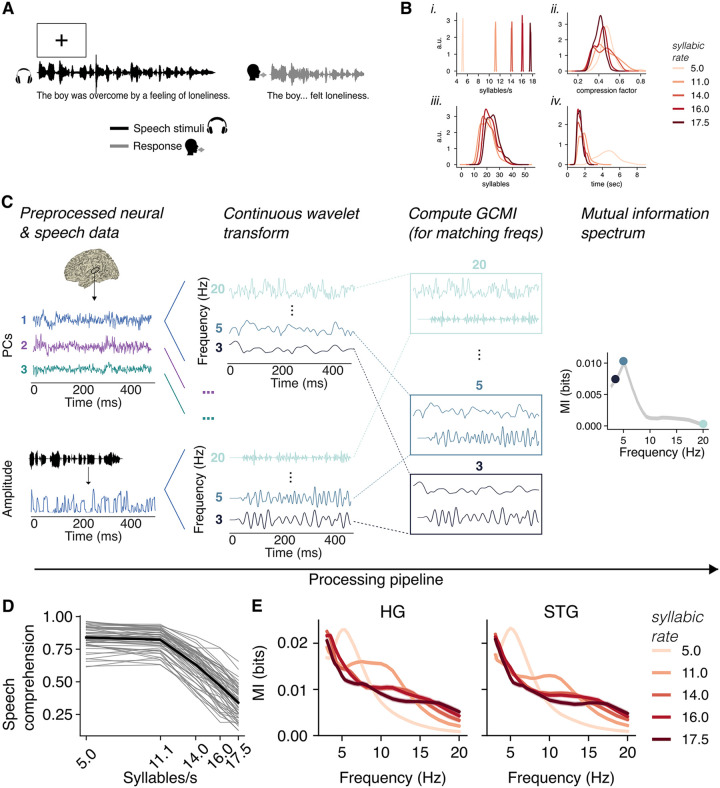
Speech tracking in HG and STG at all syllabic rates. **A.** During the MEG recording session, participants performed the intelligibility task. **B.** Stimulus parameters for sentences presented in the comprehension task. ***i.*** Sentences were presented at five syllabic rates: 5.0, 11.0, 14.0, 16.0, 17.5 syllables/s. ***ii.*** Compression differences were minimized between rate conditions. ***iii.*** Information density across rate conditions was balanced by selecting sentences with overlapping distributions of sentence length (i.e., number of syllables). ***iv.*** However, this came at the cost of not fully equalizing duration. While the four faster conditions were similar in duration, sentences in the slowest condition (5 syllables/s) were notably longer. **C.** Illustration of Gaussian-Copula Mutual Information (GCMI) processing pipeline (reads from left to right). **D.** Line graph displays the behavioral results of intelligibility task: speech was comprehended well at 5 and 11 syllables/s; comprehension deteriorated for the higher syllabic rates. Grey lines illustrate single participant comprehension, thick black line represents the mean over participants. **E.** Non-normalized GCMI spectra in HG and STG. For both panels, lines are color-coded according to the syllabic rate of sentence stimuli (see legend). HG, Heschl’s gyrus; STG, superior temporal gyrus; The brain plot was generated using the Fieldtrip toolbox and plotting the template brain (ROI_MNI_V4.nii using: ft_readatlas) that had been interpolated to a brain surface mesh template (surface_pial_both.mat; using ft_sourceinterpolate) and plotted (using: ft_sourceplot), the ROI has been drawn. All other icons, the “Headphones” by Dong Gyu Yang, “speaking” by Ainul Muttaqin, are taken from The Noun Project used under the Creative Commons Attribution 3.0 license https://creativecommons.org/licenses/by/3.0/. The underlying numerical data (i.e., in B, D, E) are available at https://doi.org/10.17605/OSF.IO/SNDPE.

Speech comprehension was estimated using an intelligibility (sentence repetition) task during the MEG recording session (Fig 3A and 3C). Trial-based comprehension accuracy (% words correct) was regressed against several variables (see Fig 2A and 2B and S3 Table) (*N* = 55). FDR-corrected *p*-values are reported, if not otherwise indicated. As expected, we observed a main effect of syllabic rate, with decreased comprehension for higher syllabic rates (linear: *b* = −77.72, SE = 1.91, *p*_FDR_ < .001; quadratic: *b* = −26.66, SE = 1.01, *p*_FDR_ < .001). Replicating previous findings [41], stronger auditory–motor synchronization (*b* = 0.05, SE = 0.01, *p*_FDR_ = .001) predicted better speech comprehension. In contrast to our previous findings, the motor production rate effect did not reach significance (*b* = 0.02, SE = 0.02, *p*_FDR_ = .266). In addition, faster preferred auditory rates predicted better speech comprehension (*b* = 0.01, SE = 0.02, *p*_FDR_ < .001). These findings were refined by two-way interaction effects of auditory–motor synchronization (PLV) × preferred auditory rate (*b* = 0.08, SE = 0.01, *p*_FDR_ < .001), auditory–motor synchronization (PLV) × motor production rate (*b* = 0.05, SE = 0.02, *p*_FDR_ = .007) and motor production rate × preferred auditory rate (*b* = −0.06, SE = 0.02, *p*_FDR_ < .001), and a three-way interaction of motor production rate × preferred auditory rate × PLV (*b* = −0.08, SE = 0.02, *p*_FDR_ < .001). A positive effect of the motor production rate was particularly observed in high synchronizers (auditory–motor synchronization (PLV) × motor production rate). The positive effect of the preferred auditory rate was stronger in individuals with high motor production rates. The three-way interaction suggests that the interplay of auditory and motor rates is particularly evident in individuals with stronger auditory–motor synchronization behavior. Several control variables facilitated speech comprehension: better working memory performance (*b* = 0.07, SE = 0.01, *p*_FDR_ < .001), shorter sentences (*b* = −0.12, SE = 0.02, *p*_FDR_ < .001), and the time on task (i.e., suggesting performance improvement throughout the experiment, *b* = 0.05, SE = 0.00, *p*_FDR_ < .001).

As it has been hypothesized that high and low synchronizers behave fundamentally differently [3] and to reduce model complexity, we computed separate models for the two groups (Fig 2B and S4 Table). Both groups showed a main effect of syllabic rate (LOW: (linear: *b* = −45.67, SE = 2.02, *p*_FDR_ < .001; quadratic: *b* = −15.82, SE = 0.92, *p*_FDR_ < .001); HIGH: (linear: *b* = −53.61, SE = 1.69, *p*_FDR_ < .001; quadratic: *b* = −19.73, SE = 0.98, *p*_FDR_ < .001). For low synchronizers, we further observed an effect of working memory (*b* = 0.12, SE = 0.02, *p*_FDR_ < .001, see S6 Fig for raw data of working memory). In high synchronizers, speech perception was additionally predicted by a main effect of the preferred auditory rate (*b* = 0.03, SE = 0.01, *p*_FDR_ = .010) and by the motor production rate (*b* = 0.03, SE = 0.01, *p*_FDR_ < .001), and a motor production rate × preferred auditory rate interaction effect (*b* = −0.02, SE = 0.01, *p*_FDR_ = .013). Thereby, individuals with high motor production rates showed high performance independent of the preferred auditory rate. In contrast, in individuals with low motor production rates, higher preferred auditory rates were beneficial. While the statistical power was lower in the model of low synchronizers due to fewer participants (*N* = 21 versus *N* = 27), these results suggest—jointly with the three-way interaction of the main model—that the motor production rate effects and the interaction of the auditory and motor rates are mainly present in individuals with high auditory–motor synchronization.

### Auditory cortex tracking and auditory–motor coupling across syllabic rates

Next, we analyzed whether speech tracking could be predicted by auditory–motor coupling and the endogenous theta frequencies of auditory (HG, STG) and speech motor (IFG pars triangularis, SMA) brain areas. To quantify speech tracking (see Fig 3C for analysis pipeline), we computed Gaussian-Copula Mutual Information (GCMI [64]) between neuronal activity in auditory brain areas (HG and STG) and the speech signal’s amplitude envelope. To simplify the complexity of the statistical model, GCMI values were averaged across hemispheres. To test for significant tracking, GCMI was normalized using surrogate data, yielding z-transformed GCMI values (see S1 Fig). Speech tracking peaked at the syllabic rate of sentences relative to the other tested frequencies (Fig 3E).

Next, we quantified auditory–motor coupling by computing GCMI between speech motor areas (IFG pars triangularis and SMA) and auditory areas (HG and STG; see S2A Fig). Using the optimal delay per condition, we extracted GCMI spectra for all conditions and ROI pairs. GCMI values were averaged across hemispheres. GCMI was normalized using surrogate data, yielding z-transformed GCMI values. However, the normalized GCMI spectrum showed an offset (i.e., a large difference in GCMI values between the syllabic rate conditions that was not present in the non-normalized data) in the 5 syllables/s condition relative to the faster conditions (see S2B Fig). Because the normalization changed the shape of the GCMI spectrum drastically, we treat the normalized data cautiously and conduct subsequent analyses on the non-normalized GCMI values for both speech tracking and auditory–motor coupling.

### Individual rate of endogenous theta brain rhythms in auditory and motor cortex

**Fig 4:**
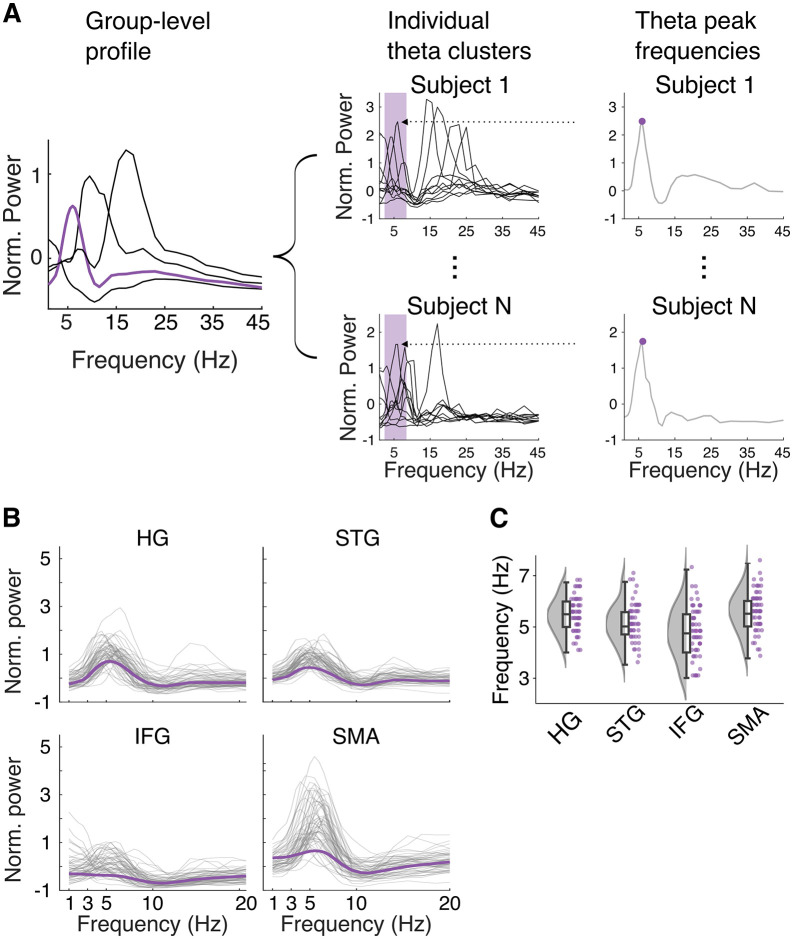
Spectral fingerprints of HG, STG, IFG, and SMA. **A.** Illustration of single-subject theta cluster extraction. Group-level spectral profiles contain multiple spectral clusters, each represented by a line, and express region-specific spectral power relative to the power across the whole brain. For further analysis only the cluster peaking in the theta range (i.e., magenta line) was considered. We reconstructed which single-subject clusters (multiple clusters possible) contributed to the group-level theta cluster and selected the individual theta cluster with the highest amplitude. Finally, the frequency at the cluster peak was extracted as individual theta frequency. **B.** Line graphs illustrate individual theta clusters (grey lines) corresponding to the group-level clusters (thick magenta line), averaged across hemispheres. Note that for IFG the group cluster is also colored in magenta (not grey as done above in panel E) to better distinguish group and individual clusters. **C.** Distribution- and boxplots visualize the peak frequencies extracted from the individual theta clusters. HG, Heschl’s gyrus; STG, superior temporal gyrus; IFG, interior frontal gyrus; SMA, supplementary motor area. The underlying numerical data shown in B and C are available at https://doi.org/10.17605/OSF.IO/SNDPE.

**Fig 5:**
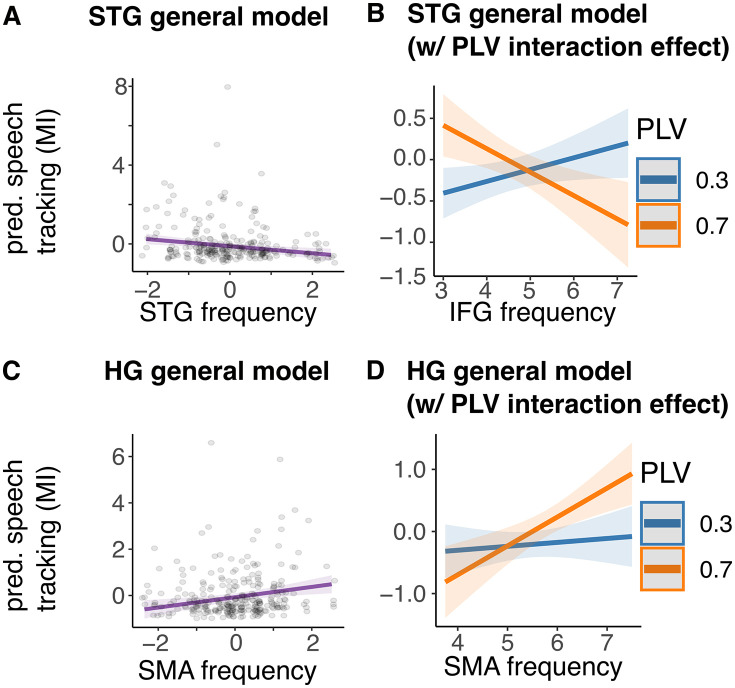
Speech tracking in STG and HG is predicted by distinct auditory and motor variables. **A.** In the STG general model, we observed a negative main effect of the endogenous frequency of STG on speech tracking (in STG). **B.** Including two-way interactions between the behavioral measure of auditory–motor synchronization and motor brain rhythms revealed a significant interaction effect of PLV × IFG, but not PLV × SMA, on speech tracking in STG. **C.** The HG general model revealed a main effect of SMA frequency on speech tracking in HG. **D.** In the HG model with PLV interaction terms, we observed an interaction effect of PLV × SMA, but not of PLV × IFG, on speech tracking. In all panels, error shades indicate 95% confidence intervals. HG, Heschl’s gyrus; STG, superior temporal gyrus; IFG, interior frontal gyrus; SMA, supplementary motor area. The raw data for each participant (dots) were scaled and plotted with the model predictions. All brain-area frequencies (x axes) are in Hz. The underlying numerical data are available at https://doi.org/10.17605/OSF.IO/SNDPE.

We hypothesized that the individual frequency of the endogenous theta rhythms in auditory and speech motor areas (‘eigenfrequency’, as assessed during resting-state) predicts speech tracking. To test this hypothesis, we identified spectral fingerprints in HG, STG, IFG, and SMA using a clustering approach (see S3 Fig). From single-subject clusters, we extracted each participant’s theta frequency for these brain regions and averaged these values across hemispheres (Fig 4A and 4C). While not all subjects exhibited a theta cluster in every ROI, the majority of subjects did (Fig 5B; HG-L: *N* = 55; HG-R: *N* = 56, STG-L: *N* = 56, STG-R: *N* = 55, IFG-L: *N* = 56, IFG-R: *N* = 56, SMA-L: *N* = 57, SMA-R: *N* = 56).

### Auditory–motor parameters affect speech tracking in HG and STG differentially

We combined all neural variables to assess whether speech tracking was affected by the endogenous theta frequency of auditory (HG, STG) and speech motor (IFG, SMA) brain areas, as well as auditory–motor coupling. To this end, we computed two GLMMs, a “HG general model” and an “STG general model”. FDR-corrected *p*-values are reported, if not otherwise indicated.

The STG general model (*N* = 50; Fig 5A, S8 Table) revealed a negative main effect of syllabic rate (linear: *b* = −8.72, SE = 0.83, *p*_FDR_ < .001), reflecting a decrease in speech tracking in STG with increasing syllabic rate. Importantly, the model also showed a main effect of the endogenous theta frequency of STG (*b* = −0.18, SE = 0.06, *p*_FDR_ = .011), suggesting increased speech tracking for individuals with lower endogenous auditory theta frequencies.

The HG general model (*N* = 54, Fig 5C, S5 Table) also revealed a negative main effect of syllabic rate (linear: *b* = −9.27, SE = 0.80, *p*_FDR_ < .001). For the endogenous theta frequency of SMA, we observed a positive main effect (*b* = 0.22, SE = 0.07, *p*_FDR_ = .023), such that speech tracking was higher in individuals with higher endogenous theta frequencies in SMA.

### Motor parameters predict speech tracking in high synchronizers

Based on theoretical assumptions and the behavioral findings, we included two-way interaction effects of auditory–motor synchronization (PLV) and both speech motor rates (IFG, SMA) in the HG and STG general models (see Fig 5B and 5D; S6 and S9 Tables). In the STG model (*N* = 50), we observed a significant interaction effect of auditory–motor synchronization (PLV) and IFG (*b* = −1.01, SE = 0.28, *p*_FDR_ = .003), but not PLV and SMA (*b* = 0.32, SE = 0.43, SE = 0.27, *p*_FDR_ = .118). In contrast, the HG model (*N* = 54) revealed a trend for an interaction effect of PLV and SMA (*b* = 0.77, SE = 0.29, *p* = .009, *p*_FDR_ = .089), but not PLV and IFG (*b* = −0.62, SE = 0.33, *p* = .059, p_FDR_ = .224). As both interaction effects suggest that speech tracking is influenced by the motor rate more strongly in individuals with higher auditory–motor synchronization, we also computed separate GLMMs for high and low synchronizers (see S7 and S10 Tables).

**Fig 6:**
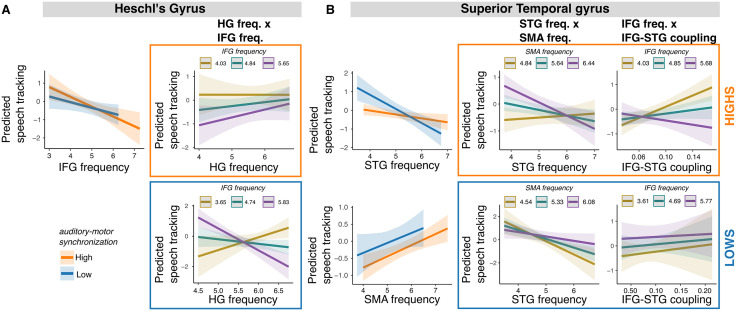
Speech tracking in HG and STG is predicted by different variables in low (blue) and high (orange) synchronizers. **A.** In the HG model, high synchronizers showed a negative main effect of endogenous IFG rate, whereas low synchronizers showed no significant effects after correction for multiple comparisons. **B.** In the STG model, low synchronizers exhibited a negative main effect of STG frequency, while high synchronizers showed a positive main effect of endogenous SMA frequencies. All main effects in A and B are displayed for both groups, even when significant effects were observed in only one group. **C.** In high synchronizers, the STG model additionally revealed two interaction effects involving speech motor areas that were not significant in low synchronizers: STG × SMA rates, and STG–IFG coupling × IFG rate. In all panels, error shades indicate 95% confidence intervals. STG, superior temporal gyrus; IFG, interior frontal gyrus; SMA, supplementary motor area. The underlying numerical data are available at https://doi.org/10.17605/OSF.IO/SNDPE.

In low synchronizers, the HG model (*N* = 20) revealed an interaction effect of the endogenous theta frequencies of IFG and HG (*b* = −0.69, SE = 0.18, *p*_FDR_ = .002, Fig 6B) with speech tracking being highest when IFG and HG theta frequencies were mismatching (i.e., high IFG and low HG frequency, Fig 6A). In the low synchronizers STG model (*N* = 19), we observed a negative main effect of endogenous theta frequency in STG (*b* = −0.49, SE = 0.14, *p*_FDR_ = .008; Fig 6A).

In high synchronizers, the HG model (*N* = 28, Fig 6B) showed a negative main effect for endogenous theta frequency in IFG (*b* = −0.44, SE = 0.14, *p*_FDR_ = .025). Other effects were observed; however, they did not survive correction for multiple comparisons (S7 Table). The high synchronizers’ STG model (*N* = 27, Fig 6A) failed to converge. Therefore, we re-computed the model using a simplified random effect structure (random intercept for subject, S10 Table). The simplified model revealed a positive main effect of SMA theta frequency (*b* = 0.26, SE = 0.09, *p*_FDR_ = .015) in high synchronizers. Furthermore, a significant interaction effect between IFG frequency × STG-IFG coupling (*b* = −0.24, SE = 0.08, *p*_FDR_ = .015) indicated that IFG frequency predicted speech tracking more strongly in individuals with high STG-IFG coupling. Specifically, lower IFG frequencies were related to stronger tracking. Additionally, we observed an interaction effect of the endogenous theta frequencies in SMA and STG (*b* = −0.23, SE = 0.09, *p*_FDR_ = .046). For individuals with low endogenous theta frequency in SMA, speech tracking was unaffected by the theta frequency in STG. However, for individuals with higher theta frequency in SMA, speech tracking varied with theta frequency in STG such that tracking was highest in individuals with lower STG theta frequencies.

In summary, the comparison between HG and STG models reveals that the endogenous theta frequency of STG had a direct association with speech tracking, while the endogenous theta frequency of HG only played a role by interacting with other variables (such as endogenous motor frequencies and coupling). The comparison of low and high synchronizers suggests that high synchronizers exhibit stronger rhythmic speech motor system engagement during tracking (i.e., effects of endogenous motor frequencies and STG-IFG coupling).

### Effects of auditory–motor parameters show no hemispheric asymmetry

Additional analyses were computed to investigate hemispheric asymmetry in speech tracking and its interaction with the auditory–motor parameters. The hemispheric asymmetry index [65] indicates stronger right hemispheric speech tracking, with strongest effects at slower syllabic rates. Asymmetry was significantly greater than zero at most frequencies in both STG and HG (all FDR-corrected *p* ≤ 0.03, strongest at 5 Hz, S15 Table, S4 Fig), whereas no significant differences were observed between high and low synchronizers at any rate (Wilcoxon rank-sum tests; all FDR-corrected *p* > 0.48). Furthermore, using LMMs, the STG general model (*N* = 50, S11 Table) and the HG general model (*N* = 54, S12 Table) were computed with the additional predictors hemisphere and the interactions of hemisphere with endogenous auditory frequency, endogenous motor frequency and auditory–motor coupling. In both the STG and the HG model fixed effects of hemisphere were observed (STG: hemisphere [*r*], *b* = 0.39, SE = 0.08, statistic = 4.64, *p*_FDR_ < 0.001, S11 Table; HG: hemisphere [*r*], *b* = 0.41, SE = 0.1, statistic = 4.19, *p*_FDR_ < 0.001, S12 Table), indicating *stronger speech tracking in the right hemisphere* (S5 Fig). None of the interactions survived the multiple comparison control (all *p*_FDR_ > 0.138). Additionally, to assess whether effects of auditory–motor parameters varied across high and low synchronizers, the STG and HG general model with the auditory–motor synchronization (PLV) interaction effects was computed with the additional predictor of the interaction of hemisphere and auditory–motor synchronization (PLV). No significant interaction with the hemisphere was observed (S13 and S14 Tables). Here besides the fixed effects of the hemisphere (STG model: hemisphere [*r*] *b* = 0.4, SE = 0.08, statistic = 4.67, *p*_FDR_ < 0.001, S13 Table; HG model: hemisphere [*r*] *b* = 0.42, SE = 0.1, statistic = 4.34, *p*_FDR_ < 0.001, S14 Table), no interaction effects of hemisphere and auditory–motor synchronization were observed (STG: PLV × hemisphere [*r*]: *b* = 0.06, SE = 0.08, statistic = 0.75, *p*_FDR_ = 0.452; HG: PLV × hemisphere [*r*]: *b* = 0.05, SE = 0.09, statistic = 0.61, *p*_FDR_ = 0.815).

### Relationship of neural auditory–motor parameters and comprehension

For both behavioral and neural analyses, the effects exhibited similar patterns (Figs 2B and 6), with effects related to endogenous motor brain rhythms observed predominantly in individuals displaying stronger behavioral auditory–motor synchronization (high synchronizers). To directly test the relationship between the neural estimates and speech comprehension, LMM analyses were conducted separately for STG (*N* = 48) and HG (*N* = 52), with performance in the speech intelligibility task predicted by the syllabic rate, the neural tracking in auditory brain areas (HG, STG), the frequencies of auditory (HG, STG) and speech motor areas (IFG, SMA), as well as the auditory–motor coupling. Additionally, we performed LMM analyses with the additional predictor auditory–motor synchronization (PLV) and its interaction with the other parameters. The only significant effects that were observed were effects of the syllabic rate, with higher syllabic rates related to lower comprehension (HG: linear: *b* = −13.68, SE = 0.5, statistic = −27.18, *p*_FDR_ < 0.001; quadratic: *b* = −6.58., SE = 0.32, statistic = −20.81, *p*_FDR_ < 0.001; STG: linear: *b* = −12.8, SE = 0.52, statistic = −24.48, *p*_FDR_ < 0.001; quadratic: *b* = −6.26, SE = 0.32, statistic = −19.79, *p*_FDR_ < 0.001; S16 and S18 Tables; for models with PLV included similar effects were observed, S17 and S19 Tables).

Additionally, to further explore the relationship between the neural estimates and comprehension performance, the analyses were inspected as exploratory analyses without FDR correction. For the HG model findings revealed a positive effect of SMA coupling (*b* = 0.05, SE = 0.02, statistic = 2.05, *p* = 0.042) and a negative effect of HG speech tracking, with higher coupling and lower tracking related to higher comprehension (*b* = −0.1, SE = 0.05, statistic = −2.11, *p* = 0.036, S16 Table; similar results were observed for the model with auditory–motor synchronization (PLV) predictors included, S17 Table). For the STG model, a positive effect of SMA coupling was observed (*b* = 0.06, SE = 0.02, statistic = 0.1, FDR = 0.027, S18 Table). We found no interactions with auditory–motor synchronization (PLV, S19 Table). Note that given that no FDR correction was applied, these findings are not considered significant, however, future research may further investigate the nature of the relationship.

Finally, we investigated whether behavioral and neural estimates of the auditory and motor preferred rates were correlated). We hypothesized that behavioral measures of the auditory and motor rates reflect behavioral readouts of the underlying—supposedly—oscillatory properties of the corresponding brain systems. However, correlation analyses showed no significant relations between the corresponding neural and behavioral rates (all *p*s > .05, S20 Table).

## Discussion

**Fig 7:**
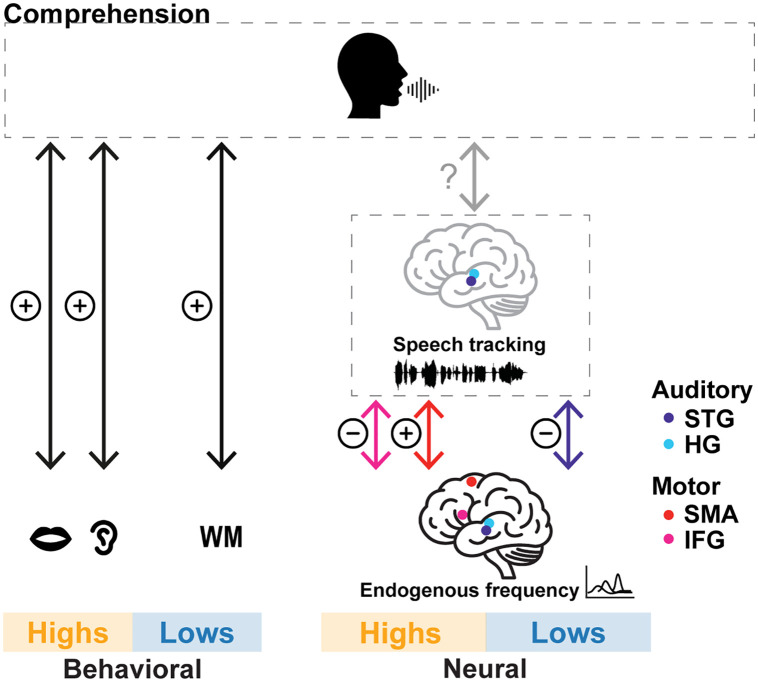
Schematic summary of the results. The left part of the figure illustrates the effects of the behavioral auditory–motor parameters on speech comprehension (black speaker icon). Individuals with high auditory–motor synchronization (Highs) show effects of the spontaneous speech production rate (arrow, mouth icon) on speech comprehension and effects of the preferred auditory rate (arrow, ear icon). In contrast, low auditory–motor synchronizers (Lows) show effects of working memory (arrow, WM icon). The direction of the effects is indicated (±). The right part of the figure illustrates the effects of the neural auditory–motor parameters on speech comprehension. In Highs, speech tracking in HG was predicted by the frequency of the endogenous speech-motor rhythm in IFG (pink arrow, pink region of interest, ROI), with lower rates related to higher tracking. Speech tracking in STG was predicted by the frequency of the endogenous speech-motor rhythm in SMA (red arrow/ROI), with higher frequencies related to higher tracking. In the Lows, higher tracking in STG was predicted by lower frequencies of the endogenous auditory brain rhythm in STG (blue arrow/ROI). The behavioral results similar as the neural results show effects of the spontaneous production rate and the endogenous motor brain rhythms only in high auditory–motor synchronizers. In an exploratory analysis (with no control for multiple comparisons), higher speech comprehension was predicted by lower tracking in HG (transparent arrow), the findings; however, require further research. The “mouth” icon by little_dipper_studio, the “Ear” icon by Gregor Cresnar, the “Brain” icon by Lewen Design and the “speaking” icon by Ainul Muttaqin are taken from The Noun Project used under the Creative Commons Attribution 3.0 license https://creativecommons.org/licenses/by/3.0/.

Preferred frequencies of an individual’s endogenous (resting-state) auditory and motor brain rhythms predict their speech tracking in cortical auditory areas during continuous listening (Fig 7). This is our main result, and supports a framework of endogenous oscillations shaping perception. It significantly refines models of auditory speech tracking and auditory–motor interactions, revealing that distinct processes may be recruited to different degrees for different parts of the population. Specifically, we find that across the population, speech tracking in higher-order auditory cortex (STG) was predicted by individual frequencies of endogenous theta brain rhythms in this area. Furthermore, individual frequencies of endogenous theta brain rhythms of cortical speech motor areas (particularly SMA, and IFG through interactions with STG–IFG coupling) predicted speech tracking in auditory association areas - but only in individuals with behaviorally quantified high auditory–motor synchronization.

A slightly different picture emerges for primary auditory cortex (which we here operationalize as Heschl’s gyrus, HG). No main effect of the frequency of the endogenous HG rhythm was observed, neither across the population nor separately in low or high synchronizers. In high synchronizers, as obtained for auditory association cortex, endogenous frequencies of theta brain rhythms of cortical speech motor areas (particularly IFG) predicted speech tracking in HG. Primary auditory cortex thus seems more receptive to external stimulation rates and less rhythmically constrained by its endogenous theta brain rhythm than STG. We found no evidence for a significant correlation between neural estimates and comprehension performance. An exploratory analysis, however, indicated the relationship as a potential topic for future research. Together the data suggest that during speech comprehension, individuals with higher auditory–motor synchronization use motor top–down predictions through coupled auditory–motor oscillators. In contrast, low auditory–motor synchronizers rely more on auditory oscillatory populations and possibly may more heavily recruit working memory processes, as should be further explored by future research.

### Speech comprehension and tracking decline at higher syllabic rates

As expected, we found that comprehension decreased at higher syllabic rates (sharply above 11 syllables/s). Although this decline was observed at slightly higher rates than previously reported in studies using simpler stimuli (e.g., words or short sentences [24,36,66]), recent studies have observed similar effects [41,67,68]. The shallower decline in comprehension performance across rates for more complex speech stimuli may be explained by enhanced comprehension due to linguistic predictability, particularly at faster syllabic rates [41]. Note that auditory cortex showed amplitude envelope tracking even at non-intelligible rates. Previous studies [36] reported that comprehension was predicted by neural tracking. Others, however, did not report a correlation, pointing towards a more complex link between comprehension and tracking [67,69–71]. Although comprehension and neural tracking both declined at higher rates, we find no significant correlation between them. Thus overall, the relationship between neural tracking and comprehension seems to be complex, likely due to both measures being affected by various factors differently [72].

### The frequency of endogenous brain rhythms in STG predicts speech tracking

Endogenous theta rhythms in cortical auditory areas are believed to phase-align to the acoustic envelope of incoming speech [23,26,27,43,73]. Our study, to our knowledge for the first time, establishes a direct relationship between an individual’s rate of the endogenous theta rhythm (measured during rest) and speech tracking in auditory association cortex (STG) during comprehension. We found that lower endogenous theta frequencies in STG were consistently associated with stronger speech tracking. This was shown in both high and low synchronizers in the general model (see also: S7 Fig; S22–S26 Tables). However, effects were stronger in low synchronizers, as seen in the group models. This finding directly suggests recruitment of an oscillatory population during speech tracking [26,44,46,49]. It is possible that for individuals who more strongly rely on the auditory system for speech processing and recruit the motor system less, a lower endogenous theta frequency is advantageous because it is close to the natural syllabic rate of speech (~5 Hz). For individuals who strongly recruit the motor system instead, because cortical auditory and motor areas more strongly interact, whether lower or higher endogenous theta frequencies are advantageous may depend on the frequency of the endogenous theta motor rhythm (as suggested by the interaction effect of auditory and motor frequency). In contrast, the endogenous theta frequency of primary auditory cortex (HG) was not predictive of speech tracking. We speculate that due to a stronger preference for speech signals in STG [42,74], the role of its endogenous rhythm may be more relevant to speech tracking. Intracranial results have demonstrated that parts of STG preferentially represent “syllable-level temporal structure“ [74], whereas HG reflects less complex acoustic features [74,75]. Furthermore, oscillatory neuronal populations in STG may be involved in speech segmentation [26,42], with possibly weaker oscillatory properties in HG.

### Endogenous motor-cortex rhythms predict speech tracking in high synchronizers

Endogenous theta rhythms of two cortical speech motor regions reliably predicted speech tracking in high, but not in low auditory–motor synchronizers. (Note that we defined SMA and IFG as speech motor areas in this study.) The endogenous theta frequencies of SMA and IFG exhibited contrasting effects on speech tracking: higher SMA rates were associated with higher tracking, higher IFG rates with lower tracking. This is consistent across tracking in auditory association and primary auditory cortex (STG and HG; Figs 5 and 6). Interestingly, STG particularly showed effects of the frequencies of SMA (less so for IFG), while the opposite was observed for HG. Given SMA’s role in temporal processing [9,76–78] and IFG’s involvement in temporal sequencing and speech motor processing [9,79], these findings indicate that high synchronizers may rely more heavily on temporal motor predictions for speech processing, compared to low synchronizers.

This interpretation is further supported by our behavioral observation of increased comprehension performance in high synchronizers. The endogenous theta frequency in IFG showed a negative effect, indicating enhanced tracking with lower endogenous frequencies. Given the proposed direct connection between IFG and STG [9], observing the same direction of effects may imply a synergistic effect of (the endogenous frequencies in) these two areas on tracking. In contrast, the positive effect of the endogenous theta frequency in SMA on tracking paralleled our behavioral findings and showed the expected direction of the effect. The discrepancies between the SMA and IFG effects may reflect differences in network connections, as well as whether the areas are connected in an excitatory or inhibitory manner [80]. Further research is necessary to elucidate the distinct relation of endogenous IFG and SMA rhythms to speech tracking.

The finding of selective motor effects for high synchronizers is in line with previous work [3,39,41,57,81,82]. Particularly, Assaneo, Rimmele and colleagues [3] proposed, based on behavioral data and a neural computational model, that oscillatory auditory–motor coupling was engaged preferentially in high synchronizers. Here, in low synchronizers, we only detected an interaction effect between the endogenous theta peak frequencies in IFG and HG. High compared to low synchronizers may not only rely more strongly on the recruitment of the speech motor system but also more extensively engage additional areas like (parts of) the SMA, reflecting other aspects of temporal processing or alternative processing routes [9]. Behaviorally, particularly speech comprehension in low but not high synchronizers was predicted by working memory capacity. According to predictive coding frameworks, if an incoming signal can be predicted accurately by an internal predictive model, less sensory processing is needed and cognitive resources are released [83,84]. More specifically, during speech comprehension predictions from cortical motor areas about how the speech signal sounds, may reduce the reliance on phonological storage [85]. This may be an explanation for this finding, leading to the speculation of different speech processing routes, with high synchronizers relying more on predictions from the motor system, related to enhanced comprehension performance, and low synchronizers relying more on auditory processing and an auditory working-memory contribution.

### Auditory–motor coupling interacts to affect speech tracking

Overall, we observed similar patterns for theta phase auditory–motor coupling between different motor (IFG, SMA) and auditory (HG, STG) areas (S2 Fig), with significant auditory–motor coupling at all syllabic rates. Coupling, however, decreased from 5 Hz to 11 Hz and slightly increased again at faster rates. The findings align with reports of a “sweet-spot” for auditory–motor coupling around 4.5 Hz [29]. Furthermore, such a sensitivity for certain frequencies in the theta- and beta-range has been previously shown for the auditory cortex [86] and for the motor cortices (as reflected in their endogenous rhythms [46,48]). Speech tracking was predicted by coupling only in high synchronizers, who showed an interaction effect of IFG-STG coupling and the IFG frequency on STG tracking. Specifically, effects of the endogenous IFG theta frequency on speech tracking were more pronounced in individuals with high auditory–motor coupling strength. Our results are limited by the focus on phase–phase theta coupling. Further research is required to understand oscillatory processing routes within a spectrally and spatially more complex auditory–motor network.

### No hemispheric asymmetry of auditory–motor parameters

In line with previous research [87], speech tracking in auditory cortical areas (STG, HG) was stronger in the right compared to the left hemisphere. Like speech tracking, auditory–motor interactions can show hemispheric lateralization, whereas the strength of lateralization may depend on various influences [88]. However, we found no lateralization of the effects of the peak frequencies of the endogenous auditory and motor brain rhythms and the auditory–motor coupling on speech tracking. Future research may provide further insights into these complex interactions.

### Relationship of neurophysiological and behavioral findings

Replicating our two behavioral experiment(s) [41], we show that higher speech comprehension was related to higher auditory–motor synchronization and preferred auditory rates (in our previous study, the latter was a trend that did not survive multiple-comparison control). A three-way interaction between these variables and the individual spontaneous motor production rates suggests a complex interplay of the behaviorally assessed preferred rates and auditory–motor synchronization. Interestingly, the behavioral and neural findings show similar patterns of results for high and low synchronizers. In low synchronizers, comprehension was predicted by working memory scores. In contrast, in high synchronizers, comprehension was predicted by the preferred auditory rate, and, crucially, the motor production rate and the interaction of auditory and motor rates.

We found no direct relationship between the auditory–motor neural estimates and speech comprehension. Although, an exploratory analysis hints at a possible relationship. Lower speech tracking in Heschl’s Gyrus may be related to higher performance in the intelligibility task, and higher auditory–motor coupling between SMA and Heschl’s Gyrus and SMA and STG to higher comprehension. These findings were not significant; however, may inspire future research. Speech comprehension is intricate and shaped by numerous interacting variables. While the neuronal dynamics underlying speech tracking are crucial, they likely represent just one aspect of the number of computations—including linguistic and situational predictions, as well as working memory—that facilitate speech comprehension.

### Conclusions

We demonstrate that *endogenous* theta rhythms of STG predict speech tracking. Interestingly, endogenous theta rhythms of speech motor areas (SMA, IFG) were predictive of speech tracking only in those individuals showing high behavioral auditory–motor synchronization. Our findings are consistent with an oscillatory model of auditory–motor interactions during speech comprehension. While some individuals recruit the speech motor system (SMA, IFG), likely providing temporal predictions to enhance comprehension, others seem to predominantly rely on auditory processing. Furthermore, our findings suggest differences between primary auditory and association areas in their oscillatory characteristics relevant for speech tracking and in their processing routes.

## Materials and methods

### Ethics statement

The study was approved by the local ethics committee of the University Hospital of the Goethe-University Frankfurt (Ethikkomission des Fachbereichs Medizin der Goethe-Universität, approval number: 2021-509) in accordance with the Declaration of Helsinki. Prior to each session, participants provided written informed consent.

Our magnetoencephalography (MEG) and behavioral experiment entailed three separate sessions: a behavioral session, an MEG session with behavior, and a structural magnetic resonance imaging (MRI) session.

### Participants

Overall, the data of *N* = 57 participants were analyzed (age: *M* = 26.9, SD = 5.4; 32 female, self-report of gender, i.e., German “Geschlecht”). The initial sample consisted of *N* = 60 participants (*N* = 3 participants were excluded because of technical issues during recording, and *N* = 2 were excluded from behavioral analyses because of an average performance of 3 standard deviations below average in the baseline condition (5 syllables/s) of the speech comprehension task). As assessed by self-report, participants had no history of neurological or psychiatric diseases and had normal hearing, as well as normal (or corrected-to-normal) vision. All participants were native speakers of German and right-handed. At the end of the final session, participants received monetary compensation. Due to analysis-specific exclusion criteria, different numbers of subjects were included in the different analyses. Specifically, participant numbers were affected by (1) the SSS test and (2) the extraction of the individual theta peak frequency at auditory and audio-motor brain areas. The number of participants included in each analysis and the exclusion criteria are stated in the respective analysis methods and results section—if they diverge from the general sample of 57 subjects.

### Stimuli

In two experimental tasks (speech comprehension task and preferred rate task) participants listened to naturalistic sentences. The sentences were sourced from German books (*N*_sentences_ = 306; 8 talkers; source: zeno.org) and audiobooks (*N*_sentences_ = 138; 3 talkers; source: Librivox.org), respectively. Sentences from books were recorded by three native talkers of German at the MPIEA in a sound-attenuated booth using MatLab R2017a on a Windows 7 Pro (64-bit) and a Neumann U87i studio microphone, and A/D conversion at 44.1 kHz. Talkers delivered the sentences at their normal, slowest, and maximal speaking rate, while prioritizing proper articulation over speed. All sound files, including audio books, underwent processing using Praat (6.0.40). Long pauses (>300 ms) were removed to prevent inaccurate rate (syllables/second) estimates.

Three stimulus lists were generated from the sentence materials for both the speech comprehension task (300 sentences) and the auditory rate preference task (132 sentences). Time compression or expansion was applied to all sentences to create different syllabic rate conditions. Sentences were randomly selected (without replacement) from the total pool of sentences based on two main criteria: the degree of compression required to obtain the desired syllabic rate (compression factor <3) and the duration of the sentence after compression (>1 s; only applied to comprehension task). No sentence repetitions occurred within each stimulus set across tasks. Time compression or expansion was performed using the Pitch Synchronous Overlap and Add (PSOLA) algorithm in Praat, and all stimuli were standardized to a root mean square (RMS) amplitude at 69 dB (see Fig 3). The selection of syllabic rates (with distributions around five syllabic rates: 5.00, 11.00, 14.00, 16.00, 17.50 syllables/s) was informed by our previous study to capture the full range of variance in comprehension [41].

### Experimental tasks

#### Intelligibility task.

To measure speech comprehension, participants performed an intelligibility task ([89,90]; Fig 4A). The performance in the sentence repetition task has been shown to be highly correlated with “information transfer tasks” that are used to measure comprehension (i.e., content questions regarding a heard sentence) [90]. Therefore, it has been widely used as a proxy for comprehension [91]. On each trial, participants listened to a sentence via headphones and verbally repeated it as accurately as possible. All responses were recorded, and participants stopped the recording via button press (left or right index finger), initiating the interstimulus interval. Sentences were presented at five syllabic rates (5.00, 11.00, 14.00, 16.00, 17.50 syllables/s) with 60 different sentences each, totaling 300 trials. Trials with different syllabic rates were pseudorandomized within blocks of 30 trials, with self-paced breaks between blocks.

#### Spontaneous speech synchronization (SSS) test.

To assess auditory–motor synchronization, participants performed the SSS test (Fig 1A; for a detailed description, see the General Introduction, study 2 and Assaneo and colleagues [57]. In two trials, participants continuously whispered a syllable (/te) for 80 seconds and aimed to synchronize their own motor output to a stream of syllables. Their whispering was recorded. The auditory stimulus progressively increased in rate from 4.3 to 4.7 syllables/s (increments of 0.1 syllables/s) every 60 syllables. Participants’ syllable production was masked by the simultaneously presented auditory syllable train.

#### Speech production task.

The individual spontaneous speech motor production rate was measured by asking participants to freely speak “as they would naturally” (Fig 1B). Participants were prompted with 6 questions/statements to facilitate continuous speech production (6 trials; own life, preferences, people, culture/traditions, society/politics, general knowledge; see S2 Table [92]. Participants read the question/statement and initiated the speaking period (30 s) via button press. White noise was presented via headphones to minimize auditory feedback. Breaks in between trials were self-paced.

#### Auditory rate preference task.

The preferred auditory rate was assessed using a two-interval forced choice (2IFC) task (Fig 1C). On each trial, we presented two versions of the same sentence, randomly ordered, differing only in syllabic rate. After stimulus presentation, participants indicated which of the two stimuli they preferred via button press. Stimuli were presented at 12 syllabic rates from 3.0 to 8.5 syllables/s (in steps of 0.5 syllables/s). Each syllabic rate was paired against every other rate, resulting in 132 unique trials. Trials were presented in blocks of 30 trials, with self-paced breaks in between. Crucially, the trial sequence was randomized, without grouping by syllabic rate.

#### Digit span test.

Working memory capacity was quantified using the forward and backward [93] digit span test. Participants listened to digit spans and typed in their responses after listening [94]. The test comprised seven levels, ranging from two/three to nine digits with two items at each level. The procedure started with the shortest digit spans (forward: three digits, backward: two digits) and stopped as soon as participants failed to repeat both spans of the same length or when the two longest digit spans were reached.

#### Procedure: Behavioral session.

Participants were seated in a sound-attenuated experimental booth equipped with a Fujitsu CELSIUS M740B PC. Stimuli were presented using Psychtoolbox (Brainard, 1997) in Matlab (9.7.0.1471314, R2019b) and insert earplugs (ER3C Tubal Insert Earphones; Etymotic Research). Participants’ speech and whisper was recorded using a gooseneck microphone (MX418 microflex gooseneck microphone, Shure) and behavioral responses were collected using a standard keyboard. Stimuli were presented at ~70 dB in the preferred auditory rate and digit span tasks. For the spontaneous speech production task and the SSS-test, loudness could be adjusted using a volume control knob on the sound card. Participants were instructed to increase the volume until their own speech or whisper became inaudible, while still being comfortable. The rationale behind this procedure is to isolate motor production by suppressing auditory feedback.

All participants started with the spontaneous speech motor production rate task to avoid priming effects. Next, participants performed either the SSS-test or the preferred auditory rate preference task, the order was randomized across participants. Finally, all participants finished with the digit span task, followed by the questionnaire (demographics and musicality).

#### Procedure: MEG session.

After application of EOG and ECG channels, participants were seated in the MEG. The stimuli were delivered binaurally using insert earplugs (EARTONE Gold 3A insert earphones; Ulrich Keller Medizin-Technik) and the Matlab (R2017a) software with the Psychtoolbox (3.0.14; Brainard, 1997) extension on a Fujitsu-Technology CELSIUS R940power PC. Participants’ responses were recorded using a button box (Current Designs Package 932). Fiducials were attached to participants’ nasion and the preauricular points to continuously measure head position.

First, participants performed the intelligibility task. The task was split into 10 blocks (~5 min each). Second, participants completed an auditory localizer task in which they passively listened to a sequence of sounds (pure tones: 0.4 s tone duration; 250 Hz and 1,000 Hz, 100 repetitions, jittered intertrial interval 0.5–1.5 s; ~5 min). Third, participants performed a motor localizer task (~12 min) during which they repeatedly articulated syllables (i.e., performed the movement) without vocalizing. On each trial, one out of three syllables (/pa/,/ta/,/sa/) was articulated for 3 s and each syllable was repeated in 50 trials. Note that the motor localizer data was not analyzed in this study. Finally, we recorded resting state activity (~5 min 30 s). Throughout the experiment, participants were instructed to hold their gaze at a fixation cross (except during instructions). In total, the MEG session had a duration of roughly 180 min, consisting of 120 min recording time and 60 min of preparation time and scanning pauses.

MEG data were acquired at a sampling rate of 1,200 Hz using a 275-channel whole-head MEG machine (Omega 2005, CTF Systems) in a magnetically shielded room. During scanning, online denoising (higher-order gradiometer balancing) and online low-pass filtering (cut-off: 300 Hz) were applied. Furthermore, participants’ head position was measured continuously using fiducials and the Fieldtrip toolbox ([95], version 20220617), allowing for position adjustment between blocks as well as continuous head movement correction at the analysis stage.

#### Procedure: MRI session.

Individual structural MRI scans (standard 1 mm T1-weighted MPRAGE) were obtained using a 3 Tesla scanner (2 participants were scanned on a Siemens Magnetom Trio scanner; all other participants were scanned on a Siemens Magnetom Prisma, scanner Siemens, Erlangen, Germany. Vitamin E capsules were used to mark anatomical landmarks (nasion, left and right pre-auricular points) to align MRI and MEG data for source reconstruction.

### Behavioral analysis

#### Speech comprehension.

Participants’ responses were transcribed manually. To quantify comprehension accuracy, we compared the responses to the original sentences using a sequence matcher algorithm (Python built-in sequence matcher), which quantifies the similarity between two sequences by (order of) item, i.e., letter. The output of the sequence matcher was a percentage for each sentence.

#### Preferred auditory rate.

From the 2IFC task, we derived the preferred frequencies for each trial. This distribution of preferred frequencies across trials was fitted using a Gaussian function. From the fitted Gaussian, the peak parameter was extracted and defined as individual preferred auditory frequency.

#### Spontaneous speech motor production rate.

The spontaneous speech motor production rate was quantified as articulation rate (i.e., number of produced syllables divided by trial duration, excluding pauses > 300 ms). To segment the continuous speech recordings into syllables, syllable nuclei were detected automatically using Praat [96]. The spontaneous speech motor production rate was computed for each trial separately and then averaged across trials.

#### Auditory–motor synchronization.

The data from the SSS-test were analyzed according to the protocol of Lizcano-Cortés and colleagues [63]. First, data quality was ensured by confirming that participants whispered (i.e., no vocal cord activation) and that audio files were not corrupted (i.e., noise interference). For the analysis, we applied the scripts provided by the authors [63]. Auditory–motor synchronization was measured as the phase-locking value (PLV) between the speech envelope of the produced motor signal and the cochlear envelope of the syllable stimulus. For the syllable train, the cochlear envelope was extracted using the Chimera toolbox (auditory channels: 180–7,246 Hz) [97]. For the produced motor signal, the amplitude envelope was estimated using the Hilbert transform (hilbert.m function in Matlab). Both envelopes were down-sampled to 100 Hz and bandpass filtered between 3.5 and 5.5 Hz. From the filtered signal, we extracted the phase (using hilbert.m and phase.m functions in Matlab), computed the PLV for time windows of 5s (overlap 2 s) and averaged across time windows. This procedure was completed for both experimental runs separately. To test for consistency between runs, a linear regression was fitted to the data (independent variable: PLV of run 1, dependent variable: PLV of run 2). Participants with PLV pairs outside the 95th confidence interval were excluded from further analysis. For the remaining participants, PLVs from both runs were averaged. The mean PLVs were subjected to a Gaussian mixture model to partition participants into high and low synchronizers. While we obtained PLVs for all *N* = 57 participants, 10 could not be classified clearly as high synchronizers (probability being a high synchronizer = 45%–55%). Accordingly, the final sample contained *N* = 27 high synchronizers and *N* = 20 low synchronizers. Note, however, that all participants, regardless of being high or low synchronizers, were included in total sample analysis where the group assignment was secondary.

### MEG analysis: Speech comprehension task

#### Speech envelope extraction.

For the speech signals presented during the comprehension task in the MEG, we extracted the cochlear envelope using the Chimera toolbox (auditory channels: 180–7,246 Hz) [97]. Specifically, the spectrograms were computed for multiple frequency bands (auditory channels: 180–7,246 Hz) and the absolute values then averaged to obtain the broadband speech envelope. Broadband envelopes were down-sampled to 100 Hz to match MEG signals.

#### Preprocessing.

During MEG preprocessing, the data were bandpass filtered (0.5–160 Hz, Butterworth filter; filter order 4) and line-noise was removed (49.5–50.5, 99.5–100.5, 149.5–150.5 Hz, two-pass; filter order 4). We applied a semi-automatic artifact rejection procedure to detect jump, muscle, and threshold artifacts. For jump and muscle artifacts, data were filtered to optimize artifact detection (muscle: 110–140 Hz, jump: median filter) and then z-transformed per sensor and time point. Trials were rejected if they surpassed a priori defined thresholds (jump: *z* = 45, muscle: *z* = 15). For the threshold artifacts, trials were rejected if the range (min − max difference) of activity at any channel surpassed an a priori defined threshold (threshold = 0.75e − 5). The data were down-sampled to 500 Hz and epoched (−1,000 ms to +100 ms after trial offset), resulting in epochs of variable length. Using the continuous fiducial measures, trials containing head movements larger than 4 mm were identified and rejected. Data from the separate recording blocks were concatenated into one large file and sensors with high *z*-values (*z* > 2) were rejected. Finally, eye blink, eye movement and heartbeat artifacts were corrected using independent component analysis (infomax algorithm; [98]). Data were further down-sampled to 100 Hz for computational efficiency.

#### Source localization.

Using the three anatomical landmarks (nasion, left, and right preauricular points), individual T1-weighted MRI images were co-registered to the MEG coordinate system in a semiautomatic procedure. T1-weighted MRIs were segmented into three tissues (white matter, grey matter, cerebrospinal fluid) to construct single-shell volume conduction models (headmodel) [99] and warped into MNI space to compute individual grids by inverse warping the template grid (grid resolution: 5 mm) onto individual anatomical scans. Using the individual grids and volume conduction models, we computed individual forward models to reconstruct source activity.

For source reconstruction, we used a Linearly Constrained Minimum Variance (LCMV) Beamformer (array-unit LCMV) [100]. Firstly, we computed the covariance matrix across all trials and created a common filter for all conditions using the individual forward models. The lambda regularization parameter was set to 1% and time series were extracted for all three dipole orientations per voxel. Secondly, trial data was source-localized by projecting it through (i.e., multiplying it with) the common filter for each condition separately. Computing the common filter across all conditions—instead of separate filters for each condition—ensures that differences between conditions reflect differences in source activity, instead of differences in the spatial filters itself.

#### Regions of interest.

For the speech tracking analysis, regions of interest (ROI, as defined by the Automated Anatomical Labeling (AAL) atlas [101]) included primary and non-primary auditory cortex, comprising Heschl’s gyrus (HG) and superior temporal gyrus (STG), respectively. Both areas are crucial for speech perception, with the STG showing increasing responsiveness to complex acoustic speech features compared to HG, as supported by neurocognitive models and empirical studies [74,102–104]. Notably, speech tracking has been consistently observed in both HG and STG across multiple studies [12,26,105–109].

For auditory–motor coupling, in addition to HG and STG, the ROIs also included two speech motor areas that are well-established in the speech motor network. IFG, a key component of the core language network [103] which might contribute to the auditory–motor coupling during speech perception [11,57], exhibits direct structural connections with the auditory cortex [9,110] and is involved in various speech processing subroutines such as phonological encoding [111] or semantic and sentential information integration [112]. Importantly, IFG synchronizes to speech envelopes [57,106] and shows differing degrees of synchronization depending on individual auditory–motor speech coupling [57]. Interestingly, the auditory–motor coupling of IFG has been proposed to reflect neural oscillatory activity [113]. The SMA, although not traditionally considered a language area, plays a central role in speech motor control [6] and, more recently, has been recognized to contribute to higher-level processes during speech processing [9]. Together with the cerebellum and basal ganglia, SMA processes temporal structure [114] and sensory temporal information more generally [76–78]. Notably, SMA is particularly implicated in adverse listening situations [115], possibly through top-down mechanisms, predicting upcoming sensory events [78,116].

We extracted the corresponding parcels for all ROIs (13 + 14: IFG pars triangularis; 19 + 20: SMA, 79 + 80: HG, 81 + 82: STG) from the source-localized data using the AAL atlas. To further reduce the dimensionality of the data, principal components were computed across voxels within each parcel. To this end, for each trial, all voxel time-series within a parcel were stacked (three time-series, i.e., for each dipole orientation, per voxel) and principal components and their explained variance were extracted. Based on previous work [117], the further analysis only included the first three principal components.

#### Mutual information: Speech tracking.

Speech tracking was estimated using information theory [106,118,119]. Specifically, we computed GCMI [64] to assess statistical dependencies between speech envelopes and source-localized neural activity in auditory and motor ROIs. GCMI capitalizes on the concept of gaussian copulas which is a statistical description of the relationship of two variables regardless of their respective marginal distributions. Mutual information (MI) can be computed as the negative entropy of a statistical copula. This approach ranks the data (to make it marginally uniform), maps them to a Gaussian distribution, and then computes mutual information under the assumption of Gaussian dependencies. This facilitates estimation of MI because (1) no assumptions of the marginal distributions of the variables are needed and (2) the computation is efficient as a Gaussian parametric estimation can be employed. A further advantage of the GCMI approach is that it lends itself naturally to dealing with multivariate data [64]. This allowed us to use phase information of speech envelopes and source-localized activity for multiple frequency bands.

For the estimation of GCMI, we applied a continuous wavelet transform for 28 frequencies (3.5–20 Hz) to the source-localized principal components for each trial using *cwtfilterbank.m* in Matlab. To avoid onset effects, filtered principal components were epoched again from 200 ms relative to stimulus onset until trial end. From the complex signals, we first extracted the phase information (real and imaginary parts) and then copula-normalized the parts separately. Finally, the normalized components were concatenated within each condition. This yielded two frequency-by-samples vectors per condition, i.e., source time series. Importantly, the same procedure (continuous wavelet transform, epoching, extraction of phase information, concatenation) was applied to the speech envelope data with the only difference that each trial only consisted of one time-series per trial (no PCA). Analogous to the source time-series, this resulted in two speech time-series per condition (real and imaginary parts).

In a multivariate analysis, we estimated GCMI between 2 speech time-series and 6 source time-series for each parcel and frequency. To account for stimulus-brain lags, we applied our GCMI estimation at various positive delays (0 ms to 300 ms, 10 ms steps).

#### Mutual information: Auditory–motor coupling.

To examine the hypothesis that audio-motor coupling strength predicts speech tracking (and comprehension), we estimated audio-motor coupling using GCMI. This analysis was identical to the speech tracking analysis above with respect to frequency analysis and GCMI parameters. The important difference lies in the selection of time series: here GCMI was computed between MEG time courses from two different brain areas. Specifically, we computed GCMI between HG or STG (both left and right) and IFG pars triangularis or SMA (both left and right).

### Mutual information: Surrogate data

We tested whether speech tracking and auditory–motor coupling exceeded chance-level within each condition by comparing the observed MI values to the 99th percentile of surrogate data. Surrogate data refers to artificially generated datasets that preserve key statistical properties of the original data (e.g., amplitude distribution or autocorrelation structure) while disrupting the specific dependencies or relationships of interest.

Surrogate data for both analyses (speech tracking and auditory–motor coupling) were generated by segmenting the concatenated trials of each condition, shuffling segments across iterations (*N* = 500), and computing GCMI between one intact and one shuffled timeseries. For speech tracking, GCMI was estimated between each source time series and the shuffled speech time series. For auditory–motor coupling, GCMI was computed specifically between the motor ROI source time series and the shuffled auditory ROI time series. Segment length was condition-specific and set to 80% of the cycle length corresponding to each syllabic rate (5 Hz = 160.0 ms; 11 Hz = 72.72 ms; 14 Hz = 57.1 ms; 16 Hz = 50 ms; 17.5 Hz = 45.7 ms).

We used the surrogate distribution to create z-transformed GCMI values, thus subtracting true GCMI values by the mean surrogate distribution and dividing the result by the surrogate’s standard deviation. The normalization was performed within participants for each parcel, condition, and syllabic rate. GCMI values varied systematically as a function of delay and condition; we extracted GCMI for each condition at the delay that maximized the GCMI values.

### MEG analysis: Resting state

#### Preprocessing.

For the resting state data, preprocessing was similar to the main task but adjusted in a few aspects to match the preprocessing procedure employed in previous studies [48]. Specifically, all parameters for artifact rejection were identical to the main task of the current study. The data were detrended, down-sampled to 250 Hz and epoched into 0.8 s trials, which resulted in an average of *M* = 380 trials (sd = 59.8, min = 121, max = 412). Sensors were rejected based on the neighborhood ratio using the *hcp_qc_neighstddratio.m* function (Human Connectome Project, WU-Minn Consortium), which is defined by the sensors noise level (SD) relative to the noise level of neighboring sensors ([SensorSD − NeighborSD]/NeighborSD). Sensors were rejected if the ratio exceeded a value of 0.5. For trial rejection based on head movements and the independent component analysis, the same procedures as in the main data of the current study were implemented.

#### Common filter for source projection.

We used the same headmodels as in the main analysis. Forward models were obtained using the same procedure as for the main task, but with a different grid (resolution = 10 mm; see study 1 and [46]). For source projection, we used an LCMV Beamformer. First, we computed the covariance matrix across all trials for each individual. Second, the common filter was generated using the previously computed forward models. The lambda regularization parameter was set to 7% and the optimal dipole orientation for each voxel was extracted (single value decomposition). The beamformer coefficients were obtained in preparation for the later source projection of Fourier spectra.

#### Spectral analysis and source projection.

We used Fourier transforms of DPSS taper-weighted time segments (DPSS multitaper, 2 tapers, ± 2 Hz spectral smoothing). DPSS multitaper were applied for spectral smoothing. Complex Fourier spectra of the DPSS taper-weighted segments were computed in sensor space for individual epochs (0.8 s epochs were zero-padded to a duration of 2 s after the DPSS tapers were applied). Zero padding was performed to retrieve frequency bins of the Fourier transform output with 0.5 Hz spacing. Frequencies at 1–120 Hz with a logarithmic frequency spacing were selected for further analyses in the spectral profile clustering analysis.

#### Clustering spectral data.

For each participant and parcel, the source-localized single-trial Fourier spectra were subjected to the k-means algorithm. The k-means algorithm treated each trial’s spectrum as a 42-dimensional space and grouped all trials into *k* mutually exclusive spectra according to spectral (dis)similarities. These spectral clusters represent the spectral modes the brain area engages in over time. We set *k* to 10 (see [46]), used the Cosine distance metric, and initiated the k-means algorithm 10 times (max. 100 iterations). To quantify the proportion of trial-spectra—as determined by the k-means algorithm—belonging to each cluster, *k*-means clusters were subjected to a Gaussian Mixture (GM) Model algorithm.

Prior to the group-level analysis, we determined the optimal number of clusters across participants for each parcel. We evaluated the Silhouette criterion (1,000 iterations) for solutions between 1 and 15 clusters and selected the number of clusters characterized by the highest Silhouette value. To obtain group-level spectra for each parcel, single-subject clusters were stacked and then fed into the k-means algorithm. The results were again subjected to the GM Model algorithm.

#### Selection of individual source-localized theta peaks.

We extracted individual peak frequencies from the theta clusters for HG, STG, SMA, and IFG (see above for motivation of ROIs). Spectral clusters were defined as theta cluster if their peak frequency was within the range of 4–8 Hz. To this end, first, we extracted single-subject spectral clusters that contributed to the theta group-level cluster in question. From the single-subject clusters, we computed the peak frequency (henceforth *endogenous theta frequency of area X*). If multiple clusters from one participant contributed to the group cluster, the cluster with the highest peak amplitude was selected. On the group level, HG was characterized by two theta clusters. We extracted the individual clusters contributing to the lower group cluster because it was contributed to by *N* = 56 participants, compared to *N* = 44 for the higher cluster.

### Statistical analysis

To assess the hypothesized links between speech comprehension and speech tracking and the corresponding predictor variables, we computed Generalized Linear Mixed Models (GLMM). Models were computed using R (version 4.1.3, 2022-03-10) set up in Rstudio (version 2022.2.1.461). For the GLMMs, we used the R package *lme4* (version 1.1-35.1). Tables and effects are visualized using *sjPlot* (version 2.8.15) and *ggplot2* (version 3.4.4). For all models, continuous predictor variables were z-transformed. All results are corrected for multiple comparisons using false discovery rate (FDR) correction, unless stated otherwise. Statistical inference was conducted using the lmerTest package, using the Satterthwaite’s method to approximate the denominator degrees of freedom. Model coefficients (beta), standard errors (std. Error), *t*-values, *p*-values (*p*, FDR-corrected *p*-values, *p*_FDR_), and estimated degrees of freedom (df) are reported in the supplementary tables. As measure of effect size, partial eta-squared (*ηp*^*2*^) values are for fixed effects are approximated using the *eta_squared()* function.

First, for the behavioral GLMM, trial-wise speech comprehension performance (% words correct) was regressed against syllabic rate, preferred auditory rate, spontaneous speech motor production rate, PLV, working memory score, compression factor, sentence length (in syllables), and stimulus order. We were particularly interested in the interaction effect of PLV × preferred auditory rate × spontaneous speech motor production rate. Thus, in addition to main effects, the model contained a three-way interaction. As random effects, we introduced a by-participant random slope for syllabic rate and a random intercept for trial-ID. Overall, the model (*N* = 57) explained 47.8% of the variance. To reduce the complexity, we computed the model separately for high and low synchronizers, which allowed us to reduce the three-way interaction to a two-way interaction (no PLV term). These high (*N* = 27) and low (*N* = 20) synchronizer models explained 47.1% and 48.1% of the variance, respectively.

Second, we designed GLMMs that computed the relationship between speech tracking and the neural predictor variables. All neural variables (speech tracking, endogenous rates, auditory–motor coupling) were averaged across hemispheres. We computed two separate models, one focused on HG (“HG general model”) and one on STG (“STG general model”). Participants were excluded from this analysis if they did not display single-subject spectral peaks in the respective brain areas. In the HG general model (*N* = 54), speech tracking in HG was regressed against syllabic rate, auditory–motor coupling (IFG-to-HG and SMA-to-HG) and the endogenous theta frequencies of HG, IFG, and SMA. The model included two three-way interactions between auditory–motor coupling and the corresponding endogenous theta frequencies. The STG general model (*N* = 50) was identical except that all HG variables were exchanged for STG variables (endogenous rate and coupling). Overall, the HG model explained 24.8% of the variance, the STG model 35.6%. Analog to the behavioral GLMM, additionally, we first added the PLV as predictor to the general models for HG and STG and then computed the models separately for high and low synchronizers. Partitioning the sample into high and low synchronizer groups resulted in the following sub-samples: HG_highs: *N* = 27, HG_lows: *N* = 20, STG_highs: *N* = 27, STG_lows: *N* = 19.

#### Hemispheric asymmetry analysis.

Additional analyses were conducted to assess hemispheric asymmetry in speech tracking and its relationship to auditory–motor synchronization. Hemispheric asymmetry was quantified using the hemispheric asymmetry index (HAI; see [62]), with positive values indicating stronger right-hemispheric tracking. The HAI was computed separately for each syllabic rate and region of interest, including STG and HG. To test whether hemispheric asymmetry differed from zero, one-sample Wilcoxon signed-rank tests were performed at each syllabic rate within each region. Resulting *p*-values were corrected for multiple comparisons using the FDR. To examine whether hemispheric asymmetry differed between high and low synchronizers, Wilcoxon rank-sum tests were conducted at each rate and region, with FDR correction applied across frequencies (full statistical results in S15 Table; visualizations in S4 Fig).

Additionally, GLMMs of our main analyses for speech tracking in HG and STG were computed with the categorical predictor hemisphere (left, right) and auditory–motor synchronization (PLV) included, as well as the interaction between hemisphere and PLV and hemisphere and the frequency of the endogenous auditory and motor brain rhythms (full statistical results in S11–S14 Tables; visualizations in S5 Fig)

## Supporting information

S1 Fig Normalized MI spectra for speech tracking in auditory cortex. Line graph illustrates normalized MI spectra in Heschl’s gyrus (HG, left) and posterior superior temporal gyrus (STG, right). For both panels, lines are color-coded according to the syllabic rate of sentence stimuli and shaded error bars represent standard error of the mean across participants.
(PDF)

S2 Fig Raw MI spectra for auditory–motor coupling. Line plots illustrate raw (**A**) and normalized (**B**) auditory–motor coupling (MI) between different auditory and motor brain areas as a function of frequency: IFG-HG, SMA-HG (top), IFG-STG, SMA-STG (bottom). Lines are color-coded according to the syllabic rate of sentence stimuli and shaded error bars represent standard error of the mean across participants. HG = Heschl’s gyrus, STG = superior temporal gyrus; IFG = interior frontal gyrus; SMA = supplementary motor area.
(PDF)

S3 Fig Group-level spectral fingerprints in the left (top) and right (bottom) hemisphere. Clusters are color-coded according to their peak frequency (legend on the right). Shaded error bars reflect standard error of the mean across participants. HG = Heschl’s gyrus; STG = posterior superior temporal gyrus; IFG = interior frontal gyrus; SMA = supplementary motor area.
(PDF)

S4 Fig Hemispheric asymmetry in speech tracking. Hemispheric asymmetry index (HAI) values are shown for high (orange) and low (blue) synchronizers across syllabic rates in Heschl’s gyrus (HG; top) and posterior superior temporal gyrus (STG; bottom). Positive values indicate stronger right-hemispheric speech tracking. The underlying numerical data are available at https://doi.org/10.17605/OSF.IO/SNDPE.
(PDF)

S5 Fig Stronger speech tracking in the right HG and STG. Additional mixed models were computed, including hemisphere as categorical predictor. In both HG and STG, speech tracking was significantly stronger in the right, than the left hemisphere (see also Supplementary models 11,12). The underlying numerical data are available at https://doi.org/10.17605/OSF.IO/SNDPE.
(PDF)

S6 Fig Distribution of memory scores. Distribution of working memory performance scores for participants classified as high synchronizers and low synchronizers. Scores are plotted separately for each group. Working memory scores do not differ between groups, as assessed using a Wilcoxon Test (*W* = 375.5, *p*-value = 0.188).
(PDF)

S7 Fig Correlations between the theta brain rhythm of various regions of interest and speech tracking in STG and HG and performance in the intelligibility task. We pre-selected all brain areas from the frontal, central and temporal cortex and choose those areas that consistently showed theta cluster across the studies Keitel and Gross (2016) and Lubinus and colleagues (2021). Additionally, two parietal areas were chosen. If for these brain areas a theta cluster was present in a larger number of participants, the area was chosen as region of interest (ROI) for the correlational analysis. The following 15 ROIs from the AAL atlas were analyzed: Frontal_Inf_Tri_, Supp_Motor_Area_, Heschl_, Temporal_Sup_, Parietal_Inf_, Precentral_, Insula_, Rolandic_Oper_, Supra_Marginal_Gyrus_, Frontal_Inf_Oper_, Frontal_Sup_Medial_, Frontal_Mid_, Frontal_Sup_, Frontal_Inf_Orb_, Cingulum_Mid_. Peak frequencies of the theta cluster with maximum amplitude were selected for each participant. Peak frequencies were averaged across the left and right hemisphere. Spearman correlations between the peak frequencies of the endogenous brain rhythms in each ROI and the speech tracking in STG (upper row; averaged across hemispheres and syllabic rate conditions), speech tracking in HG (middle row; averaged across hemispheres and syllabic rate conditions) and performance (bottom row) were computed. False-discovery (FDR) control for multiple comparisons was applied. R-values are color coded. Although, several ROIs show significant effects, only the negative correlations of STG peak frequencies with STG speech tracking survive multiple comparison control. The underlying statistic data, displayed in this figure are available in S26 Table. The brain plots were generated using the Fieldtrip toolbox by mapping the *R*-values onto the respective ROIs of the AAL atlas template brain (ROI_MNI_V4.nii using: ft_readatlas) that had been interpolated to a brain surface mesh template (surface_pial_both.mat; using ft_sourceinterpolate) and plotted (using: ft_sourceplot).
(PDF)

S1 Table Sentence materials: Titles and authors of (audio)books. List of sources from which stimuli for the tasks (speech comprehension and preferred auditory rate tasks) were constructed.
(PDF)

S2 Table Prompts for speech production task. Thematic questions used to facilitate natural speech production. Each item is representative of a different thematic category, as introduced by Alexandrou and colleagues (2016). Sentences were translated to German.
(PDF)

S3 Table Predicting single-trial speech comprehension performance. (PDF)

S4 Table Predicting single-trial speech comprehension performance, separately for high and low synchronizers. (PDF)

S5 Table HG general model: Predicting speech tracking in HG. (PDF)

S6 Table Predicting speech tracking in HG, including behaviorally measured auditory–motor synchronization (PLV) as predictor. (PDF)

S7 Table Group models: Predicting speech tracking in HG, separately for high and low synchronizers. (PDF)

S8 Table STG general model: Predicting speech tracking in STG. (PDF)

S9 Table Predicting speech tracking in STG, including behaviorally measured auditory–motor synchronization (PLV) as predictor. (PDF)

S10 Table Group models: Predicting speech tracking in STG, separately for high and low synchronizers. (PDF)

S11 Table Predicting speech tracking in STG, including a categorical predictor for hemisphere, both as main effect and as interaction term with the endogenous frequencies of STG, SMA, IFG. (PDF)

S12 Table Predicting speech tracking in HG, including a categorical predictor for hemisphere, both as main effect and as interaction term with the endogenous frequencies of HG, SMA, IFG. (PDF)

S13 Table Predicting speech tracking in STG, including a categorical predictor for hemisphere and PLV (behavioral measure of auditory–motor synchronization), both as main effects and as interaction term PLV × hemisphere. (PDF)

S14 Table Predicting speech tracking in HG, including a categorical predictor for hemisphere and PLV (behavioral measure of auditory–motor synchronization), both as main effects and as interaction term PLV × hemisphere. (PDF)

S15 Table Statistical results of hemispheric asymmetry analyses. Results of Wilcoxon rank-sum tests comparing High versus Low synchronizers and Wilcoxon signed-rank tests assessing whether the hemispheric asymmetry index (HAI) differed from zero (Vs. Zero). Results are reported for each syllabic rate and region of interest (HG and STG), including W statistics and FDR-corrected *p*-values.
(PDF)

S16 Table Predicting speech comprehension from neural audio-motor estimates involving Heschl’s Gyrus speech tracking. (PDF)

S17 Table Predicting speech comprehension from neural audio-motor estimates + PLV involving Heschl’s Gyrus speech tracking. (PDF)

S18 Table Predicting speech comprehension from neural audio-motor estimates involving STG. (PDF)

S19 Table Predicting speech comprehension from neural audio-motor estimates + PLV involving STG. (PDF)

S20 Table Spearman rank correlations between spectral peak measures and behavioral frequency variables. Correlations were computed between theta frequency of speech-production–related regions (inferior frontal gyrus, IFG; supplementary motor area, SMA) and the preferred motor rate, and between theta frequency of auditory regions (superior temporal gyrus, STG; Heschl’s gyrus, HG) and the preferred auditory rate. Analyses were conducted both separately for left and right hemispheres and for bilateral averages (mean of left and right homologous regions). Correlation coefficients (Spearman’s *ρ*) and corresponding two-tailed *p*-values are reported. *P*-values were additionally corrected for multiple comparisons using the false discovery rate (FDR) procedure. All correlations were computed using pairwise complete observations.
(PDF)

S21 Table Spearman rank correlations between spectral peak measures in various ROIs and speech tracking in Superior Temporal Gyrus (STG). Peak frequencies of the theta cluster of endogenous brain rhythms with maximum amplitude were selected for each participant. Peak frequencies were averaged across the left and right hemisphere. Spearman correlations between the peak frequencies in each region of interest (ROI, AAL labels are displayed) and the speech tracking in STG (averaged across hemispheres and syllabic rate conditions) were computed. Correlation coefficients (Spearman’s *ρ*, *rho*) and corresponding two-tailed *p*-values are reported. *P*-values are additionally reported corrected for multiple comparisons using the false discovery rate (FDR) procedure (*p*_FDR_). Significance is indicated by asterisks (* < .05; ** < .01).
(PDF)

S22 Table Spearman rank correlations between spectral peak measures in various regions of interest (ROIs) and speech tracking in Heschl’s Gyrus (HG). Peak frequencies of the theta cluster of endogenous brain rhythms with maximum amplitude were selected for each participant. Peak frequencies were averaged across the left and right hemisphere. Spearman correlations between the peak frequencies in each region of interest (ROI, AAL labels are displayed) and the speech tracking in HG (averaged across hemispheres and syllabic rate conditions) were computed. Correlation coefficients (Spearman’s *ρ*, *rho*) and corresponding two-tailed *p*-values are reported. *P*-values are additionally reported corrected for multiple comparisons using the false discovery rate (FDR) procedure (*p*_FDR_). Significance is indicated by asterisks (* < .05; ** < .01).
(PDF)

S23 Table Spearman rank correlations between spectral peak measures in various regions of interest (ROIs) and speech comprehension performance. Peak frequencies of the theta cluster of endogenous brain rhythms with maximum amplitude were selected for each participant. Peak frequencies were averaged across the left and right hemisphere. Spearman correlations between the peak frequencies in each region of interest (ROI, AAL labels are displayed) and the performance in the speech intelligibility task (averaged across syllabic rate conditions) were computed. Correlation coefficients (Spearman’s *ρ*, *rho*) and corresponding two-tailed *p*-values are reported. *P*-values are additionally reported corrected for multiple comparisons using the false discovery rate (FDR) procedure (*p*_FDR_). Significance is indicated by asterisks (* <.05; ** <.01).
(PDF)

S24 Table Spearman rank correlations between spectral peak measures in various ROIs and speech tracking in the Superior Temporal Gyrus (STG) separately computed for the left and right hemisphere. Peak frequencies of the theta cluster of endogenous brain rhythms with maximum amplitude were selected for each participant. Spearman correlations between the peak frequencies of the left and right hemisphere in each region of interest (ROI, AAL labels are displayed) and the left hemispheric speech tracking in STG, as well as the right hemispheric tracking in STG were computed separately. Correlation coefficients (Spearman’s *ρ*, *rho*) and corresponding two-tailed *p*-values are reported. *P*-values are additionally reported corrected for multiple comparisons, within the left and right hemispheric tracking analyses separately, using the false discovery rate (FDR) procedure (*p*_FDR_). Significance is indicated by asterisks (* <.05; ** <.01).
(PDF)

S25 Table Spearman rank correlations between spectral peak measures in various ROIs and speech tracking in the Heschl’s Gyrus (HG) separately computed for the left and right hemisphere. Peak frequencies of the theta cluster of endogenous brain rhythms with maximum amplitude were selected for each participant. Spearman correlations between the peak frequencies of the left and right hemisphere in each region of interest (ROI, AAL labels are displayed) and the left hemispheric speech tracking in HG, as well as the right hemispheric tracking in HG were computed separately. Correlation coefficients (Spearman’s *ρ*, *rho*) and corresponding two-tailed *p*-values are reported. *P*-values are additionally reported corrected for multiple comparisons, within the left and right hemispheric tracking analyses separately, using the false discovery rate (FDR) procedure (*p*_FDR_). Significance is indicated by asterisks (* <.05; ** <.01).
(PDF)

S26 Table Spearman rank correlations between spectral peak measures in various ROIs of the left and right hemisphere and speech comprehension performance. Peak frequencies of the theta cluster of endogenous brain rhythms with maximum amplitude were selected for each participant. Spearman correlations between the peak frequencies of the left and right hemisphere in each region of interest (ROI, AAL labels are displayed) and the performance in the intelligibility task were computed. Correlation coefficients (Spearman’s *ρ*, *rho*) and corresponding two-tailed *p*-values are reported. *P*-values are additionally reported corrected for multiple comparisons using the false discovery rate (FDR) procedure (*p*_FDR_). Significance is indicated by asterisks (* <.05; ** <.01).
(PDF)

## Abbreviations

AAL: Automated Anatomical Labeling
FDR: false discovery rate
GCMI: Gaussian-Copula Mutual Information
GLMM: Generalized Linear Mixed Models
GM: Gaussian Mixture
HAI: hemispheric asymmetry index
HG: Heschl’s gyrus
IFG: inferior frontal gyrus
LCMV: Linearly Constrained Minimum Variance
MEG: magnetoencephalography
MI: mutual information
MRI: magnetic resonance imaging
PLV: phase-locking value
PSOLA: Pitch Synchronous Overlap and Add
RMS: root mean square
ROI: regions of interest
SMA: supplementary motor area
SSS: spontaneous speech synchronization
STG: superior temporal gyrus
2IFC: two-interval forced choice
