# Supplementary material for: Endogenous auditory and motor brain rhythms predict individual speech tracking
Source: PLoS Biol. 2026 Jul 31;24(7):e3003924. doi: 10.1371/journal.pbio.3003924 (PMC13446743; doi:10.1371/journal.pbio.3003924)
Supplement: Supplementary file 1 [file pbio.3003924.s001.pdf]

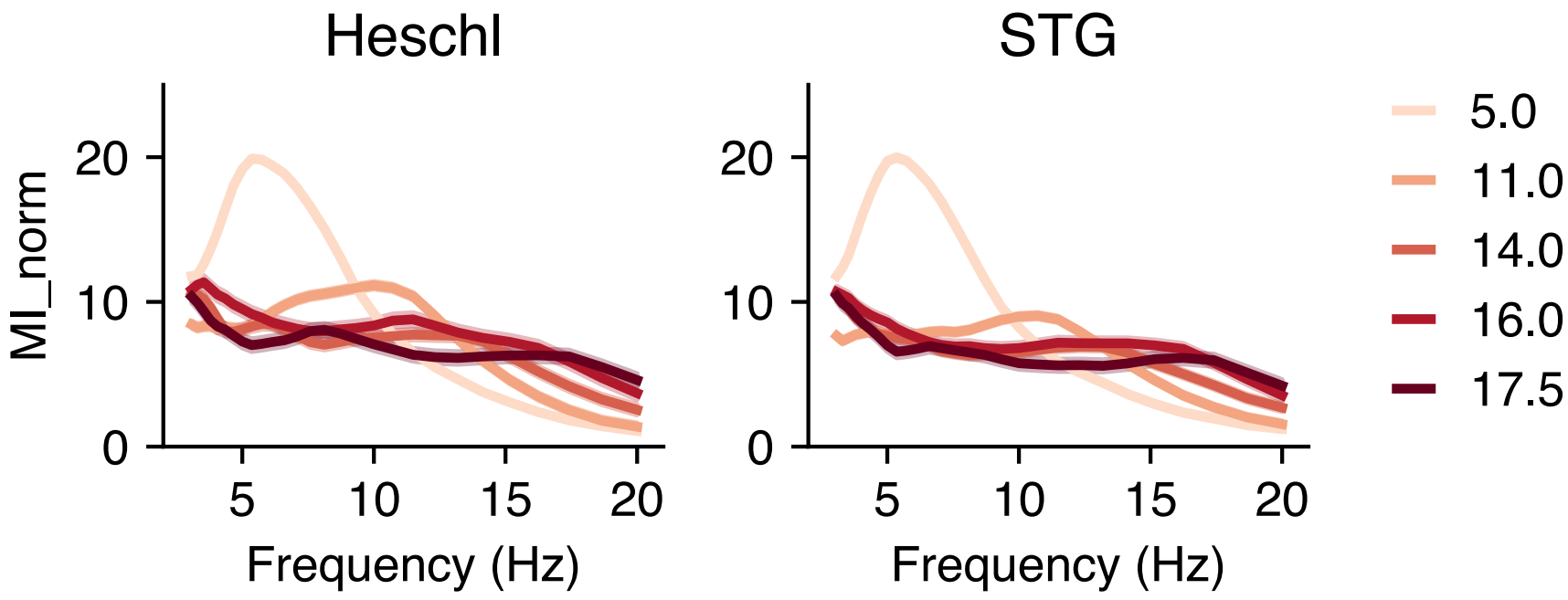

S1 Fig. Normalized MI spectra for speech tracking in auditory cortex. Line graph illustrates normalized gaussian-copula mutual information (MI) spectra in Heschl's gyrus (HG, left) and posterior superior temporal gyrus (STG, right). For both panels, lines are color-coded according to the syllabic rate of sentence stimuli and shaded error bars represent standard error of the mean across participants.
