# Supplementary material for: Endogenous auditory and motor brain rhythms predict individual speech tracking
Source: PLoS Biol. 2026 Jul 31;24(7):e3003924. doi: 10.1371/journal.pbio.3003924 (PMC13446743; doi:10.1371/journal.pbio.3003924)
Supplement: Supplementary file 2 [file pbio.3003924.s002.pdf]

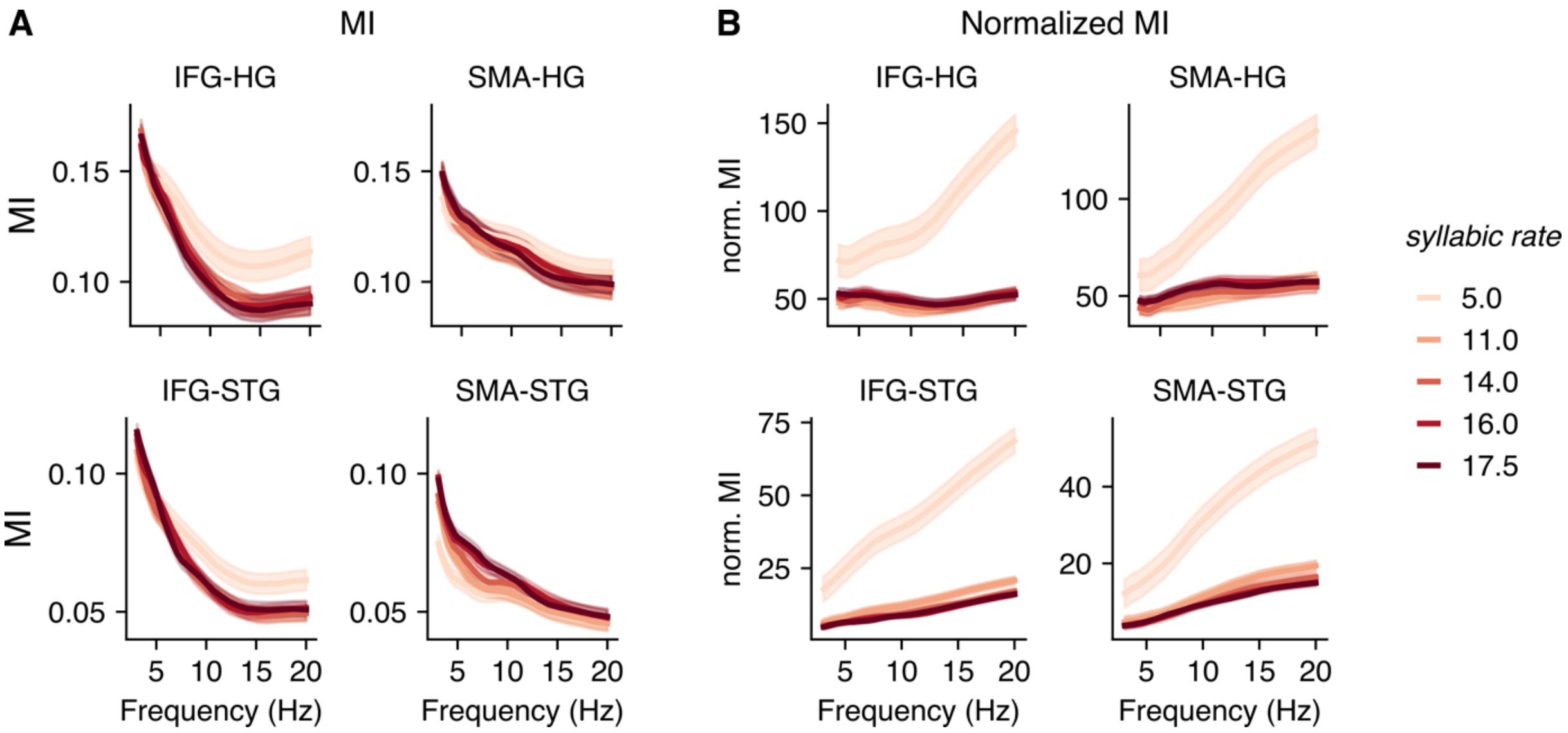

S2 Fig. Raw MI spectra for auditory-motor coupling. Line plots illustrate raw (**A**) and normalized (**B**) auditory-motor coupling (MI) between different auditory and motor brain areas as a function of frequency: IFG-HG, SMA-HG (top), IFG-STG , SMA-STG (bottom). Lines are color-coded according to the syllabic rate of sentence stimuli and shaded error bars represent standard error of the mean across participants. HG = Heschl's gyrus, STG = superior temporal gyrus; IFG = interior frontal gyrus; SMA = supplementary motor area.
