# Supplementary material for: Endogenous auditory and motor brain rhythms predict individual speech tracking
Source: PLoS Biol. 2026 Jul 31;24(7):e3003924. doi: 10.1371/journal.pbio.3003924 (PMC13446743; doi:10.1371/journal.pbio.3003924)
Supplement: Supplementary file 3 [file pbio.3003924.s003.pdf]

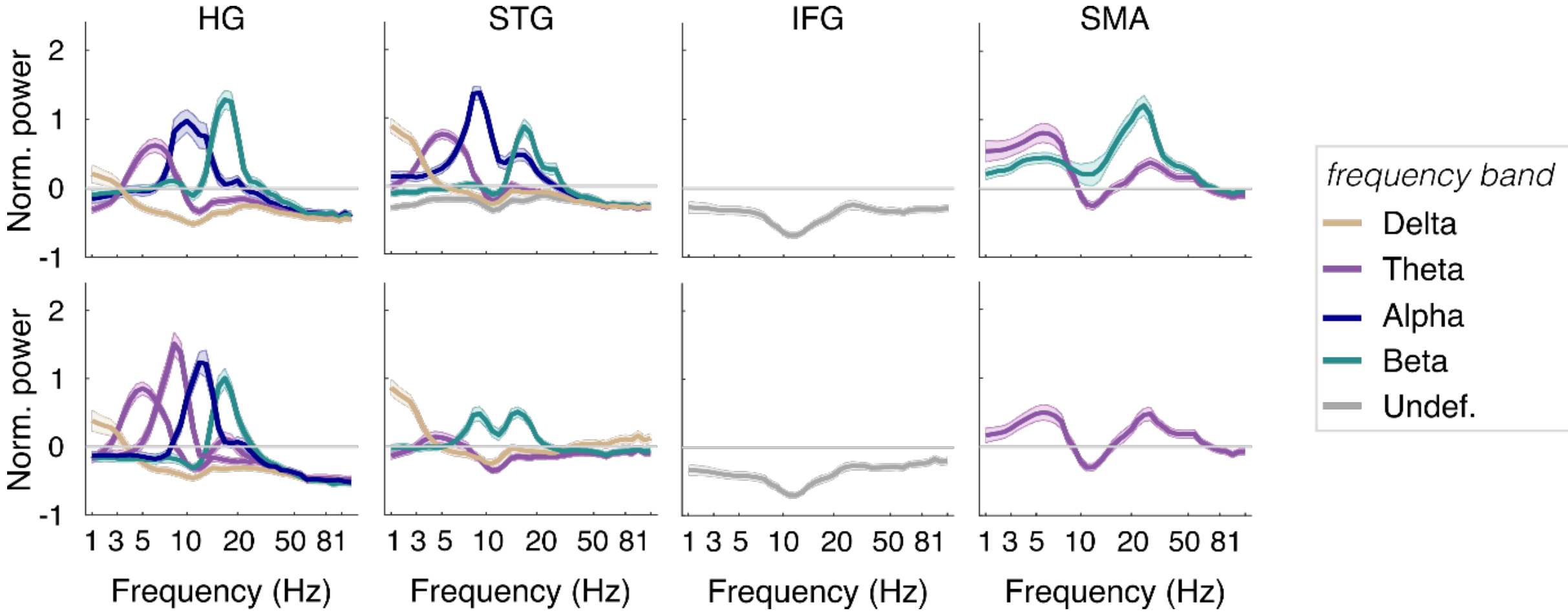

S3 Fig. Group-level spectral fingerprints in the left (top) and right (bottom) hemisphere. Clusters are color-coded according to their peak frequency (legend on the right). Shaded error bars reflect standard error of the mean across participants. HG = Heschl's gyrus; STG = superior temporal gyrus; IFG = interior frontal gyrus; SMA = supplementary motor area.
