# Supplementary material for: Endogenous auditory and motor brain rhythms predict individual speech tracking
Source: PLoS Biol. 2026 Jul 31;24(7):e3003924. doi: 10.1371/journal.pbio.3003924 (PMC13446743; doi:10.1371/journal.pbio.3003924)
Supplement: Supplementary file 4 [file pbio.3003924.s004.pdf]

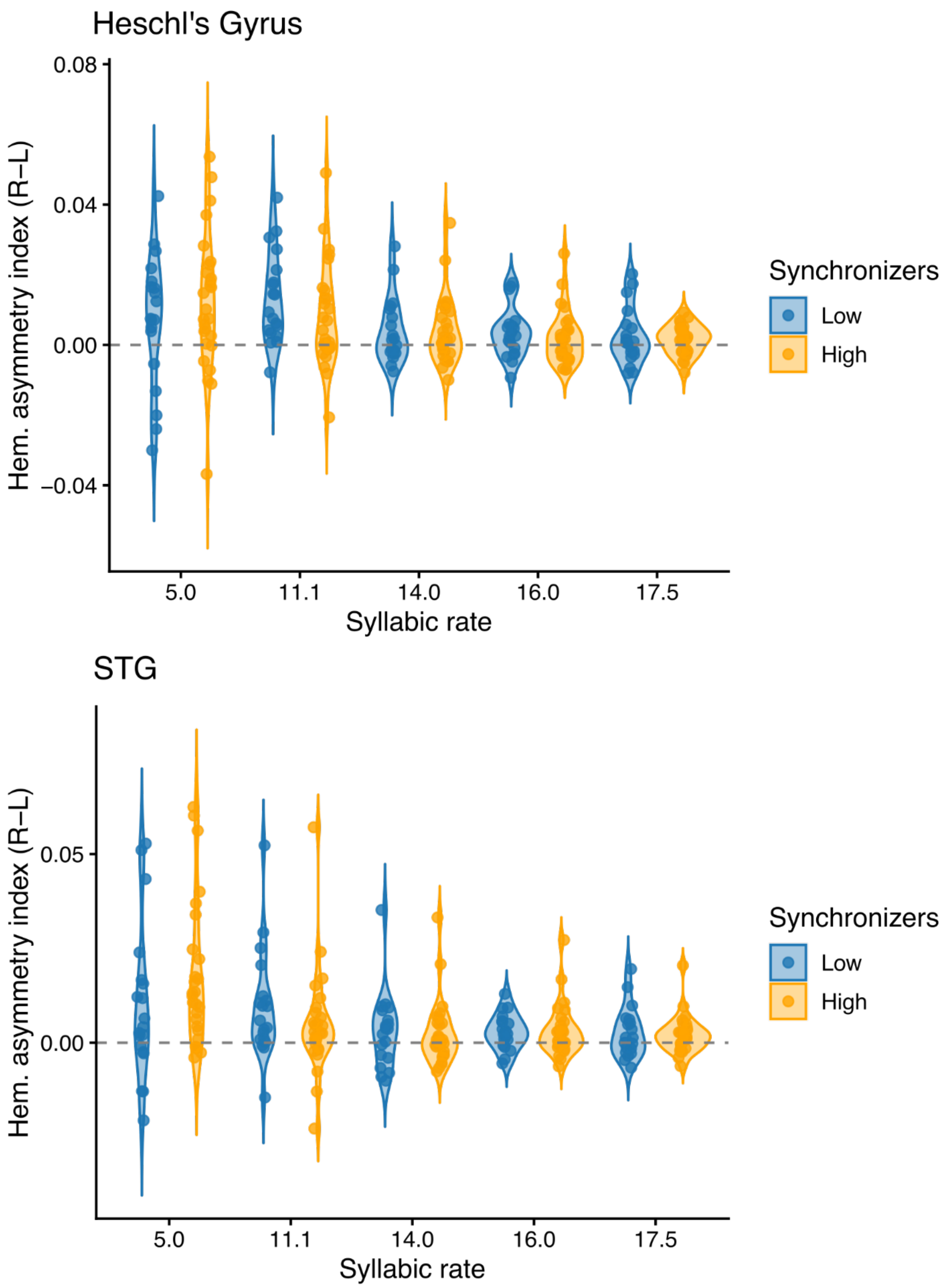

S4 Fig. Hemispheric asymmetry in speech tracking. Hemispheric asymmetry index (HAI) values are shown for high (orange) and low (blue) synchronizers across syllabic rates in Heschl's gyrus (HG; top) and posterior superior temporal gyrus (STG; bottom). Positive values indicate stronger right-hemispheric speech tracking. The underlying numerical data are available at https://doi.org/10.17605/OSF.IO/SNDPE
