# Supplementary material for: Endogenous auditory and motor brain rhythms predict individual speech tracking
Source: PLoS Biol. 2026 Jul 31;24(7):e3003924. doi: 10.1371/journal.pbio.3003924 (PMC13446743; doi:10.1371/journal.pbio.3003924)
Supplement: Supplementary file 5 [file pbio.3003924.s005.pdf]

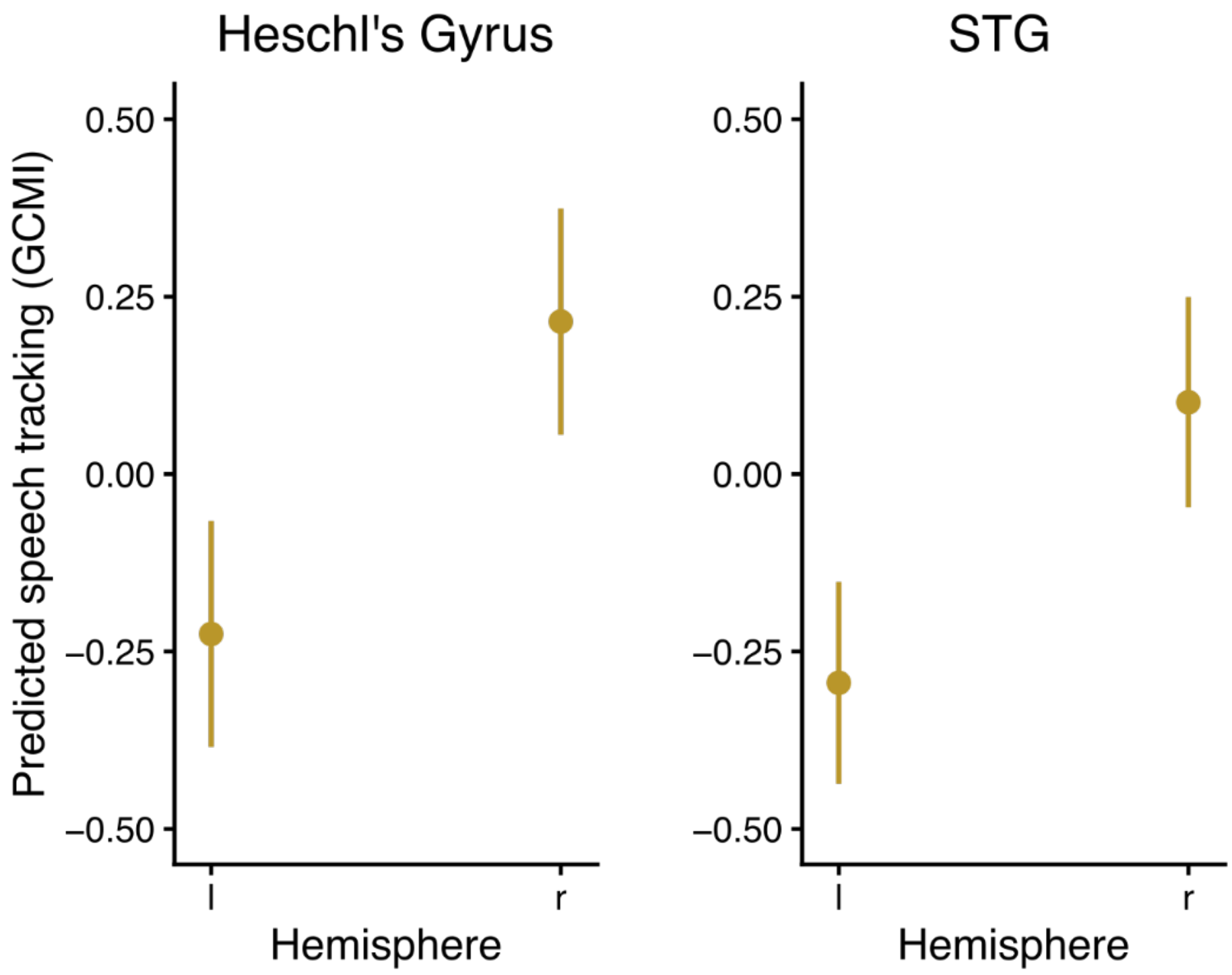

S5 Fig. Stronger speech tracking in the right HG and STG. Additional mixed models were computed, including hemisphere as categorical predictor. In both HG and STG, speech tracking was significantly stronger in the right, than the left hemisphere (see also Supplementary models 11,12). The underlying numerical data are available at https://doi.org/10.17605/OSF.IO/SNDPE
