# Supplementary material for: Endogenous auditory and motor brain rhythms predict individual speech tracking
Source: PLoS Biol. 2026 Jul 31;24(7):e3003924. doi: 10.1371/journal.pbio.3003924 (PMC13446743; doi:10.1371/journal.pbio.3003924)
Supplement: Supplementary file 6 [file pbio.3003924.s006.pdf]

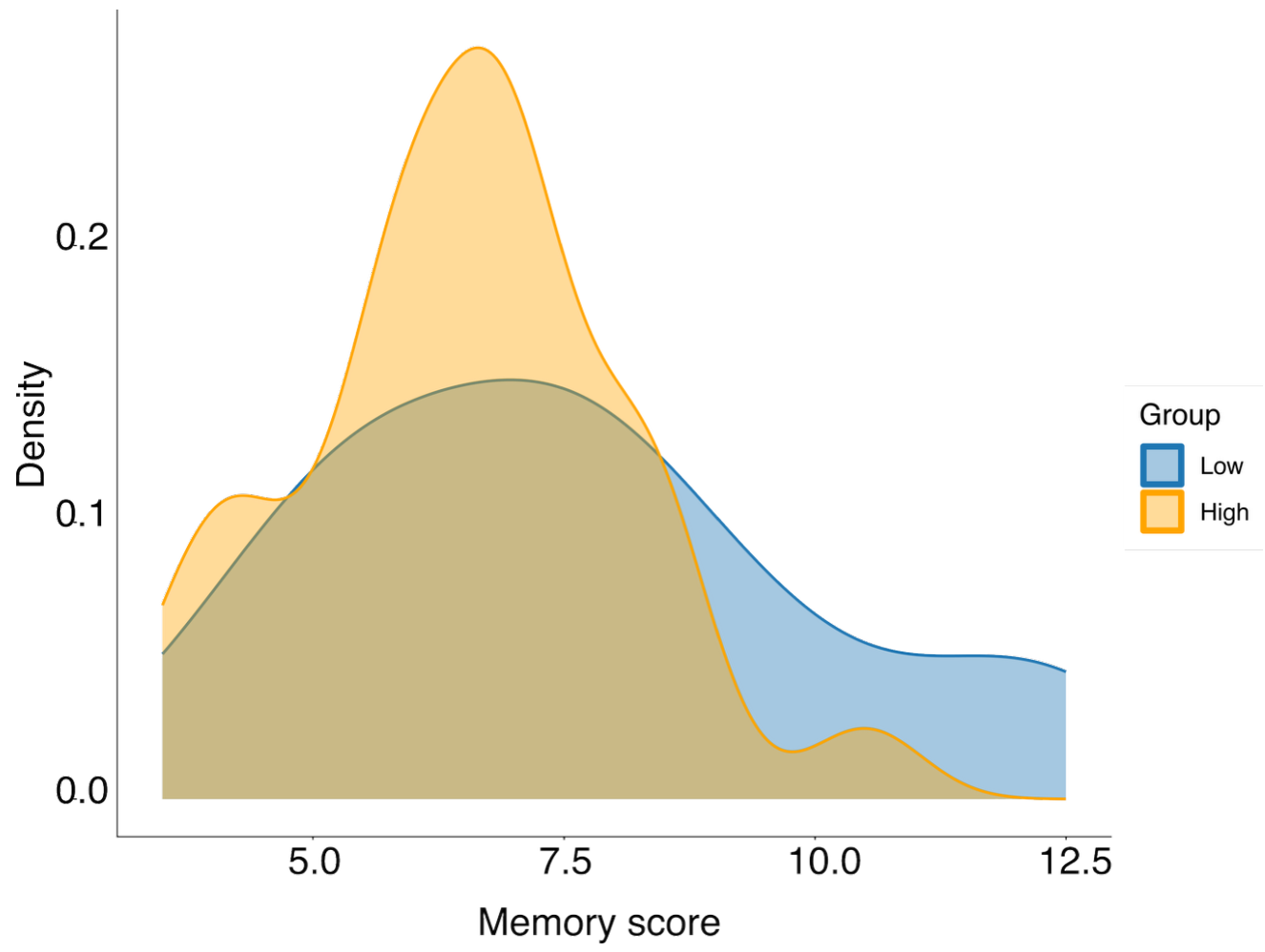

S6 Fig. Distribution of memory scores. Distribution of working memory performance scores for participants classified as high synchronizers and low synchronizers. Scores are plotted separately for each group. Working memory scores do not differ between groups, as assessed using a Wilcoxon-Test (W = 375.5, p-value = 0.188).
