# Supplementary material for: Endogenous auditory and motor brain rhythms predict individual speech tracking
Source: PLoS Biol. 2026 Jul 31;24(7):e3003924. doi: 10.1371/journal.pbio.3003924 (PMC13446743; doi:10.1371/journal.pbio.3003924)
Supplement: Supplementary file 7 [file pbio.3003924.s007.pdf]

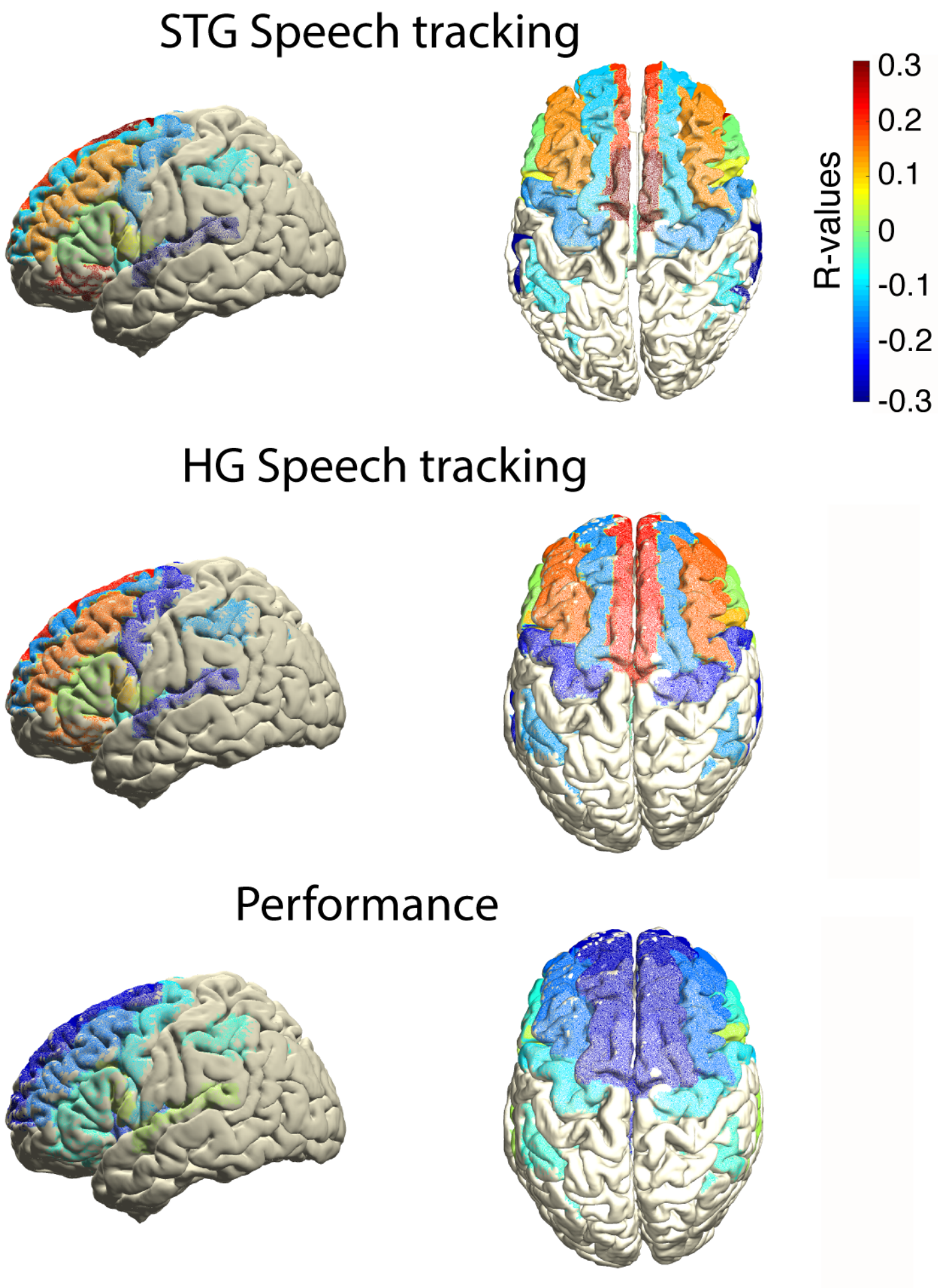

S7 Fig. Correlations between the theta brain rhythm of various regions of interest and speech tracking in STG and HG and performance in the intelligibility task. We pre-selected all brain areas from the frontal, central and temporal cortex and choose those areas that consistently showed theta cluster across the studies Keitel & Gross (2016) and Lubinus et al. (2021). Additionally, two parietal areas were chosen. If for these brain areas a theta cluster was present in a larger number of participants, the area was chosen as region of interest (ROI) for the correlational analysis. The following 15 ROIs from the AAL atlas were analyzed: Frontal_Inf_Tri_, Supp_Motor_Area_, Heschl_, Temporal_Sup_, Parietal_Inf_, Precentral_, Insula_, Rolandic_Oper_, Supra_Marginal_Gyrus_, Frontal_Inf_Oper_, Frontal_Sup_Medial_, Frontal_Mid_, Frontal_Sup_, Frontal_Inf_Orb_, Cingulum_Mid_. Peak frequencies of the theta cluster with maximum amplitude were selected for each participant. Peak frequencies were averaged across the left and right hemisphere. Spearman correlations between the peak frequencies of the endogenous brain rhythms in each ROI and the speech tracking in STG (upper row; averaged across hemispheres and syllabic rate conditions), speech tracking in HG (middle row; averaged across hemispheres and syllabic rate conditions) and performance (bottom row) were computed. False-discovery (FDR) control for multiple comparisons was applied. R-values are color coded. Although, several ROIs show significant effects, only the negative correlations of STG peak frequencies with STG speech tracking survive multiple comparison control. The underlying statistic data, displayed in this figure are available in Supplementary Table 26. The brain plots were generated using the Fieldtrip toolbox by mapping the R-values onto the respective ROIs of the AAL atlas template brain (ROI_MNI_V4.nii using: ft_readatlas) that had been interpolated to a brain surface mesh template (surface_pial_both.mat; using ft_sourceinterpolate) and plotted (using: ft_sourceplot).
