# Supplementary material for: Endogenous auditory and motor brain rhythms predict individual speech tracking
Source: PLoS Biol. 2026 Jul 31;24(7):e3003924. doi: 10.1371/journal.pbio.3003924 (PMC13446743; doi:10.1371/journal.pbio.3003924)
Supplement: Supplementary file 8 [file pbio.3003924.s008.pdf]

S1 Table. Sentence materials: Titles and authors of (audio)books. List of sources from which stimuli for the tasks (speech comprehension and preferred auditory rate tasks) were constructed.

Type	Author	Title	Source
PDF	Hedwig Dohm	Sibilla Dalmar	Zeno
PDF	Hedwig Dohm	Schicksale einer Seele	Zeno
PDF	Hedwig Dohm	Christa Ruland	Zeno
PDF	Dora Duncker	Großstadt	Zeno
PDF	Bertha von Suttner	Martha's Kinder	Zeno
PDF	Bertha von Suttner	Die Waffen nieder	Zeno
PDF	Stefan Zweig	Schachnovelle	Zeno
PDF	Ida Boy-Ed	Fanny Förster	Zeno
Audiobook	Frances Hodgson Burnett	Der kleine Lord	Librivox
Audiobook	Ludwig Thoma	Tante Frieda	Librivox
Audiobook	Ludwig Thoma	Lausbubengeschichten	Librivox
