# Supplementary material for: Endogenous auditory and motor brain rhythms predict individual speech tracking
Source: PLoS Biol. 2026 Jul 31;24(7):e3003924. doi: 10.1371/journal.pbio.3003924 (PMC13446743; doi:10.1371/journal.pbio.3003924)
Supplement: Supplementary file 9 [file pbio.3003924.s009.pdf]

S2 Table. Prompts for speech production task. Thematic questions used to facilitate natural speech production. Each item is representative of a different thematic category, as introduced by Alexandrou et al.(2016). Sentences were translated to German.

Category	Sentence/statement
Own life	Welche Hobbies haben Sie oder hatten Sie in Ihrem bisherigen Leben?<br>What kind of hobbies do you have or have had during your life?
Preferences	Welche Art von Urlaubstrips mögen Sie am liebsten?<br>What kinds of vacation trips do you like?
People	Beschreiben Sie bitte einen Künstler, Schriftsteller oder Filmdirektor. Warum finden Sie ihn/sie interessant?<br>Describe a known artist, writer, or film director. Why do you find her/him interesting?
Culture/traditions	Beschreiben Sie bitte ein traditionelles Weihnachtsfest.<br>Describe a traditional Christmas holiday.
Society/politics	Was wissen Sie über Müll- und Recyclingregelungen in Ihrem Heimatland?<br>What do you know about garbage and recycling policies in your home country?
General knowledge	Was wissen Sie über Skifahren und Snowboarden?<br>What do you know about skiing and snowboarding?
