# Supplementary material for: Endogenous auditory and motor brain rhythms predict individual speech tracking
Source: PLoS Biol. 2026 Jul 31;24(7):e3003924. doi: 10.1371/journal.pbio.3003924 (PMC13446743; doi:10.1371/journal.pbio.3003924)
Supplement: Supplementary file 10 [file pbio.3003924.s010.pdf]

S3 Table. Predicting single-trial speech comprehension performance. Syllabic rate^2: indicates the quadratic model of the syllabic rate effect; PLV: phase-locking value, indicates strength of auditory-motor synchronization behavior

Predictors	beta	SE	t	p_{FDR}	p	df	ηp²
syllabic rate	-77.72	1.91	-40.63	**<0.001**	**<0.001**	94	-
syllabic rate^2	-26.66	1.01	-26.44	**<0.001**	**<0.001**	13900	0.92
PLV	0.05	0.01	3.51	**0.001**	**<0.001**	4466	0.002
motor rate	0.02	0.02	1.20	**0.266**	0.228	4532	0.00032
auditory rate	0.1	0.02	6.18	**<0.001**	**<0.001**	44560	0.008
working memory	0.07	0.01	4.92	**<0.001**	**<0.001**	4407	0.005
compression	0	0.01	-0.54	0.587	0.587	13370	0.00001
num. syllables	-0.12	0.02	-5.19	**<0.001**	**<0.001**	402	0.06
stimulus order	0.05	0	10.97	**<0.001**	**<0.001**	16070	0.0074
PLV * motor rate	0.05	0.02	2.77	**0.007**	**0.006**	4543	0.0017
PLV * auditory rate	0.08	0.01	5.1	**<0.001**	**<0.001**	4510	0.0057
motor rate * auditory rate	-0.08	0.02	-3.58	**<0.001**	**<0.001**	4441	0.0032
PLV * motor rate * auditory rate	-0.11	0.02	-5.19	**<0.001**	**<0.001**	4506	0.0058

Random effects

Group	Name	Variance	STD
Trial-ID	(Intercept)	0.2015501	0.44894
Subjects	Syllabic rate	0.0004362	0.02089
Residual		0.2965593	0.54457
N (subs)		55	
Observations		16500	
Marginal R^2		0.478	
