# Supplementary material for: Endogenous auditory and motor brain rhythms predict individual speech tracking
Source: PLoS Biol. 2026 Jul 31;24(7):e3003924. doi: 10.1371/journal.pbio.3003924 (PMC13446743; doi:10.1371/journal.pbio.3003924)
Supplement: Supplementary file 11 [file pbio.3003924.s011.pdf]

S4 Table. Predicting single-trial speech comprehension performance, separately for high and low synchronizers. Syllabic rate^2 : indicates the quadratic model of the syllabic rate effect

	High synchronizers							Low synchronizers						
Predictors	beta	SE	t	p_{FDR}	p	df	ηp^2	beta	SE	t	p_{FDR}	p	df	ηp^2
syllabic rate	-16.86	0.53	-31.76	**<0.001**	**<0.001**	55	**-**	-14.78	0.73	-20.26	**<0.001**	**<0.001**	28	-
syllabic rate^2	-6.21	0.31	-20.15	**<0.001**	**<0.001**	6463	0.92	-5.21	0.3	-17.27	**<0.001**	**<0.001**	4791	0.93
motor rate	0.03	0.01	3.73	**<0.001**	**<0.001**	1370	0.01	-0.01	0.01	-1.13	**<0.001**	0.331	2995	0.0004
auditory rate	0.03	0.01	2.73	**0.010**	**0.006**	1343	0.008	-0.01	0.01	-1.03	0.337	**<0.001**	3011	0.0004
working memory	-0.01	0.01	-0.76	0.506	0.449	1371	0.0004	0.03	0.01	5.66	0.337	**<0.001**	2832	0.01
compression	0	0	0.50	0.621	0.621	6204	0.00004	0	0	-0.89	**<0.001**	0.504	4528	0.0002
num. syllables	-0.04	0.01	-4.82	**<0.001**	<0.001	409	0.05	-0.04	0.01	-4.77	0.374	<0.001	412	0.05
stimulus order	0.01	0	4.74	**<0.001**	<0.001	7727	0.003	0.02	0	9.39	**<0.001**	<0.001	5953	0.010
Motor rate * auditory rate	-0.02	3.01	-2.60	**0.013**	**0.009**	1362	0.005	0.01	0.01	1.08	**<0.001**	0.709	3024	0.0004

Random effects

		High synchronizers		Low synchronizers	
Group	Name	Variance	STD	Variance	STD
Trial-ID	(Intercept)	2.04E-02	0.142725	0.0197654	0.140589
Subjects	Syllabic rate	3.07E-05	0.005542	0.0000746	0.008637
Residual		2.87E-02	0.169437	0.030909	0.17581
N (subs)		27		21	
Observations		8100		6300	
Marginal R^2		0.471		0.481	
