# Supplementary material for: Endogenous auditory and motor brain rhythms predict individual speech tracking
Source: PLoS Biol. 2026 Jul 31;24(7):e3003924. doi: 10.1371/journal.pbio.3003924 (PMC13446743; doi:10.1371/journal.pbio.3003924)
Supplement: Supplementary file 12 [file pbio.3003924.s012.pdf]

S5 Table. HG general model: Predicting speech tracking in HG. Syllabic rate^2 : indicates the quadratic model of the syllabic rate effect

Predicted speech tracking in HG

Predictors	beta	SE	t	p_{FDR}	p	df	ηp^2
syllabic rate	-9.27	0.8	-11.53	**<0.001**	**<0.001**	254	**-**
syllabic rate^2	0.14	0.78	0.18	0.981	0.858	206	0.37
SP Heschlm	-0.04	0.07	-0.65	0.72	0.514	61	0.007
SP SMAm	0.22	0.07	3.01	**0.023**	**0.003**	69	0.12
couplingSMA HG	-0.06	0.06	-1.03	0.626	0.305	142	0.007
SP IFGm	-0.11	0.08	-1.5	0.429	0.134	64	0.03
couplingIFG HG	0	0.06	0.02	0.984	0.984	122	< 0.0001
SP Heschlm × SP SMAm	0.11	0.07	1.56	0.429	0.119	64	0.04
SP Heschlm × couplingSMA HG	-0.04	0.06	-0.59	0.72	0.553	129	0.003
SP SMAm × couplingSMA HG	0.07	0.08	0.9	0.626	0.366	71	0.004
SP Heschlm × SP IFGm	-0.07	0.09	-0.86	0.626	0.391	167	0.01
SP Heschlm × couplingIFG HG	0.04	0.08	0.55	0.72	0.585	125	0.002
SP IFGm × couplingIFG HG	-0.08	0.07	-1.14	0.626	0.254	125	0.01
(SP Heschlm × SP SMAm) × couplingSMA HG	0	0.08	-0.05	0.984	0.958	233	< 0.0001
(SP Heschlm × SP IFGm) x couplingIFG HG	0.19	0.08	2.39	0.094	**0.018**	175	0.03

Random effects

Group	Name	Variance	STD
Subjects	Syllabic rate	0.000401	0.02002
Residual		0.599258	0.77412
N (subs)		54	
Observations		270	
Marginal R^2		0.4	
