# Supplementary material for: Endogenous auditory and motor brain rhythms predict individual speech tracking
Source: PLoS Biol. 2026 Jul 31;24(7):e3003924. doi: 10.1371/journal.pbio.3003924 (PMC13446743; doi:10.1371/journal.pbio.3003924)
Supplement: Supplementary file 13 [file pbio.3003924.s013.pdf]

S6 Table. Predicting speech tracking in HG, including behaviorally measured auditory-motor synchronization (PLV) as predictor. Syllabic rate^2: indicates the quadratic model of the syllabic rate effect; PLV: phase-locking value, indicates strength of auditory-motor synchronization behavior

Predicted speech tracking in HG

Predictors	beta	SE	t	p_{FDR}	p	df	ηp²
syllabic rate	-9.26	0.79	-11.7	**<0.001**	**<0.001**	250	**-**
syllabic rate^2	0.11	0.78	0.14	0.989	0.886	205	0.38
SP Heschlm	0.01	0.07	0.07	0.989	0.942	54	<0.0001
SP SMA	-0.18	0.15	-1.2	0.398	0.23	57	0.02
SMA-HG coupling	-0.04	0.06	-0.75	0.668	0.457	122	0.005
SP IFG	0.03	0.06	0.4	0.817	0.688	54	0.03
IFG-HG coupling	0.19	0.16	1.21	0.398	0.229	105	0.002
PLV	0.58	0.34	1.71	0.241	0.089	50	0.06
SP HG × SP SMA	0.12	0.07	1.78	0.24	0.076	57	0.05
SP HG x SMA-HG coupling	-0.03	0.06	-0.57	0.725	0.572	114	0.003
SP SMA ×SMA-HG coupling	0.05	0.08	0.87	0.698	0.514	203	0.002
SP HG × SP IFG	-0.11	0.09	-1.27	0.398	0.206	62	0.03
SP HG x IFG-HG coupling	0.09	0.07	1.14	0.403	0.254	148	0.009
SP IFG x IFG-HG coupling	-0.09	0.07	-1.39	0.391	0.164	106	0.02
SP SMA x PLV	0.77	0.29	2.62	0.089	**0.009**	53	0.11
SP IFG x PLV	-0.62	0.33	-1.9	0.224	0.059	51	0.07
(SP HG x SP SMA) x SMA HG coupling	-0.00	0.07	-0.01	0.989	0.989	216	< 0.001
(SP HG x SP IFG) x IFG HG coupling	0.19	0.08	2.44	0.098	**0.016**	153	0.04

Random effects

Group	Name	Variance	STD
Subjects	Syllabic rate	0.0002493	0.01579
Residual		0.5941521	0.77081
N (subs)		54	
Observations		270	
Marginal R^2		0.415	
