# Supplementary material for: Endogenous auditory and motor brain rhythms predict individual speech tracking
Source: PLoS Biol. 2026 Jul 31;24(7):e3003924. doi: 10.1371/journal.pbio.3003924 (PMC13446743; doi:10.1371/journal.pbio.3003924)
Supplement: Supplementary file 14 [file pbio.3003924.s014.pdf]

S7 Table. Group models: Predicting speech tracking in HG, separately for high and low synchronizers. Syllabic rate^2 : indicates the quadratic model of the syllabic rate effect

Predicted speech tracking in HG

	High synchronizers							Low synchronizers						
Predictors	beta	SE	t	p_{FDR}	p	df	ηp^2	beta	SE	t	p_{FDR}	p	df	ηp^2
syllabic rate	-6.56	0.81	-8.15	**<0.001**	**<0.001**	96		-6.03	0.75	-8.04	**<0.001**	**<0.001**	83	-
syllabic rate^2	0.14	0.72	0.20	0.892	0.843	81	0.43	0.36	0.74	0.48	0.779	0.663	56	0.49
HG	0.12	0.12	1.01	0.612	0.315	19	0.05	-0.18	0.14	-1.33	0.476	0.186	5	0.25
SMA	0.28	0.13	2.15	0.180	**0.034**	20	0.19	0.14	0.10	1.36	0.476	0.177	6	0.24
SMA-HG coupling	-0.09	0.10	-0.88	0.612	0.383	91	0.008	-0.08	0.12	-0.69	0.714	0.491	21	0.02
IFG	-0.44	0.14	-3.02	**0.025**	**0.003**	20	0.31	-0.04	0.13	-0.34	0.840	0.735	7	0.02
IFG-HG coupling	0.05	0.11	0.45	0.782	0.654	60	0.003	0.08	0.10	0.79	0.687	0.429	9	0.07
HG × SMA	0.29	0.14	2.01	0.187	**0.047**	23	0.15	-0.09	0.10	-0.92	0.640	0.360	5	0.14
HG × SMA-HG coupling	0.01	0.10	0.14	0.892	0.892	80	0.0002	0.00	0.10	0.01	0.992	0.992	12	<.0001
SMA × SMA-HG coupling	-0.08	0.13	-0.58	0.751	0.563	121	0.003	0.12	0.13	0.97	0.640	0.333	43	0.02
HG × IFG	0.12	0.14	0.88	0.612	0.381	24	0.03	-0.69	0.18	-3.90	**0.002**	**<0.001**	5	0.76
HG × IFG-HG coupling	0.08	0.12	0.72	0.691	0.475	72	0.007	-0.09	0.16	-0.57	0.760	0.570	31	0.01
IFG × IFG-HG coupling	-0.17	0.13	-1.28	0.543	0.204	59	0.03	-0.02	0.09	-0.20	0.900	0.844	9	0.004
(HG × SMA) × SMA-HG coupling	-0.05	0.13	-0.41	0.782	0.685	116	0.001	0.16	0.10	1.66	0.401	0.100	15	0.15
(HG × IFG) × IFG-HG coupling	0.11	0.10	1.14	0.582	0.254	79	0.02	0.22	0.18	1.27	0.476	0.208	26	0.06

Random effects

		High synchronizers		Low synchronizers	
Group	Name	Variance	STD	Variance	STD
Subjects	Syllabic rate	0.001891	0.04348	0.000	0.000
Residual		0.492249	0.70160	0.8133	0.9018
N (subs)		28		20	
Observations		140		100	
Marginal R^2		0.557		0.511	
