# Supplementary material for: Endogenous auditory and motor brain rhythms predict individual speech tracking
Source: PLoS Biol. 2026 Jul 31;24(7):e3003924. doi: 10.1371/journal.pbio.3003924 (PMC13446743; doi:10.1371/journal.pbio.3003924)
Supplement: Supplementary file 15 [file pbio.3003924.s015.pdf]

S8 Table. STG general model: Predicting speech tracking in STG. Syllabic rate² : indicates the quadratic model of the syllabic rate effect

Predicted speech tracking in STG

Predictors	beta	SE	t	p_{FDR}	p	df	ηp²
syllabic rate	-8.72	0.83	-10.51	**<0.001**	**<0.001**	233	**-**
syllabic rate²	1.57	0.8	1.96	0.205	0.051	190	0.353
STG	-0.18	0.06	-3.24	**0.011**	**0.001**	51	0.17
SMA-STG coupling	0.11	0.06	1.85	0.208	0.065	46	0.07
SMA	-0.05	0.05	-0.86	0.69	0.388	89	0.008
IFG-STG coupling	-0.01	0.06	-0.18	0.947	0.859	48	0.0007
IFG	0.12	0.06	2.08	0.205	**0.039**	96	0.04
STG × SMA-STG coupling	-0.07	0.06	-1.11	0.607	0.269	55	0.02
STG × SMA	-0.01	0.06	-0.17	0.947	0.868	142	0.0002
SMA × SMA-STG coupling	-0.06	0.06	-1.03	0.607	0.304	126	0.008
STG × IFG-STG coupling	0	0.07	-0.07	0.947	0.947	51	<.0001
STG × IFG	-0.02	0.06	-0.4	0.947	0.691	127	0.001
IFG × IFG-STG coupling	-0.07	0.06	-1.16	0.607	0.247	124	0.01
(STG × SMA) × SMA-STG coupling	0.03	0.06	0.51	0.947	0.611	200	0.001
(STG × IFG) × IFG-STG coupling	0.01	0.07	0.18	0.947	0.857	129	0.0003

Random effects

Group	Name	Variance	STD
Subjects	Syllabic rate	4.42E-06	0.002102
Residual		6.35E-01	0.796768
N (subs)		50	
Observations		250	
Marginal R²		0.388	
