# Supplementary material for: Endogenous auditory and motor brain rhythms predict individual speech tracking
Source: PLoS Biol. 2026 Jul 31;24(7):e3003924. doi: 10.1371/journal.pbio.3003924 (PMC13446743; doi:10.1371/journal.pbio.3003924)
Supplement: Supplementary file 16 [file pbio.3003924.s016.pdf]

S9 Table. Predicting speech tracking in STG, including behaviorally measured auditory-motor synchronization (PLV) as predictor. Syllabic rate^2: indicates the quadratic model of the syllabic rate effect; PLV: phase-locking value, indicates strength of auditory-motor synchronization behavior

Predicted speech tracking in STG

Predictors	beta	SE	t	p_{FDR}	p	df	ηp²
**syllabic rate**	-8.78	0.81	-10.82	**<0.001**	**<0.001**	230	-
syllabic rate^2	1.58	0.78	2.02	0.142	**0.045**	186	0.37
**STG**	-0.19	0.06	-3.41	**0.005**	**0.011**	45	0.20
SMA	-0.11	0.15	-0.77	0.702	0.444	43	0.01
SMA-STG coupling	-0.03	0.05	-0.52	0.722	0.604	82	0.003
**IFG**	0.44	0.14	3.23	**0.007**	**0.001**	41	0.20
IFG-STG coupling	0.13	0.06	2.16	0.12	**0.032**	87	0.05
PLV	0.14	0.28	-0.51	0.722	0.610	41	0.006
STG × SMA	-0.05	0.07	-0.78	0.702	0.436	44	0.01
STG × SMA-STG coupling	-0.02	0.06	-0.40	0.730	0.692	137	0.001
SMA x SMA-STG coupling	-0.08	0.06	-1.41	0.338	0.160	118	0.02
STG × IFG	-0.00	0.07	-0.00	1.000	1.000	45	<.001
STG × IFG-STG coupling	-0.03	0.06	-1.63	0.281	0.646	114	0.002
IFG x IFG-STG coupling	-0.10	0.06	-1.63	0.281	0.104	109	0.02
PLV x SMA	0.43	0.27	1.57	0.281	0.118	42	0.06
**PLV x IFG**	-1.01	0.28	-3.64	**0.003**	**<0.001**	39	0.25
(STG × SMA) × SMA-STG coupling	0.06	0.06	0.84	0.702	0.383	196	0.004
(STG × IFG) × IFG-STG coupling	0.03	0.07	0.87	0.722	0.633	119	0.002

Random effects

Group	Name	Variance	STD
Subjects	Syllabic rate	0.000818	0.0286
Residual		0.589251	0.7676
N (subs)		50	
Observations		250	
Marginal R^2		0.421	
