# Supplementary material for: Endogenous auditory and motor brain rhythms predict individual speech tracking
Source: PLoS Biol. 2026 Jul 31;24(7):e3003924. doi: 10.1371/journal.pbio.3003924 (PMC13446743; doi:10.1371/journal.pbio.3003924)
Supplement: Supplementary file 17 [file pbio.3003924.s017.pdf]

S10 Table. Group models: Predicting speech tracking in STG, separately for high and low synchronizers. Syllabic rate² : indicates the quadratic model of the syllabic rate effect

Predicted speech tracking in STG

	High synchronizers							Low synchronizers						
Predictors	beta	SE	t	p_{FDR}	p	df	ηp^2	beta	SE	t	p_{FDR}	p	df	ηp^2
syllabic rate	-6.50	0.77	-8.43	**<0.001**	**<0.001**	111	-	-5.57	0.81	-6.88	**<0.001**	**<0.001**	66	-
syllabic rate²	1.53	0.74	2.09	0.128	**0.04**	102	0.42	0.87	0.73	1.19	0.508	0.237	49	0.46
STG	-0.16	0.09	-1.91	0.135	0.058	21	0.15	-0.49	0.14	-3.43	**0.008**	**0.001**	6	0.67
SMA	0.26	0.09	3.05	**0.015**	**0.003**	18	0.34	0.21	0.13	1.58	0.477	0.119	6	0.29
SMA-STG coupling	0.06	0.08	0.81	0.519	0.422	38	0.02	-0.14	0.11	-1.34	0.504	0.183	55	0.03
IFG	-0.15	0.08	-1.91	0.135	0.059	17	0.17	0.30	0.13	2.24	0.149	**0.028**	7	0.41
IFG-STG coupling	0.10	0.08	1.30	0.390	0.195	58	0.03	0.10	0.15	0.70	0.555	0.486	53	0.009
STG × SMA	-0.23	0.09	-2.57	**0.046**	**0.012**	17	0.28	0.23	0.19	1.19	0.504	0.238	7	0.17
STG × SMA-STG coupling	-0.05	0.09	-0.48	0.670	0.629	84	0.003	0.09	0.12	0.71	0.555	0.477	68	0.007
SMA × SMA-STG coupling	-0.07	0.09	-0.74	0.527	0.461	43	0.01	-0.12	0.12	-1.00	0.521	0.321	53	0.02
STG × IFG	-0.10	0.10	-0.95	0.519	0.346	24	0.04	-0.13	0.18	-0.71	0.555	0.482	7	0.07
STG × IFG-STG coupling	-0.03	0.08	-0.43	0.670	0.67	95	0.002	0.16	0.16	0.99	0.521	0.326	66	0.01
IFG × IFG-STG coupling	-0.24	0.08	-3.05	**0.015**	**0.003**	73	0.11	-0.04	0.13	-0.29	0.778	0.771	55	0.002
(STG × SMA) × SMA-STG coupling	0.08	0.10	0.82	0.519	0.413	113	0.009	0.04	0.13	0.28	0.778	0.778	71	0.001
(STG × IFG) × IFG-STG coupling	0.10	0.10	1.04	0.519	0.302	110	0.01	0.13	0.14	0.90	0.536	0.368	42	0.02

Random effects

		High synchronizers		Low synchronizers
Group	Name	Variance	STD	Variance
Subjects	(Intercept)	0.03455	0.1859	0.000
Residual		0.51855	0.7201	0.6032
N (subs)		27		19
Observations		135		95
Marginal R^2		0.484		0.542

				c	
Group	Name	Variance	STD	Variance	STD
Subjects	Syllabic rate	**0.52**		0.000	0.000
Residual		**0.03**		0.6032	0.7767
N (subs)		27		19	
Observations		135		95	
Marginal R^2		0.484		0.542	
