# Supplementary material for: Endogenous auditory and motor brain rhythms predict individual speech tracking
Source: PLoS Biol. 2026 Jul 31;24(7):e3003924. doi: 10.1371/journal.pbio.3003924 (PMC13446743; doi:10.1371/journal.pbio.3003924)
Supplement: Supplementary file 18 [file pbio.3003924.s018.pdf]

S11 Table. Predicting speech tracking in STG, including a categorical predictor for hemisphere, both as main effect and as interaction term with the endogenous frequencies of STG, SMA, IFG. Syllabic rate² : indicates the quadratic model of the syllabic rate effect

Predictors	*Estimates*	*SE*	*Statistic*	p_{FDR}	*p*	*df*	ηp²
syllabic rate	-10.07	0.86	-11.69	**<0.001**	**<0.001**	508	**-**
syllabic rate²	1.81	0.83	2.17	0.182	**0.03**	471	0.24
SP STG	-0.11	0.07	-1.71	0.251	0.087	214	0.04
SP SMA	0.12	0.07	1.82	0.251	0.07	220	0.03
SMA STG	0.03	0.06	0.59	0.757	0.555	406	0.002
hemisphere [r]	0.39	0.08	4.64	**<0.001**	**<0.001**	515	0.04
SP IFG tri	-0.05	0.07	-0.74	0.74	0.458	227	0.002
IFG STG	0.13	0.06	2.03	0.184	**0.043**	431	0.005
SP STG × SP SMA	-0.21	0.08	-2.74	**0.049**	**0.006**	208	0.04
SP STG × SMA STG	0.02	0.07	0.37	0.929	0.712	431	0.0001
SP SMA × SMA STG	-0.02	0.07	-0.28	0.963	0.783	474	0.001
SP STG × hemisphere [r]	0	0.09	0.04	0.971	0.967	246	0.0006
SP SMA × hemisphere [r]	-0.01	0.09	-0.15	0.971	0.882	292	0.0005
SMA STG × hemisphere [r]	-0.17	0.08	-2.06	0.184	**0.04**	446	0.01
SP STG × SP IFG tri	0.06	0.07	0.91	0.667	0.365	256	0.003
SP STG × IFG STG	0	0.06	-0.07	0.971	0.944	435	0.002
SP IFG tri × IFG STG	-0.09	0.06	-1.4	0.371	0.161	415	0.001
hemisphere [r] × SP IFG tri	0.14	0.09	1.63	0.258	0.103	239	0.01
hemisphere [r] × IFG STG	-0.07	0.11	-0.65	0.74	0.518	390	0.0006
(SP STG × SP SMA) × SMA STG	0	0.09	0.04	0.971	0.971	457	0.0008
(SP STG × SP SMA) × hemisphere [r]	0.12	0.1	1.16	0.529	0.247	185	0.008
(SP STG × SMA STG) × hemisphere [r]	-0.01	0.09	-0.14	0.971	0.89	423	<.0001
(SP SMA × SMA STG) × hemisphere [r]	-0.03	0.11	-0.25	0.963	0.803	452	0.0001
(SP STG × SP IFG tri) × IFG STG	0.06	0.07	0.88	0.667	0.378	447	0.02
(SP STG × hemisphere [r]) × SP IFG tri	-0.07	0.1	-0.67	0.74	0.502	185	0.002
(SP STG × hemisphere [r]) × IFG STG	0.1	0.11	0.92	0.667	0.359	439	0.002
(hemisphere [r] × SP IFG tri) × IFG STG	0.18	0.1	1.71	0.251	0.088	395	0.01
(SP STG × SP SMA × SMA STG) × hemisphere [r]	-0.09	0.12	-0.72	0.74	0.471	427	0.001
(SP STG × hemisphere [r] × SP IFG tri) × IFG STG	0.19	0.11	1.69	0.251	0.092	447	0.007

Random effects:

Groups	Name	Variance	Std.Dev.
sub	freq	2.68E-05	0.005176
Residual		6.88E-01	0.829293

Number of obs: 500, groups: sub, 50

Marginal R^2: 0.327
