# Supplementary material for: Endogenous auditory and motor brain rhythms predict individual speech tracking
Source: PLoS Biol. 2026 Jul 31;24(7):e3003924. doi: 10.1371/journal.pbio.3003924 (PMC13446743; doi:10.1371/journal.pbio.3003924)
Supplement: Supplementary file 19 [file pbio.3003924.s019.pdf]

S12 Table. Predicting speech tracking in HG, including a categorical predictor for hemisphere, both as main effect and as interaction term with the endogenous frequencies of HG, SMA, IFG. Syllabic rate^2 : indicates the quadratic model of the syllabic rate effect;

Predictors	*Estimates*	*SE*	*Statistic*	*pFDR*	*p*	*df*	*ηp²*
syllabic rate	-10.34	0.87	-11.87	**<0.001**	**<0.001**	473	**-**
syllabic rate^2	0.58	0.84	0.7	0.807	0.485	469	0.24
SP Heschl	-0.07	0.08	-0.92	0.807	0.358	314	0.001
SP SMA	0.07	0.07	0.91	0.807	0.362	333	0.001
SMA HG	-0.02	0.07	-0.34	0.807	0.734	460	0.001
hemisphere [r]	0.41	0.1	4.19	**<0.001**	**<0.001**	511	0.04
SP IFG tri	-0.05	0.08	-0.71	0.807	0.479	253	0.0007
IFG HG	0.02	0.06	0.37	0.807	0.713	430	<.0001
SP Heschl × SP SMA	0.05	0.07	0.66	0.807	0.511	310	0.01
SP Heschl × SMA HG	-0.01	0.09	-0.15	0.88	0.88	502	0.0001
SP SMA × SMA HG	-0.02	0.07	-0.31	0.807	0.753	496	0.003
SP Heschl × hemisphere[r]	0.15	0.11	1.38	0.807	0.168	367	0.007
SP SMA × hemisphere [r]	-0.03	0.1	-0.26	0.82	0.793	317	0.0002
SMA HG × hemisphere [r]	-0.04	0.11	-0.33	0.807	0.743	506	0.001
SP Heschl × SP IFG tri	0.08	0.08	1.03	0.807	0.302	332	0.003
SP Heschl × IFG HG	0.03	0.07	0.37	0.807	0.71	440	0.003
SP IFG tri × IFG HG	-0.04	0.07	-0.55	0.807	0.58	376	0.003
hemisphere [r] × SP IFG tri	0.08	0.1	0.83	0.807	0.409	313	0.004
hemisphere [r] × IFG HG	-0.05	0.1	-0.51	0.807	0.613	470	0.0004
(SP Heschl × SP SMA) × SMA HG	0.03	0.08	0.43	0.807	0.669	496	0.0002
(SP Heschl × SP SMA) × hemisphere [r]	0.12	0.1	1.18	0.807	0.239	309	0.002
(SP Heschl × SMA HG) × hemisphere [r]	0.1	0.11	0.89	0.807	0.376	504	0.004
(SP SMA × SMA HG) × hemisphere [r]	-0.13	0.13	-1.01	0.807	0.313	479	0.003
(SP Heschl × SP IFG tri) × IFG HG	0.08	0.07	1.08	0.807	0.28	408	0.002
(SP Heschl × hemisphere[r]) × SP IFG tri	-0.06	0.11	-0.55	0.807	0.579	232	0.003
(SP Heschl × hemisphere[r]) × IFG HG	-0.17	0.11	-1.58	0.807	0.116	420	0.005
(hemisphere [r] × SP IFG tri) × IFG HG	-0.03	0.1	-0.33	0.807	0.743	427	0.0006
(SP Heschl × SP SMA × SMA HG) × hemisphere [r]	-0.06	0.11	-0.55	0.807	0.584	468	0.0004
(SP Heschl × hemisphere[r] × SP IFG tri) × IFG HG	-0.04	0.1	-0.39	0.807	0.7	431	0.0002

Random effects:

Groups	Name	Variance	Std.Dev.
sub	freq	0.0002957	0.0172
Residual		0.6902168	0.8308

Number of obs: 520, groups: sub, 54

Marginal R^2 0.308
