# Supplementary material for: Endogenous auditory and motor brain rhythms predict individual speech tracking
Source: PLoS Biol. 2026 Jul 31;24(7):e3003924. doi: 10.1371/journal.pbio.3003924 (PMC13446743; doi:10.1371/journal.pbio.3003924)
Supplement: Supplementary file 20 [file pbio.3003924.s020.pdf]

S13 Table. Predicting speech tracking in STG, including a categorical predictor for hemisphere and PLV (behavioral measure of auditory-motor synchronization), both as main effects and as interaction term PLV X hemisphere. Syllabic rate^2 : indicates the quadratic model of the syllabic rate effect; PLV: phase-locking value, indicates strength of auditory-motor synchronization behavior

Predictors	*Estimates*	*SE*	*t*	p_{FDR}	*p*	*df*	ηp^2
syllabic rate	-10.08	0.86	-11.68	**<0.001**	**<0.001**	504	-
syllabic rate^2	1.81	0.83	2.17	0.195	**0.03**	468	0.24
PLV	0	0.06	-0.07	0.977	0.942	184	0.001
hemisphere [r]	0.4	0.08	4.67	**<0.001**	**<0.001**	512	0.04
SP STG	-0.11	0.07	-1.67	0.281	0.096	212	0.04
SP SMA	0.12	0.07	1.8	0.281	0.072	217	0.03
SMA STG	0.03	0.06	0.58	0.823	0.56	405	0.003
SP IFG tri	-0.05	0.07	-0.71	0.763	0.477	224	0.003
IFG STG	0.13	0.06	2.03	0.204	**0.043**	431	0.005
PLV × hemisphere [r]	0.06	0.08	0.75	0.763	0.452	513	0.002
SP STG × SP SMA	-0.22	0.08	-2.66	0.065	**0.008**	206	0.04
SP STG × SMA STG	0.02	0.07	0.35	0.951	0.723	433	<.0001
SP SMA × SMA STG	-0.02	0.07	-0.25	0.951	0.803	473	0.001
hemisphere [r] × SP STG	0.01	0.09	0.11	0.977	0.909	241	0.0009
hemisphere [r] × SP SMA	-0.02	0.09	-0.26	0.951	0.794	281	0.0009
hemisphere [r] × SMA STG	-0.17	0.08	-2.01	0.204	**0.045**	446	0.01
SP STG × SP IFG tri	0.06	0.07	0.89	0.719	0.376	254	0.004
SP STG × IFG STG	0	0.06	-0.05	0.977	0.957	435	0.002
SP IFG tri × IFG STG	-0.09	0.06	-1.42	0.382	0.155	414	0.0006
hemisphere [r] × SP IFG tri	0.15	0.09	1.69	0.281	0.092	237	0.01
hemisphere [r] × IFG STG	-0.06	0.11	-0.57	0.823	0.566	389	0.0006
(SP STG × SP SMA) × SMA STG	0	0.09	0.03	0.977	0.977	457	0.0009
(hemisphere [r] × SP STG) × SP SMA	0.13	0.11	1.2	0.525	0.23	186	0.01
(hemisphere [r] × SP STG) × SMA STG	-0.02	0.09	-0.21	0.951	0.832	423	<.0001
(hemisphere [r] × SP SMA) × SMA STG	-0.03	0.11	-0.27	0.951	0.789	452	0.0001
(SP STG × SP IFG tri) × IFG STG	0.06	0.07	0.88	0.719	0.382	447	0.02
(hemisphere [r] × SP STG) × SP IFG tri	-0.04	0.1	-0.39	0.951	0.696	178	0.0007
(hemisphere [r] × SP STG)	0.11	0.11	0.94	0.719	0.349	441	0.002
× IFG STG	0.19	0.1	1.94	0.199	0.053	400	0.009
(hemisphere [r] × SP IFG tri) × IFG STG	0.17	0.11	1.59	0.298	0.112	426	0.001
(hemisphere [r] × SP STG× SP SMA) × SMA STG	-0.09	0.12	-0.75	0.763	0.454	449	0.007

Random effects:

Groups	Name	Variance	Std.Dev.
sub	freq	5.02E-05	0.007084
Residual		6.86E-01	0.828274

Number of obs: 500, groups: sub, 50

Marginal R^2 0.328
