# Supplementary material for: Endogenous auditory and motor brain rhythms predict individual speech tracking
Source: PLoS Biol. 2026 Jul 31;24(7):e3003924. doi: 10.1371/journal.pbio.3003924 (PMC13446743; doi:10.1371/journal.pbio.3003924)
Supplement: Supplementary file 21 [file pbio.3003924.s021.pdf]

S14 Table. Predicting speech tracking in HG, including a categorical predictor for hemisphere and PLV (behavioral measure of auditory-motor synchronization), both as main effects and as interaction term PLV X hemisphere. Syllabic rate^2 : indicates the quadratic model of the syllabic rate effect; PLV: phase-locking value, indicates strength of auditory-motor synchronization behavior

Predictors	*Estimates*	*std. Error*	*t*	*p FDR*	*p*	*df*	ηp²
syllabic rate	-10.33	0.87	-11.87	**<0.001**	**<0.001**	466	-
syllabic rate²	0.56	0.83	0.67	0.815	0.503	467	0.24
PLV	0.07	0.07	1.15	0.815	0.25	182	0.03
hemisphere [r]	0.42	0.1	4.34	**<0.001**	**<0.001**	508	0.04
SP Heschl	-0.06	0.08	-0.78	0.815	0.438	312	0.003
SP SMA	0.06	0.07	0.88	0.815	0.379	328	0.0005
SMA HG	-0.02	0.07	-0.3	0.815	0.763	458	0.001
SP IFG tri	-0.06	0.08	-0.8	0.815	0.423	242	0.0004
IFG HG	0.03	0.06	0.44	0.815	0.661	426	0.0002
PLV × hemisphere [r]	0.05	0.09	0.61	0.815	0.541	513	0.0004
SP Heschl × SP SMA	0.03	0.07	0.44	0.815	0.663	308	0.006
SP Heschl × SMA HG	-0.02	0.09	-0.23	0.815	0.815	500	<.0001
SP SMA × SMA HG	-0.02	0.07	-0.29	0.815	0.769	495	0.003
hemisphere [r] × SP Heschl	0.17	0.11	1.56	0.815	0.12	357	0.008
hemisphere [r] × SP SMA	-0.05	0.11	-0.5	0.815	0.62	296	0.0006
hemisphere [r] × SMA HG	-0.06	0.11	-0.57	0.815	0.571	505	0.002
SP Heschl × SP IFG tri	0.08	0.08	1.05	0.815	0.296	329	0.002
SP Heschl × IFG HG	0.02	0.07	0.24	0.815	0.808	441	0.003
SP IFG tri × IFG HG	-0.03	0.07	-0.39	0.815	0.694	379	0.002
hemisphere [r] × SP IFG tri	0.11	0.1	1.13	0.815	0.258	289	0.0006
hemisphere [r] × IFG HG	-0.05	0.1	-0.49	0.815	0.625	469	0.0004
(SP Heschl × SP SMA) × SMA HG	0.03	0.08	0.37	0.815	0.711	496	0.0001
(hemisphere [r] × SP Heschl) × SP SMA	0.09	0.11	0.85	0.815	0.395	287	0.001
(hemisphere [r] × SP Heschl) × SMA HG	0.1	0.12	0.85	0.815	0.396	503	0.003
(hemisphere [r] × SP SMA) × SMA HG	-0.1	0.13	-0.77	0.815	0.442	478	0.002
(SP Heschl × SP IFG tri) × IFG HG	0.07	0.07	0.97	0.815	0.334	409	0.002
(hemisphere [r] × SP Heschl) × SP IFG tri	-0.09	0.11	-0.83	0.815	0.409	228	0.004
(hemisphere [r] × SP Heschl) × IFG HG	-0.16	0.11	-1.45	0.815	0.148	422	0.005
(hemisphere [r] × SP IFG tri) × IFG HG	-0.04	0.1	-0.36	0.815	0.721	424	0.0008
(hemisphere [r] × SP Heschl × SP SMA) × SMA HG	-0.05	0.11	-0.4	0.815	0.689	468	0.0002
(hemisphere [r] × SP Heschl × SP IFG tri) × IFG HG	-0.04	0.1	-0.36	0.815	0.721	429	0.0001

Random effects:

Groups	Name	Variance	Std.Dev.
sub	freq	0.0003087	0.01757
Residual		0.686327	0.82845

Number of observations: 520, groups: sub, 54

Marginal 0 R^2.315
