# Supplementary material for: Endogenous auditory and motor brain rhythms predict individual speech tracking
Source: PLoS Biol. 2026 Jul 31;24(7):e3003924. doi: 10.1371/journal.pbio.3003924 (PMC13446743; doi:10.1371/journal.pbio.3003924)
Supplement: Supplementary file 22 [file pbio.3003924.s022.pdf]

S15 Table: Statistical results of hemispheric asymmetry analyses. Results of Wilcoxon rank-sum tests comparing High vs Low synchronizers and Wilcoxon signed-rank tests assessing whether the hemispheric asymmetry index (HAI) differed from zero (Vs. Zero). Results are reported for each syllabic rate and region of interest (HG and STG), including W statistics and FDR-corrected p-values.

	Syllabic rate	Area	W	p	p FDR
High vs Low	5.0000	STG	212	0.070	0.475
High vs Low	11.0707	STG	356	0.319	0.907
High vs Low	14.0343	STG	329	0.640	0.907
High vs Low	16.0127	STG	286	0.726	0.907
High vs Low	17.5000	STG	316	0.830	0.923
High vs Low	5.0000	HG	268	0.483	0.907
High vs Low	11.0707	HG	390	0.095	0.475
High vs Low	14.0343	HG	309	0.938	0.938
High vs Low	16.0127	HG	348	0.401	0.907
High vs Low	17.5000	HG	284	0.697	0.907
Vs_Zero	5.0000	STG	1405	0.000	0.000
Vs_Zero	11.0707	STG	1309	0.000	0.000
Vs_Zero	14.0343	STG	1059	0.065	0.065
Vs_Zero	16.0127	STG	1244	0.001	0.002
Vs_Zero	17.5000	STG	1171	0.006	0.009
Vs_Zero	5.0000	HG	1288	0.000	0.001
Vs_Zero	11.0707	HG	1396	0.000	0.000
Vs_Zero	14.0343	HG	1181	0.005	0.008
Vs_Zero	16.0127	HG	1108	0.026	0.028
Vs_Zero	17.5000	HG	1139	0.013	0.017
