# Supplementary material for: Endogenous auditory and motor brain rhythms predict individual speech tracking
Source: PLoS Biol. 2026 Jul 31;24(7):e3003924. doi: 10.1371/journal.pbio.3003924 (PMC13446743; doi:10.1371/journal.pbio.3003924)
Supplement: Supplementary file 23 [file pbio.3003924.s023.pdf]

S16 Table. Predicting speech comprehension from neural audio-motor estimates involving Heschl''s Gyrus speech tracking. Syllabic rate^{2} : indicates the quadratic model of the syllabic rate effect

Predictors	*Estimates*	*std. Error*	*t*	P_{FDR}	*p*	*df*	ηp²
syllabic rate	-13.68	0.5	-27.18	**<0.001**	**<0.001**	192	-
syllabic rate^2	-6.58	0.32	-20.81	**<0.001**	**<0.001**	215	0.80
speechTracking HG	-0.1	0.05	-2.11	0.115	**0.036**	237	0.02
SP Heschlm	-0.06	0.04	-1.62	0.236	0.107	223	0.01
SP SMAm	-0.02	0.04	-0.44	0.804	0.658	226	0.0009
couplingSMA	0.05	0.02	2.05	0.115	**0.042**	236	0.02
SP IFGm	0	0.04	-0.02	0.984	0.984	224	<.0001
couplingIFG	0	0.03	0.05	0.984	0.957	241	<.0001
syllabic rate (linear) × speechTracking HG	-0.78	0.6	-1.31	0.3	0.191	232	-
Syllabic rate (quadratic) × speechTracking HG	-0.52	0.38	-1.39	0.3	0.167	211	0.01

Random effects:

Groups	Name	Variance	Std.Dev.
sub	freq	0.0009161	0.03027
Residual		0.0527843	0.22975

Number of obs: 260, groups: sub, 52

Marginal Conditional R^2: 0.938
