# Supplementary material for: Endogenous auditory and motor brain rhythms predict individual speech tracking
Source: PLoS Biol. 2026 Jul 31;24(7):e3003924. doi: 10.1371/journal.pbio.3003924 (PMC13446743; doi:10.1371/journal.pbio.3003924)
Supplement: Supplementary file 24 [file pbio.3003924.s024.pdf]

S17 Table. Predicting speech comprehension from neural audio-motor estimates + PLV involving Heschl''s Gyrus speech tracking. Syllabic rate^2 : indicates the quadratic model of the syllabic rate effect; PLV: phase-locking value, indicates strength of auditory-motor synchronization behavior

Predictors	*Estimates*	*std. Error*	*t*	*p*	*df*	ηp²
syllabic rate	-13.69	0.51	-26.85	**<0.001**	188	-
syllabic rate²	-6.57	0.33	-20.21	**<0.001**	205	0.80
speechTracking HG	-0.11	0.05	-2.18	**0.03**	227	0.02
PLV	0.02	0.06	0.27	0.788	61	0.001
SP Heschlm	-0.05	0.04	-1.18	0.239	205	0.007
SP SMAm	-0.01	0.04	-0.26	0.799	208	0.0003
couplingSMA	0.06	0.03	2.21	**0.028**	228	0.02
SP IFGm	0.02	0.04	0.38	0.707	206	0.0007
couplingIFG	0.01	0.03	0.36	0.719	232	0.0006
syllabic rate (linear) × speechTracking HG	-0.84	0.61	-1.37	0.173	222	-
syllabic rate (quadratic) × speechTracking HG	-0.4	0.41	-0.98	0.328	205	0.009
syllabic rate (linear) × PLV	0.05	0.51	0.1	0.917	185	-
syllabic rate (quadratic) × PLV	0.27	0.34	0.81	0.419	216	0.003
speechTracking HG × PLV	0.06	0.05	1.17	0.243	236	0.006
PLV × SP Heschlm	-0.06	0.04	-1.25	0.212	202	0.008
PLV × SP SMAm	0.06	0.04	1.56	0.119	208	0.01
PLV × couplingSMA	0.01	0.03	0.28	0.781	230	0.0003
PLV × SP IFGm	-0.04	0.04	-0.81	0.419	203	0.003
PLV × couplingIFG	-0.05	0.03	-1.64	0.102	232	0.01
(syllabic rate (linear) × speechTracking HG) × PLV	0.72	0.58	1.25	0.213	234	-
(syllabic rate (quadratic) × speechTracking HG) × PLV	0.11	0.35	0.32	0.75	213	0.01

Random effects:

Groups	Name	Variance	Std.Dev.
sub	freq	0.0008723	0.02953
Residual		0.0543222	0.23307

Number of obs: 260, groups: sub, 52

Marginal R^2: 0.937
