# Supplementary material for: Endogenous auditory and motor brain rhythms predict individual speech tracking
Source: PLoS Biol. 2026 Jul 31;24(7):e3003924. doi: 10.1371/journal.pbio.3003924 (PMC13446743; doi:10.1371/journal.pbio.3003924)
Supplement: Supplementary file 25 [file pbio.3003924.s025.pdf]

S18 Table. Predicting speech comprehension from neural audio-motor estimates involving STG. Syllabic rate^2 : indicates the quadratic model of the syllabic rate effect

Predictors	*Estimates*	*std. Error*	*t*	*p*	*p*	*df*	ηp²
syllabic rate	-12.8	0.52	-24.48	**<0.001**	**<0.001**	181	-
syllabic rate^2	-6.26	0.32	-19.79	**<0.001**	**<0.001**	198	0.78
speechTracking STG	-0.06	0.06	-1.04	0.504	0.301	217	0.005
SP STGm	0.01	0.04	0.14	0.891	0.891	204	<.0001
SP SMAm	-0.04	0.04	-1	0.504	0.32	210	0.005
couplingSMA	0.06	0.02	2.23	0.097	**0.027**	214	0.02
SP IFGm	-0.01	0.04	-0.36	0.79	0.718	208	<.0001
couplingIFG	-0.02	0.02	-0.84	0.55	0.4	215	0.003
syllabic rate (linear) × speechTracking STG	-0.84	0.7	-1.19	0.504	0.235	215	-
syllabic rate (quadratic) × speechTracking STG	-0.64	0.45	-1.42	0.434	0.158	196	0.01

Random effects:

Groups	Name	Variance	Std.Dev.
sub	freq	0.0009633	0.03104
Residual		0.0527531	0.22968

Number of observations: 240, groups: sub, 48

Marginal R^2: 0.937
