# Supplementary material for: Endogenous auditory and motor brain rhythms predict individual speech tracking
Source: PLoS Biol. 2026 Jul 31;24(7):e3003924. doi: 10.1371/journal.pbio.3003924 (PMC13446743; doi:10.1371/journal.pbio.3003924)
Supplement: Supplementary file 26 [file pbio.3003924.s026.pdf]

S19 Table. Predicting speech comprehension from neural audio-motor estimates + PLV involving STG. Syllabic rate^2: indicates the quadratic model of the syllabic rate effect; PLV: phase-locking value, indicates strength of auditory-motor synchronization behavior

Predictors	*Estimates*	*std. Error*	*t*	*p*	*df*	ηp²
syllabic rate	-12.79	0.54	-23.78	**<0.001**	**163**	**-**
syllabic rate²	-6.19	0.32	-19.18	**<0.001**	**186**	**0.79**
speechTracking STG	-0.06	0.06	-0.9	0.37	206	0.004
PLV	0.03	0.07	0.51	0.608	51	0.005
SP STGm	0	0.04	0	0.999	173	<.0001
SP SMAm	-0.03	0.04	-0.67	0.504	192	0.002
couplingSMA	0.06	0.03	2.21	**0.028**	**204**	**0.02**
SP IFGm	-0.01	0.04	-0.14	0.89	195	0.0001
couplingIFG	-0.02	0.03	-0.75	0.456	205	0.003
syllabic rate (linear) × speechTracking STG	-0.58	0.72	-0.8	0.422	205	-
syllabic rate (quadratic) × speechTracking STG	-0.51	0.47	-1.09	0.277	189	0.006
freq × PLV	0.32	0.55	0.58	0.56	166	-
freq × PLV	0.06	0.33	0.18	0.859	187	0.002
speechTracking STG × PLV	0.08	0.06	1.33	0.184	207	0.009
PLV × SP STGm	0.05	0.05	1.09	0.277	171	0.007
PLV × SP SMAm	0.02	0.04	0.55	0.584	199	0.002
PLV × couplingSMA	0.02	0.03	0.78	0.436	197	0.003
PLV × SP IFGm	-0.01	0.04	-0.27	0.785	195	0.0004
PLV × couplingIFG	-0.02	0.03	-0.63	0.532	207	0.002
(syllabic rate (linear)× speechTracking STG) × PLV	0.62	0.71	0.88	0.381	206	-
(syllabic rate (quadratic) × speechTracking STG) × PLV	0.35	0.48	0.73	0.464	190	0.004

Random effects:

Groups	Name	Variance	Std.Dev.
sub	freq	0.001018	0.03191
Residual		0.053514	0.23133

Number of obs: 240, groups: sub, 48

Marginal R^2: 0.936
