# Supplementary material for: Endogenous auditory and motor brain rhythms predict individual speech tracking
Source: PLoS Biol. 2026 Jul 31;24(7):e3003924. doi: 10.1371/journal.pbio.3003924 (PMC13446743; doi:10.1371/journal.pbio.3003924)
Supplement: Supplementary file 27 [file pbio.3003924.s027.pdf]

S20 Table. Spearman rank correlations between spectral peak measures and behavioral frequency variables. Correlations were computed between theta frequency of speech-production–related regions (inferior frontal gyrus, IFG; supplementary motor area, SMA) and the preferred motor rate, and between theta frequency of auditory regions (superior temporal gyrus, STG; Heschl's gyrus, HG) and the preferred auditory rate. Analyses were conducted both separately for left and right hemispheres and for bilateral averages (mean of left and right homologous regions). Correlation coefficients (Spearman's ρ) and corresponding two-tailed p-values are reported. P-values were additionally corrected for multiple comparisons using the false discovery rate (FDR) procedure. All correlations were computed using pairwise complete observations.

neural	behavioral	type	rho	p	p_{FDR}
SP.IFG_tri_l	articulation_freq	separate_LR	0.12615056	0.3542169	0.8916044
SP.IFG_tri_r	articulation_freq	separate_LR	0.01432261	0.9165595	0.9165595
SP.SMA.l	articulation_freq	separate_LR	0.050005	0.7118321	0.8916044
SP.SMA.r	articulation_freq	separate_LR	-0.0448031	0.7430036	0.8916044
SP.STG.r	preferred_audio_peak	separate_LR	-0.0959658	0.4858242	0.8916044
SP.STG.l	preferred_audio_peak	separate_LR	0.02490392	0.8554346	0.9165595
SP.Heschl.l	preferred_audio_peak	separate_LR	-0.1279826	0.3517601	0.8916044
SP.Heschl.r	preferred_audio_peak	separate_LR	-0.0635989	0.6414531	0.8916044
SP.IFGm	articulation_freq	bilateral_mean	0.08221992	0.5431707	0.8916044
SP.SMAm	articulation_freq	bilateral_mean	0.04984275	0.7127266	0.8916044
SP.STGm	preferred_audio_peak	bilateral_mean	-0.0897575	0.5067012	0.8916044
SP.HGm	preferred_audio_peak	bilateral_mean	-0.1220381	0.3658144	0.8916044
