# Supplementary material for: Endogenous auditory and motor brain rhythms predict individual speech tracking
Source: PLoS Biol. 2026 Jul 31;24(7):e3003924. doi: 10.1371/journal.pbio.3003924 (PMC13446743; doi:10.1371/journal.pbio.3003924)
Supplement: Supplementary file 28 [file pbio.3003924.s028.pdf]

S21 Table. Spearman rank correlations between spectral peak measures in various ROIs and speech tracking in Superior Temporal Gyrus (STG). Peak frequencies of the theta cluster of endogenous brain rhythms with maximum amplitude were selected for each participant. Peak frequencies were averaged across the left and right hemisphere. Spearman correlations between the peak frequencies in each region of interest (ROI, AAL labels are displayed) and the speech tracking in STG (averaged across hemispheres and syllabic rate conditions) were computed. Correlation coefficients (Spearman's ρ, *rho*) and corresponding two-tailed p-values are reported. P-values are additionally reported corrected for multiple comparisons using the false discovery rate (FDR) procedure (p_{FDR}). Significance is indicated by asterix (* < .05; ** < .01).

ROI	**rho**	**p**	**p_{FDR}**
Frontal_Inf_Tri_	-0.0079033	0.95347203	0.95347203
Supp_Motor_Area_	0.31069408	0.01865964 *	0.1399473
Heschl_	-0.1459404	0.27870744	0.61447408
Temporal_Sup_	-0.3857494	0.00304215 **	0.04563231 *
Parietal_Inf_	-0.0872407	0.51873752	0.78573122
Precentral_	-0.1520786	0.25876514	0.61447408
Insula_	0.06824721	0.6139602	0.78573122
Rolandic_Oper_	-0.0697992	0.60590505	0.78573122
Supra_Marginal_Gyrus_	0.03996368	0.76787728	0.82272566
Frontal_Inf_Oper_	0.06545149	0.62858498	0.78573122
Frontal_Sup_Medial_	0.20077209	0.13426087	0.50347825
Frontal_Mid_	0.14354612	0.28675457	0.61447408
Frontal_Sup_	-0.1094411	0.41771332	0.78321248
Frontal_Inf_Orb_	0.28079285	0.03436671 *	0.17183354
Cingulum_Mid_	-0.0412608	0.7605635	0.82272566
