# Supplementary material for: Endogenous auditory and motor brain rhythms predict individual speech tracking
Source: PLoS Biol. 2026 Jul 31;24(7):e3003924. doi: 10.1371/journal.pbio.3003924 (PMC13446743; doi:10.1371/journal.pbio.3003924)
Supplement: Supplementary file 30 [file pbio.3003924.s030.pdf]

S23 Table. Spearman rank correlations between spectral peak measures in various regions of interest (ROIs) and speech comprehension performance. Peak frequencies of the theta cluster of endogenous brain rhythms with maximum amplitude were selected for each participant. Peak frequencies were averaged across the left and right hemisphere. Spearman correlations between the peak frequencies in each region of interest (ROI, AAL labels are displayed) and the performance in the speech intelligibility task (averaged across syllabic rate conditions) were computed. Correlation coefficients (Spearman's ρ, *rho*) and corresponding two-tailed p-values are reported. P-values are additionally reported corrected for multiple comparisons using the false discovery rate (FDR) procedure (p_{FDR}). Significance is indicated by asterix (* < .05; ** < .01).

ROI	**rho**	**p**	**p_{FDR}**
Frontal_Inf_Tri_	-0.0655353	-0.0655353 *	0.9483241
Supp_Motor_Area_	-0.2583413	-0.2583413	0.4003309
Heschl_	-0.1917058	-0.1917058	0.73163583
Temporal_Sup_	0.02165519	0.02165519 *	0.4003309
Parietal_Inf_	-0.0600818	-0.0600818	0.55695143
Precentral_	-0.0937698	-0.0937698	0.4003309
Insula_	0.06619882	0.06619882	0.9483241
Rolandic_Oper_	-0.2041887	-0.2041887	0.83152586
Supra_Marginal_Gyrus_	-0.1104629	-0.1104629	0.94022409
Frontal_Inf_Oper_	0.03500062	0.03500062	0.68387159
Frontal_Sup_Medial_	-0.2734312	-0.2734312	0.4003309
Frontal_Mid_	-0.173316	-0.173316	0.54570134
Frontal_Sup_	-0.272233	-0.272233	0.55695143
Frontal_Inf_Orb_	-0.0638269	-0.0638269	0.54570134
Cingulum_Mid_	-0.257469	-0.257469	0.9079334
