# Supplementary material for: Endogenous auditory and motor brain rhythms predict individual speech tracking
Source: PLoS Biol. 2026 Jul 31;24(7):e3003924. doi: 10.1371/journal.pbio.3003924 (PMC13446743; doi:10.1371/journal.pbio.3003924)
Supplement: Supplementary file 31 [file pbio.3003924.s031.pdf]

S24 Table. Spearman rank correlations between spectral peak measures in various ROIs and speech tracking in the Superior Temporal Gyrus (STG) separately computed for the left and right hemisphere. Peak frequencies of the theta cluster of endogenous brain rhythms with maximum amplitude were selected for each participant. Spearman correlations between the peak frequencies of the left and right hemisphere in each region of interest (ROI, AAL labels are displayed) and the left hemispheric speech tracking in STG, as well as the right hemispheric tracking in STG were computed separately. Correlation coefficients (Spearman's ρ, *rho*) and corresponding two-tailed p-values are reported. P-values are additionally reported corrected for multiple comparisons, within the left and right hemispheric tracking analyses separately, using the false discovery rate (FDR) procedure (p_{FDR}). Significance is indicated by asterix (* < .05; ** < .01).

**Correlation with left hemispheric speech tracking in STG**			
ROI	**rho**	**p**	**p_{FDR}**
Frontal_Inf_Tri_L	0.00698148	0.95927222	0.95927222
Frontal_Inf_Tri_R	-0.1260666	0.35453984	0.66357996
Supp_Motor_Area_L	0.16734216	0.21342197	0.49137023
Supp_Motor_Area_R	0.40326254	0.0020583 **	0.06174898
Heschl_L	-0.2514404	0.06405822	0.3843493
Heschl_R	-0.1115682	0.41299793	0.66357996
Temporal_Sup_L	-0.3642595	0.00578185 **	0.07360101
Temporal_Sup_R	-0.0910065	0.50874464	0.66357996
Parietal_Inf_L	-0.1012854	0.45344871	0.66357996
Parietal_Inf_R	0.12620809	0.34953683	0.66357996
Precentral_L	0.01324304	0.92211339	0.95927222
Precentral_R	-0.1809831	0.18192208	0.49137023
Supra_Marginal_Gyrus_L	-0.0297702	0.82600436	0.95927222
Supra_Marginal_Gyrus_R	-0.0268087	0.84450974	0.95927222
Insula_L	0.09846806	0.46617222	0.66357996
Insula_R	0.00791754	0.95381716	0.95927222
Rolandic_Oper_L	-0.232751	0.0968173	0.48408651
Rolandic_Oper_R	-0.0226839	0.86942805	0.95927222
Frontal_Inf_Oper_L	0.10537096	0.43534152	0.66357996
Frontal_Inf_Oper_R	0.1945623	0.15074512	0.49137023
Frontal_Sup_Medial_L	0.16176097	0.22930611	0.49137023
Frontal_Sup_Medial_R	0.09217205	0.50740217	0.66357996
Frontal_Mid_L	0.20819304	0.12361855	0.49137023
Frontal_Mid_R	0.17623201	0.18974432	0.49137023
Frontal_Sup_L	0.16176097	0.22930611	0.49137023
Frontal_Sup_R	0.09217205	0.50740217	0.66357996
Frontal_Inf_Orb_L	0.19223407	0.15197742	0.49137023
Frontal_Inf_Orb_R	0.35137482	0.0073601 **	0.07360101
Cingulum_Mid_L	0.26416597	0.06104359	0.3843493
Cingulum_Mid_R	-0.0122576	0.92789325	0.95927222

**Correlation with right hemispheric speech tracking in STG**			
ROI	**rho**	**p**	**p_{FDR}**
Frontal_Inf_Tri_L	0.0319351	0.81525678	0.9406809
Frontal_Inf_Tri_R	-0.0369136	0.78708513	0.9406809
Supp_Motor_Area_L	0.15528562	0.24873805	0.74621414
Supp_Motor_Area_R	0.26819238	0.0456648 *	0.45664803
Heschl_L	-0.0471519	0.73246643	0.9406809
Heschl_R	-0.0354422	0.79538504	0.9406809
Temporal_Sup_L	-0.2697297	0.04439128 *	0.45664803
Temporal_Sup_R	-0.3437416	0.01018386 *	0.30551588
Parietal_Inf_L	-0.1419565	0.2921806	0.79685618
Parietal_Inf_R	0.01089801	0.93587325	0.94708416

Precentral_L	0.08419026	0.53351618	0.86716299
Precentral_R	-0.1758398	0.19486768	0.73075379
Supra_Marginal_Gyrus_L	0.12645788	0.3485762	0.84305182
Supra_Marginal_Gyrus_R	0.07592361	0.57810866	0.86716299
Insula_L	-0.2031617	0.12959231	0.64796154
Insula_R	0.03816392	0.7800507	0.9406809
Rolandic_Oper_L	-0.1003725	0.47894977	0.86716299
Rolandic_Oper_R	0.16758199	0.2213502	0.73783399
Frontal_Inf_Oper_L	-0.0792486	0.557888	0.86716299
Frontal_Inf_Oper_R	0.11644076	0.39276033	0.84305182
Frontal_Sup_Medial_L	0.22327017	0.09502716	0.57016297
Frontal_Sup_Medial_R	-0.0203457	0.88390068	0.94703645
Frontal_Mid_L	0.08787683	0.51955875	0.86716299
Frontal_Mid_R	0.00898976	0.94708416	0.94708416
Frontal_Sup_L	0.22327017	0.09502716	0.57016297
Frontal_Sup_R	-0.0203457	0.88390068	0.94703645
Frontal_Inf_Orb_L	0.08470824	0.53099219	0.86716299
Frontal_Inf_Orb_R	0.18586387	0.16628238	0.71263878
Cingulum_Mid_L	-0.1220819	0.39342418	0.84305182
Cingulum_Mid_R	0.04883099	0.71831358	0.9406809
