# Supplementary material for: Endogenous auditory and motor brain rhythms predict individual speech tracking
Source: PLoS Biol. 2026 Jul 31;24(7):e3003924. doi: 10.1371/journal.pbio.3003924 (PMC13446743; doi:10.1371/journal.pbio.3003924)
Supplement: Supplementary file 32 [file pbio.3003924.s032.pdf]

S25 Table. Spearman rank correlations between spectral peak measures in various ROIs and speech tracking in the Heschl's Gyrus (HG) separately computed for the left and right hemisphere. Peak frequencies of the theta cluster of endogenous brain rhythms with maximum amplitude were selected for each participant. Spearman correlations between the peak frequencies of the left and right hemisphere in each region of interest (ROI, AAL labels are displayed) and the left hemispheric speech tracking in HG, as well as the right hemispheric tracking in HG were computed separately. Correlation coefficients (Spearman's ρ, *rho*) and corresponding two-tailed p-values are reported. P-values are additionally reported corrected for multiple comparisons, within the left and right hemispheric tracking analyses separately, using the false discovery rate (FDR) procedure (p_{FDR}). Significance is indicated by asterix (* < .05; ** < .01).

**Correlation with left hemispheric speech tracking in HG**			
ROI	**rho**	**p**	**p_{FDR}**
Frontal_Inf_Tri_L	-0.0392276	0.77407988	0.86008876
Frontal_Inf_Tri_R	-0.2127288	0.11546709	0.39808347
Supp_Motor_Area_L	0.18447167	0.16953568	0.39808347
Supp_Motor_Area_R	0.27017512	0.04402778 *	0.37597213
Heschl_L	-0.2373718	0.08098528	0.37597213
Heschl_R	-0.0718216	0.59887333	0.74859167
Temporal_Sup_L	-0.2361345	0.07975932	0.37597213
Temporal_Sup_R	-0.0892887	0.51681017	0.70474115
Parietal_Inf_L	-0.0199432	0.88293717	0.91338328
Parietal_Inf_R	0.07566612	0.57587956	0.74859167
Precentral_L	-0.0227071	0.86685235	0.91338328
Precentral_R	-0.1925124	0.155179	0.39808347
Supra_Marginal_Gyrus_L	0.00075078	0.99557759	0.99557759
Supra_Marginal_Gyrus_R	-0.1199174	0.37868479	0.59792336
Insula_L	0.15808345	0.24020893	0.45039175
Insula_R	-0.0912948	0.50338188	0.70474115
Rolandic_Oper_L	-0.2463372	0.07833643	0.37597213
Rolandic_Oper_R	-0.0662559	0.6307941	0.75695292
Frontal_Inf_Oper_L	0.11379541	0.39931492	0.59897239
Frontal_Inf_Oper_R	0.18794007	0.16541447	0.39808347
Frontal_Sup_Medial_L	0.1726522	0.19904173	0.39808347
Frontal_Sup_Medial_R	0.14026182	0.31172745	0.51954575
Frontal_Mid_L	0.23029194	0.08772683	0.37597213
Frontal_Mid_R	0.18195277	0.17553938	0.39808347
Frontal_Sup_L	0.1726522	0.19904173	0.39808347
Frontal_Sup_R	0.14026182	0.31172745	0.51954575
Frontal_Inf_Orb_L	0.18830344	0.16069207	0.39808347
Frontal_Inf_Orb_R	0.2358449	0.07737083	0.37597213
Cingulum_Mid_L	0.28872029	0.03990378 *	0.37597213
Cingulum_Mid_R	-0.0577999	0.66932865	0.77230229

**Correlation with right hemispheric speech tracking in HG**			
ROI	**rho**	**p**	**p_{FDR}**
Frontal_Inf_Tri_L	-0.0062902	0.96330176	0.96961844
Frontal_Inf_Tri_R	0.01792056	0.89570334	0.96961844
Supp_Motor_Area_L	0.16441038	0.22166678	0.74786348
Supp_Motor_Area_R	0.18244734	0.17835267	0.74786348
Heschl_L	-0.0123833	0.92850074	0.96961844
Heschl_R	0.00520698	0.96961844	0.96961844
Temporal_Sup_L	-0.1199336	0.37861967	0.94654918
Temporal_Sup_R	-0.2235691	0.10084066	0.74786348
Parietal_Inf_L	-0.1811891	0.1773896	0.74786348
Parietal_Inf_R	-0.0119748	0.92955269	0.96961844

Precentral_L	0.00653937	0.96149481	0.96961844
Precentral_R	-0.2306559	0.08721292	0.74786348
Supra_Marginal_Gyrus_L	0.08882083	0.51116386	0.96961844
Supra_Marginal_Gyrus_R	-0.0186973	0.89120922	0.96961844
Insula_L	-0.1975554	0.14074354	0.74786348
Insula_R	-0.0472621	0.72942247	0.96961844
Rolandic_Oper_L	-0.1336849	0.34474506	0.94021379
Rolandic_Oper_R	0.03572981	0.79565393	0.96961844
Frontal_Inf_Oper_L	-0.1409953	0.29549415	0.88648245
Frontal_Inf_Oper_R	0.16496924	0.22435905	0.74786348
Frontal_Sup_Medial_L	0.19587807	0.14421543	0.74786348
Frontal_Sup_Medial_R	-0.0242761	0.8616741	0.96961844
Frontal_Mid_L	0.06954754	0.61052427	0.96961844
Frontal_Mid_R	0.05024459	0.71051177	0.96961844
Frontal_Sup_L	0.19587807	0.14421543	0.74786348
Frontal_Sup_R	-0.0242761	0.8616741	0.96961844
Frontal_Inf_Orb_L	0.04200436	0.75638065	0.96961844
Frontal_Inf_Orb_R	0.08213276	0.54359977	0.96961844
Cingulum_Mid_L	-0.0957343	0.50396262	0.96961844
Cingulum_Mid_R	-0.0164099	0.90356816	0.96961844
