# Supplementary material for: Endogenous auditory and motor brain rhythms predict individual speech tracking
Source: PLoS Biol. 2026 Jul 31;24(7):e3003924. doi: 10.1371/journal.pbio.3003924 (PMC13446743; doi:10.1371/journal.pbio.3003924)
Supplement: Supplementary file 33 [file pbio.3003924.s033.pdf]

S26 Table. Spearman rank correlations between spectral peak measures in various ROIs of the left and right hemisphere and speech comprehension performance. Peak frequencies of the theta cluster of endogenous brain rhythms with maximum amplitude were selected for each participant. Spearman correlations between the peak frequencies of the left and right hemisphere in each region of interest (ROI, AAL labels are displayed) and the performance in the intelligibility task were computed. Correlation coefficients (Spearman's ρ, *rho*) and corresponding two-tailed p-values are reported. P-values are additionally reported corrected for multiple comparisons using the false discovery rate (FDR) procedure (p_{FDR}). Significance is indicated by asterix (* < .05; ** < .01).

	**Correlation with performance**		
ROI	**rho**	**p**	**p_{FDR}**
Frontal_Inf_Tri_L	-0.160505	0.23732515	0.60931868
Frontal_Inf_Tri_R	-0.0046704	0.97274791	0.97274791
Supp_Motor_Area_L	-0.1462926	0.27753628	0.60931868
Supp_Motor_Area_R	-0.1699247	0.21055201	0.60931868
Heschl_L	-0.1513845	0.26990401	0.60931868
Heschl_R	-0.0977523	0.47353958	0.6244503
Temporal_Sup_L	0.13196155	0.33230301	0.62306815
Temporal_Sup_R	-0.102227	0.45768072	0.6244503
Parietal_Inf_L	-0.0895811	0.5075402	0.63409841
Parietal_Inf_R	-0.040786	0.76323863	0.81014207
Precentral_L	-0.0957245	0.47874523	0.6244503
Precentral_R	-0.0444259	0.74509356	0.81014207
Supra_Marginal_Gyrus_L	-0.1383725	0.30465934	0.60931868
Supra_Marginal_Gyrus_R	0.2106545	0.1191424	0.60931868
Insula_L	-0.1420795	0.29175837	0.60931868
Insula_R	-0.1550865	0.25374227	0.60931868
Rolandic_Oper_L	-0.0391039	0.78313733	0.81014207
Rolandic_Oper_R	-0.0518907	0.70673393	0.81014207
Frontal_Inf_Oper_L	-0.1199995	0.37393679	0.6244503
Frontal_Inf_Oper_R	0.15769169	0.24575553	0.60931868
Frontal_Sup_Medial_L	-0.1014656	0.45264177	0.6244503
Frontal_Sup_Medial_R	-0.2885001	0.0343754 *	0.51563098
Frontal_Mid_L	-0.1067595	0.43354634	0.6244503
Frontal_Mid_R	-0.2167023	0.10542058	0.60931868
Frontal_Sup_L	-0.1014656	0.45264177	0.6244503
Frontal_Sup_R	-0.2885001	0.0343754 *	0.51563098
Frontal_Inf_Orb_L	0.16072247	0.23234967	0.60931868
Frontal_Inf_Orb_R	-0.1798989	0.18054759	0.60931868
Cingulum_Mid_L	-0.2510151	0.07561213	0.60931868
Cingulum_Mid_R	-0.0852383	0.52841534	0.63409841
