# Supplementary material for: Endogenous auditory and motor brain rhythms predict individual speech tracking
Source: PLoS Biol. 2026 Jul 31;24(7):e3003924. doi: 10.1371/journal.pbio.3003924 (PMC13446743; doi:10.1371/journal.pbio.3003924)
Supplement: Supplementary file 34 [file pbio.3003924.s034.pdf]

We want to thank the Editor and the Reviewers for their time and effort, their positive evaluation and constructive feedback. We thoroughly revised the manuscript (PBIOLOGY-D-25-00976_R1), implementing the reviewers' suggestions. In summary, we did the following revisions:

Following Reviewer 1, we clarified the theoretical background and evidence for motor areas aiding speech comprehension. We clarified the exclusion of participants from the group analysis who could not unambiguously be classified as high or low synchronizer. Note that these participants were not excluded from the general analyses, where no group affiliation was required (see also comment of Reviewer 2). We revised our figures to show the LMM predictions overlayed for high and low auditory-motor synchronizers.

We also revised the analysis of the *neural measures – speech comprehension relationship* to provide a more complete picture, as requested by Reviewer 1 and Reviewer 3. Although the novel analysis shows no significant effects, an exploratory analysis without control for multiple comparisons suggests a relationship between the neural tracking in Heschl's Gyrus and the performance in the speech intelligibility task. Furthermore, the exploratory analysis reveals a relationship between the SMA-Heschl's Gyrus/SMA-STG auditory-motor coupling and the performance in the speech intelligibility task. We now added a summary figure 7 that illustrated our main findings and also includes the exploratory findings about the *neural measures – speech comprehension relationship*.

Following Reviewer 2, we now discuss the choice of motor ROI considering the previous literature. We have added two novel analyses to test for hemispheric asymmetries. First for comparability with the previous literature, we analyze the hemispheric asymmetry index in HG and STG speech tracking in high versus low auditory-motor synchronizers. We find significant right hemispheric asymmetry, with no differences across synchronizer groups. Second, we conducted our neural LMM analyses with data that were not averaged across hemispheres and with the additional predictors' hemisphere and its interactions with the endogenous auditory and motor brain rhythms and the auditory-motor coupling. As expected, we did find a right hemispheric lateralization of speech tracking in HG and STG (main effect). However, we found no interactions. These new results are stated in the results section and discussion. The figures and tables have been added to the supplement (Supplementary figures 4 and 5, Tables 11-1411).

Furthermore, we revised our figures to overlay the LMM predictions with single data points of the raw data (as requested by Reviewer 2). We clarified the nature of the speech repetition task and more cautiously refer to the behavioral performance in the intelligibility task as estimate of speech comprehension. Referring to Reviewer 3 (And Reviewer 2), we clarified all methodological concerns, including the choice of the Gaussian-Copula Mutual Information method, the misunderstandings about the DPSS analyses and the choices for the spectral profile analysis method. Comprehensive figures of the spectral profiles of the ROIs (beyond the theta peaks) are provided in the supplement (as requested by Reviewer 3).

PBIOLOGY-D-25-00976_R1

Reviewer 1

R1 issue 1

Main This study shows that the resting-state frequency of endogenous superior temporal gyrus rhythms predicts speech tracking, and that high synchronizers show an additional association related to motor areas. The interpretation is that high synchronizers recruit motor areas for speech comprehension, while the default pathway in low synchronizers relies more on working memory. The study has above-average statistical power, the methods are clearly articulated, the writing style is matter-of-fact, and the visualizations are elegant. I have a number of specific larger and minor suggestions for improvement. My more general critique is that I believe the study has limitations and assumptions that hinder much of the field at large—namely, it targets mechanistic hypotheses through a chain of correlational patterns that are assumed to be causally linked. And while the resting state associations are consistent with the notion that oscillations are actively involved, it constitutes a weak confirmation. The tricky but increasingly tractable question that remains is whether oscillations are actively and causally involved during speech processing. Notwithstanding that, the results are clear, sound, and streamlined.

We want to thank the reviewer for the positive evaluation and helpful feedback. First, we want to clarify that we are not claiming to provide causal evidence. However, we believe that our correlational analysis paves the way for investigating a causal link. We are establishing systematics between individual differences in endogenous resting state rhythms and task-related neural activity and behaviour. Given that the motor system recruitment is assumed to be trainable, these systematics can be tested in a causal manner in the future, e.g. resting state rhythms/task related activity/behaviour can be measured prior to and after training. Furthermore, effects of long-term experience on the plasticity of brain rhythms and the impact on behavior can be assessed (note our initial research in this field, Lubinus et al., 2021, Cerebral Cortex). Importantly, the systematics we describe have been understudied even though they have been claimed to be crucial (for Review: Atanasova, Gross, Rimmele, Keitel, 2026, Neuroscience & Biobehavioral Reviews). Thus, this correlational work is important as an initial step to establish causal links. Furthermore, as the reviewer points out, establishing causality is in general a challenge. The methods that are available to test "causal" relationships like tACS, come with their own discussions about their effectiveness.

We agree with the reviewer that the link between speech comprehension and the neural correlates is crucial and therefore now advanced our analysis of the behavior-neural relationship. For details, see our answers below.

R1 issue 2

The presentation of neural results could be enriched. While the preprocessing and spectral fingerprinting is clearly documented, I was left wondering how other neural sources predict speech comprehension. What could be highly instructive is a more extensive plot that shows the directionality and strength between all MEG sensors (or sources) and behaviour. More specifically, the reader is shown main and interaction effects in SMA, STG, and STG-IFG. But beyond that, how do the main and interaction effects spread across regions? Source or sensor-wise visualization with testing-corrected main and interaction patterns could be shown, separately per low and high synchronizers or jointly. This achieves two things:

(1) It informs researchers of the scope of brain-to-behaviour correlations across brain region they are interested in, allowing them to make their own theoretical inferences and hypotheses.
(2) It allows us to judge whether the key inferences that the Authors draw from the neural results are the most parsimonious and explanatory.

As we understand, the reviewer asks for mixed models on the behavior-brain relationship performed separately per ROI brain wide. The reviewer also mentions that he/she would like to see the main effect and interactions (in SMA, STG, and STG-IFG) stemming from a different mixed model analysis), also brain wide. However, our analysis is already computationally heavy and takes several days at the moment. Performing the mixed model analysis 116 times (that is the number of ROIs in the AAL atlas) would be out of scope for this study.

In order to enrich the presentation of neural results, we now revised the analysis of the neural tracking- and behavior relationship to provide a more extensive picture. This has been revised in the manuscript. Furthermore, we added a summary figure 7.

> l. 357: "**Relationship of neural auditory-motor parameters and comprehension**
> For both behavioral and neural analyses the effects exhibited similar patterns (Figures 2B and 6), with effects related to endogenous motor brain rhythms observed predominantly in individuals displaying stronger behavioral auditory-motor synchronization (high synchronizers). To directly test the relationship between the neural estimates and speech comprehension, LMM analyses were conducted separately for STG (N=48) and HG (N=52), with performance in the speech intelligibility task predicted by the syllabic rate, the neural tracking in auditory brain areas (HG, STG), the frequencies of auditory (HG, STG) and speech motor areas (IFG, SMA), as well as the auditory-motor coupling. Additionally, we performed LMM analyses with the additional predictor auditory-motor synchronization (PLV) and its interaction with the other parameters. The only significant effects that were observed were effects of the syllabic rate, with higher syllabic rates related to lower comprehension (HG: linear: b = -13.68, SE = 0.5, statistic = -27.18, p_FDR <0.001; quadratic: b = -6.58., SE = 0.32, statistic = -20.81, p_FDR <0.001; STG: linear: b = -12.8, SE = 0.52, statistic = -24.48, p_FDR <0.001; quadratic: b = -6.26, SE = 0.32, statistic = -19.79, p_FDR <0.001; Supplementary Tables 16, 18; for models with PLV included similar effects were observed, Supplementary Tables 17, 19).

Additionally, to further explore the relationship between the neural estimates and comprehension performance, the analyses were inspected as exploratory analyses without FDR correction. For the HG model findings revealed a positive effect of SMA coupling (b = 0.05, SE = 0.02, statistic = 2.05, p = 0.042) and a negative effect of HG speech tracking, with higher coupling and lower tracking related to higher comprehension (b = -0.1, SE = 0.05, statistic = - 2.11, p = 0.036, Supplementary Table 16; similar results were observed for the model with auditory-motor synchronization (PLV) predictors included, Supplementary Table 17). For the STG model, a positive effect of SMA coupling was observed (b = 0.06, SE = 0.02, statistic = 0.1, FDR = 0.027, Supplementary Table 18). We found no interactions with auditory-motor synchronization (PLV, Supplementary Table 19).**“**

l. 408 ff.: “The behavioral findings paralleled the neural results by showing similar patterns of results for high and low synchronizers. Although we found no evidence for a significant correlation between neural estimates and comprehension performance, an exploratory analysis suggests such a relationship may be present, albeit weak."

l. 444: “Previous studies (36) reported that comprehension was predicted by neural tracking. Others, however, did not report a correlation, pointing towards a more complex link between comprehension and tracking (67,69–71). Although comprehension and neural tracking both declined at higher rates, we find no significant correlation between them. An exploratory analysis (without multiple comparison control) reveals reduced neural tracking in Heschl’s Gyrus was related to higher speech comprehension. However, these findings need to be interpreted cautiously. Thus overall, the relationship between neural tracking and comprehension seems to be complex, likely due to both measures being affected by various factors differently (72)."

l. 547: “We found no direct relationship between the auditory-motor neural estimates and speech comprehension. An exploratory analysis (without control for multiple comparisons) however, suggests that lower speech tracking in Heschl’s Gyrus was related to higher performance in the intelligibility task. Furthermore, overall higher auditory-motor coupling between SMA and Heschl’s Gyrus and SMA and STG was related to higher comprehension. "

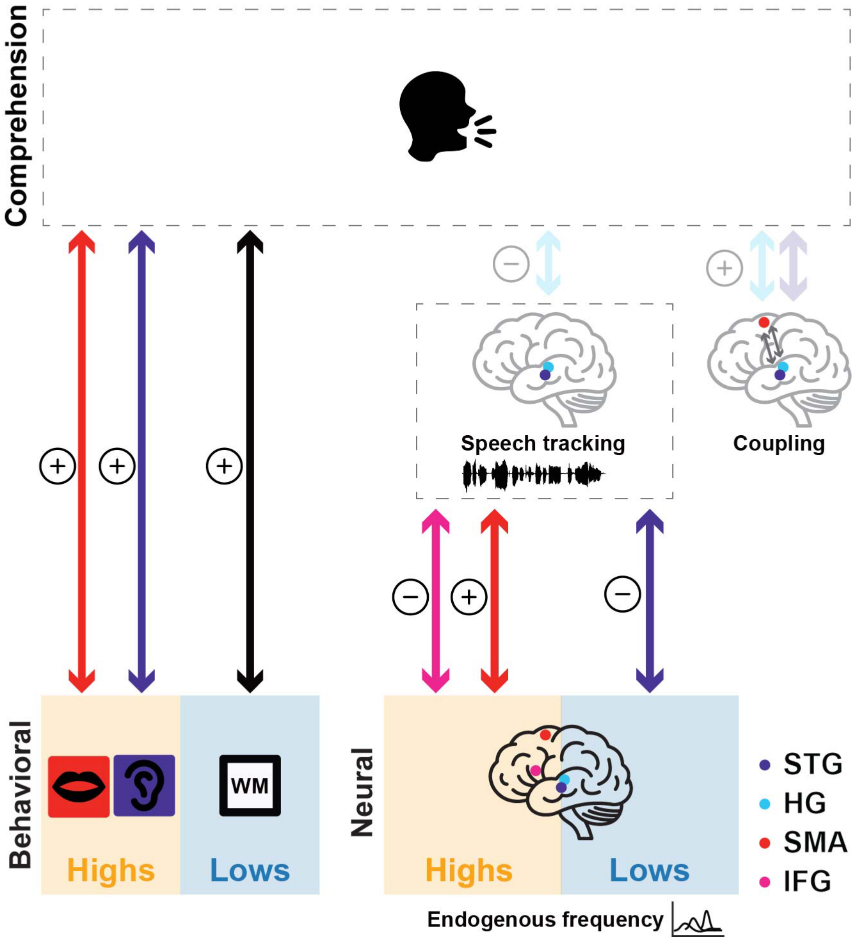

Figure 7 Schematic summary of the results. The left part of the figure illustrates the effects of the behavioral auditory-motor parameters on speech comprehension (black speaker icon). Individuals with high auditory-motor synchronization (Highs) show effects of the spontaneous speech production rate (red arrow, red mouth icon) on speech comprehension and effects of the preferred auditory rate (blue arrow, ear icon). In contrast, low auditory-motor synchronizers (Lows) show effects of working memory (black arrow, WM icon). The direction of the effects is indicated (+/-). The right part of the figure illustrates the effects of the neural auditory-motor parameters on speech comprehension. In Highs, speech tracking in HG was predicted by the endogenous frequency of IFG (pink arrow, pink region of interest, ROI), with lower rates related to higher tracking. Speech tracking in STG was predicted by the endogenous frequency of SMA (red arrow/ROI), with higher frequencies related to higher tracking. In the Lows, higher tracking in STG was predicted by lower endogenous frequencies of STG (blue arrow/ROI). The behavioral results parallel the neural results with effects of the frequencies of the endogenous motor rhythms only observed in high auditory-motor synchronizers. In an exploratory analysis (with no control for multiple comparisons), higher speech comprehension was predicted by lower tracking in HG (light blue arrow) and higher auditory-motor coupling between Heschl's Gyrus and SMA and STG and SMA. The "mouth" icon by little_dipper_studio, the "Brain" icon by Lewen Design were used under the Creative Commons Attribution 3.0 license via The Noun Project. The "Brain" icon by Lewen Design was used under the Creative Commons Attribution 3.0 license via The Noun Project.

R1 issue 3

Methods clarification

Second, I have concerns about the removal of 10 participants with synchronization abilities between 45 and 55%. First, were these participants removed from only the Group model or only the General model? I do not believe it is correct to remove them from the General model, because its premise is to leverage the full variance of the data, and removal of the centre of this distribution could cause type-1 errors. Do the results change when no participants are excluded? If 10 participants were removed only from the Group model, then this is a smaller concern.

The reviewer raised an important aspect, and we wish to clarify and elaborate on this. Importantly, for the neural analysis these participants were not removed from the General Model. As the reviewer pointed out, it was important to keep the sample as large as possible to avoid losing statistical power unnecessarily. For the behavioral General Model the participants had been excluded. We apologize for this oversight.

We have now revised the behavioral General Model to include all participants. This numerically alters our results but doesn't change our main findings. Note that the effect of spontaneous speech production rate does not reach significance, however, in our previous analysis it was also not significant, as the effect did not survive the multiple-comparison correction. Thus, our overall interpretation is not affected.

In contrast, for the behavioral and neural group models, participants were dropped before conducting the analysis. The rational is the following: We ran separate models for the high and the low synchronizers. Since the 10 participants in question could not be classified reliably as either of these groups, they were excluded before running the group analysis. The reviewer's comment does underline that this important point needs clarification in the manuscript:

1. The SSS test analysis produces two results for each participant: a mean PLV (averaged across two runs) and a probability value (indicating probability of being a high synchronizer). The decision to exclude participants was thus not based on the PLV but on the probability of being a high synchronizer. This procedure was defined in previous work (e.g. Assaneo et al., 2019, Nature Neuroscience, Lizcano-Cortéz et al., 2022, STAR Protocols).
2. The variable used in the General Model mixed models is the continuous PLV. For the Group models the group assignment into LOW/HIGH is based on the binary variable derived from the probability measure.

We now clarified this in the manuscript.

> l. 119: "Consistent with previous work (3,41,57), auditory-motor synchronization, measured using the SSS-test, was consistent with a bimodal distribution (Fig 1B). In this sample, N = 27 participants were classified as high synchronizers and N = 21 as low synchronizers. N = 10 participants were excluded from consecutive Group analyses because they were not clearly classified as High/Low synchronizers, while all participants were entered in the General Models."

We adjusted the results for the behavioral General Model accordingly:

> l. 140. "Speech comprehension was estimated using an intelligibility (sentence repetition) task during the MEG recording session (Fig 3A,C). Trial-based comprehension accuracy (% words correct) was regressed against several variables (see Fig 2A, B and Supplementary Table 3) (N=55). FDR corrected p-values are reported, if not otherwise indicated. As expected, we observed a main effect of syllabic rate, with decreased comprehension for higher syllabic rates (linear: b = -77.72, SE = 1.91, $p_{FDR} < .001$; quadratic: b = -26.66, SE = 1.01, $p_{FDR} < .001$). Replicating previous findings (41), stronger auditory-motor synchronization (b = 0.05, SE = 0.01, $p_{FDR} = .001$) predicted better speech comprehension. In contrast to our previous findings, the motor production rate effect did not reach significance (b = 0.02, SE = 0.02, $p_{FDR} = .266$). In addition, faster preferred auditory rates predicted better speech comprehension (b = 0.01, SE = 0.02, $p_{FDR} < .001$). These findings were refined by two-way interaction effects of auditory-motor synchronization (PLV) ⨉ preferred auditory rate (b = 0.08, SE = 0.01, $p_{FDR} < .001$), auditory-motor synchronization (PLV) ⨉ motor production rate (b = 0.05, SE = 0.02, $p_{FDR} = .007$) and motor production rate ⨉ preferred auditory rate (b = -0.06, SE = 0.02, $p_{FDR} < .001$), and a three-way interaction of motor production rate ⨉ preferred auditory rate ⨉ PLV (b = -0.08, SE = 0.02, $p_{FDR} < .001$). A positive effect of the motor production rate was particularly observed in high synchronizers (auditory-motor synchronization (PLV) ⨉ motor production rate).The positive effect of the preferred auditory rate was stronger in individuals with high motor production rates. The 3-way interaction suggests that the interplay of auditory and motor rates is particularly evident in individuals with stronger auditory-motor synchronization. Several control variables facilitated speech comprehension: better working memory performance (b = 0.07, SE = 0.01, $p_{FDR} < .001$), shorter sentences (b = -0.12, SE = 0.02, $p_{FDR} < .001$), and time on task (i.e. suggesting performance improvement throughout the experiment, b = 0.05, SE = 0.00, $p_{FDR} < .001$)."

R1 issue 4

The theoretical assumptions and claims are a little underspecified and underexplored. Why should motor areas aid in speech comprehension? Perhaps because it is neurally efficient for oscillating circuits to be multi-purpose, or maybe because speaking, listening, and non-verbal communication are inseparable, or some other reason? Relatedly, the idea that there are two pathways—high synchronizers recruit speech-motor systems and others auditory-working memory pathways—is interesting and could be elaborated and contextualized. How does this interface with the literature? What working memory or motor action studies bear on this? What concrete predictions does it produce? The reader could benefit from more theoretical exposition and exploration, perhaps by linking this hypothesis to existing frameworks or by covering relevant literature.

We have now clarified the theoretical background and evidence for motor areas aiding speech comprehension in the introduction:

> l. 20: “Verbal communication, by requiring the matching of acoustic and articulatory representations, exemplifies action-perception interactions in humans essential for everyday behavior (1). In particular, speech production and perception are intricately interwoven. The perception of speech may rely on the identification of intended vocal tract gestures (2), allowing for the prediction of the speech signal, as considered crucial in vocal learning (3). Others assume a more moderate role of the motor system, such that it is aiding speech perception particularly in demanding listening situations (4,5). During speech production, the motor system engages in operations requiring precise timing (6,7), with these motor areas possibly being similarly recruited during speech perception (6,8,9). Specifically, the supplementary motor area (SMA) and (parts of the) inferior frontal gyrus (IFG) are assumed to generate temporal predictions about upcoming sensory events (10–15). The SMA and IFG take different roles during speech perception, with the SMA receiving input from subcortical loops serving mostly inhibitory input and the IFG more closely linked to auditory cortex processing in the temporal lobe (9,16–18). Based on the literature, on the one hand, and on our goal to focus on higher-order speech processing and sequencing (19–21), on the other, we operationalized speech motor areas as comprising the pars triangularis of the IFG as well as SMA, while not including for these analyses the region of pre central gyrus implicated in speech motor control (21,22)."

We have discussed frameworks and evidence, which is in accordance with our two-pathway interpretation of the findings:

> l. 505: “Behaviorally, particularly speech comprehension in low but not high synchronizers was predicted by working memory capacity. According to predictive coding frameworks, if an incoming signal can be predicted accurately by an internal predictive model, less sensory processing is needed and cognitive resources are released (83,84). More specifically, during speech comprehension predictions from cortical motor areas about how the speech signal sounds, may reduce the reliance on phonological storage (85). This may be an explanation for the two speech processing routes we observe, with high synchronizers relying more on predictions from the motor system, related to enhanced comprehension performance, and low synchronizers relying more on an auditory working-memory contribution. ”

Minor points

1. The letters in the text description in Figure 1 caption do not correspond to letters in the figure.

We thank the reviewer for the hint and fixed the caption accordingly.

2. It could be helpful to consistently refer to neural rates as “frequency” and behavioural rates as “rate” to avoid confusion. . For example, in Figure 5, perhaps refer to SMA/STG frequency.

We agree that this distinction is useful and have revised this throughout the manuscript.

3. In general, some of the writing could be cleaned up to disambiguate neural and behavioural variables. As another example, “PLV” is used consistently—I believe—to refer to audio-motor synchronization. But this starts to become confusing when the neural results enter the fray. For example, in Figure 5 and surrounding text, probably many readers will assume PLV refers to neural phase effects of specific regions. Perhaps call it audio-motor synchronization (PLV) or merely audio-motor synchronization without metric.

This has been revised to *audio-motor synchronization (PLV*) throughout the manuscript.

4. It took me a moment to realize that the y-label in Figure 2 goes for all subplots.

The labels were revised for Figure 2.

5. In the Group Model results (Figure 2B), the authors show memory as a predictor for low synchronizers and motor production rate for high synchronizers. The reader might wonder how memory and motor production rate predict for the other group not shown. In fact, I would suggest having one column for group models and within one subplot showing a differently coloured line for Lows and Highs. This makes the results directly visually comparable and adds information.

We revised the figure 2B to show the predictions for Highs and Lows together, Because the model predictions for memory give the impression that high and low synchronizers show differences in memory score, we also added a plot with the raw data to the Supplement (Supp. Figure 6, also see below). Additionally, statistical comparisons showed no group differences (Wilcoxon: W = 375.5, p-value = 0.188).

For consistency, we also created a new Figure 6, displaying the results from the neural data analysis with high and low synchronizers plotted in the same figure.

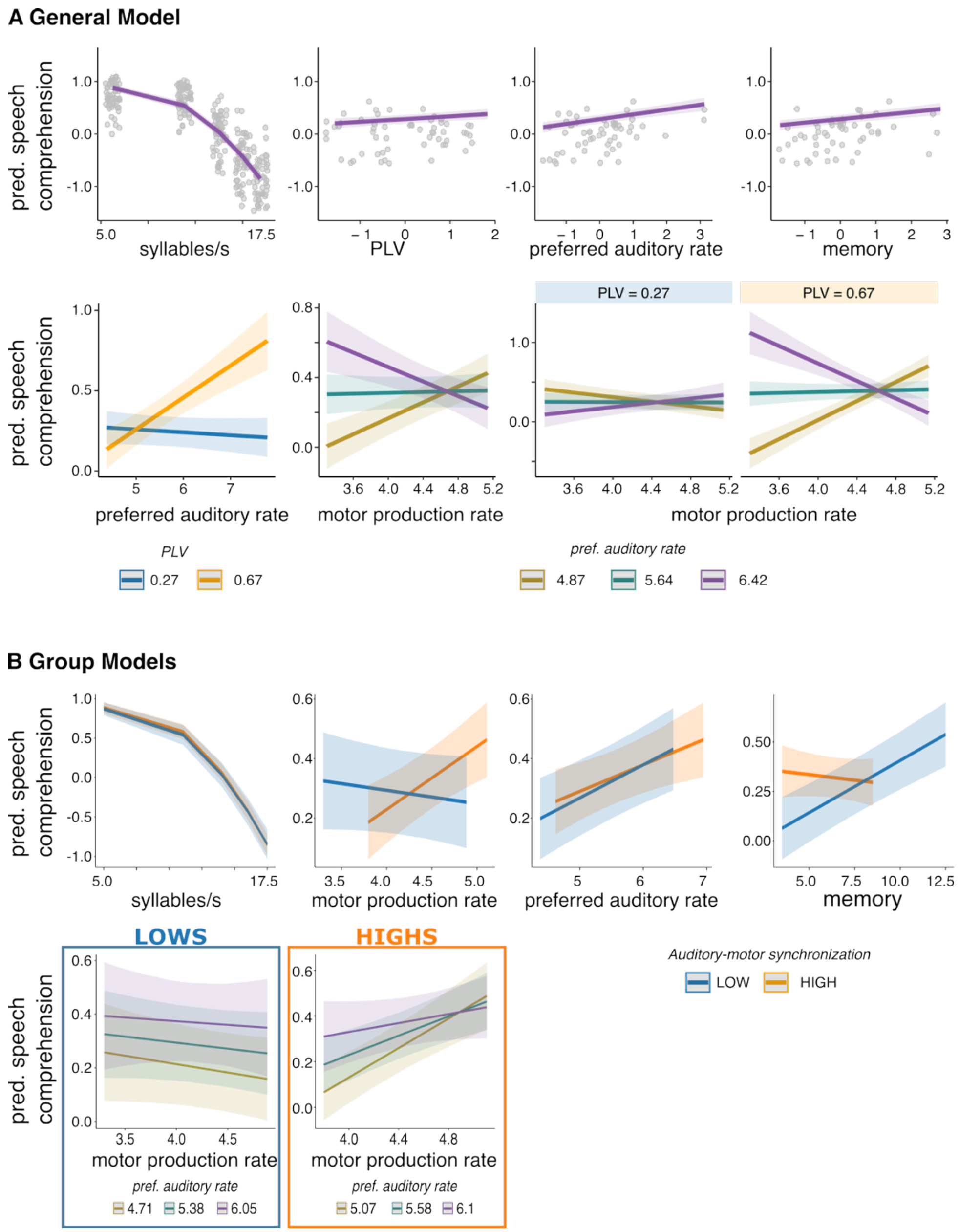

Figure 2. Main and interaction effects predicting speech comprehension in all participants (A) and in high versus low synchronizers (B). A. The general GLMM revealed a negative main effect of syllabic rate (row 1, column 1) and positive main effects of PLV (row 1, column 2), the preferred auditory rate (row 1, column 3), and memory (row 1, column 4). We further observed interaction effects (right column) between preferred motor rate and PLV (row 2, column 1), preferred auditory rate and motor production rate (row 2, column 2), and a three-way interaction of preferred auditory rate, motor production rate and PLV (row 2, column 3-4) **B.** Results from GLMMs computed separately for low (blue) and high (orange,) synchronizers. In low synchronizers, speech comprehension was predicted by the preferred auditory rate and working memory. In contrast, for high synchronizers preferred auditory rate, motor production rate, and their interaction predicted speech comprehension. For optimal comparison of effects between high and low synchronizers, all main effects of the group model are visualized in one panel.

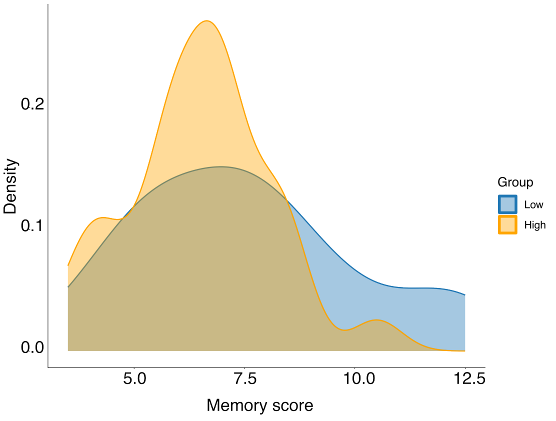

Supplementary figure 6. Distribution of memory scores. Distribution of working memory performance scores for participants classified as high synchronizers and low synchronizers. Scores are plotted separately for each group. Working memory scores do not differ between groups, as assessed using Wilcoxon Test (W = 375.5, p-value = 0.188).

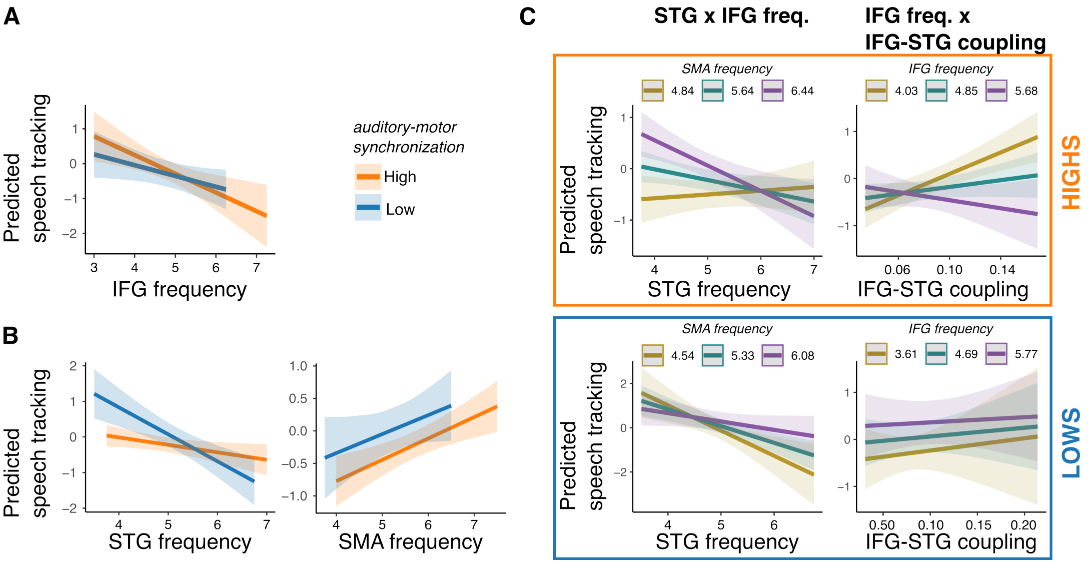

Figure 6. Speech tracking in HG and STG is predicted by different variables in low (blue) and high (orange) synchronizers. **A.** In the HG model, high synchronizers showed a negative main effect of endogenous IFG rate, whereas low synchronizers showed no significant effects after correction for multiple comparisons. **B.** In the STG model, low synchronizers exhibited a negative main effect of STG frequency, while high synchronizers showed a positive main effect of endogenous SMA frequencies. All main effects in A and B are displayed for both groups, even when significant effects were observed in only one group. **C.** In high synchronizers, the STG model additionally revealed two interaction effects involving speech motor areas that were not significant in low synchronizers: STG ╳ SMA rates, and STG–IFG coupling ╳ IFG rate. In all panels, error shades indicate 95% confidence intervals. STG = superior temporal gyrus; IFG = interior frontal gyrus; SMA = supplementary motor area.

Reviewer #2:

This manuscript describes MEG and behavioral investigation of tracking between endogenous brain oscillations and amplitude modulations in the auditory signal. The novelty of this study is the demonstration of individual differences in the endogenous rates of theta rhythm at rest and speech tracking during a sentence listening and repetition task. A behavioral test of auditory-motor synchronization was used to identify individuals with high spontaneous synchronization behavior. These individuals showed a relationship between endogenous rates of theta in the SMA and speech tracking. The relationship between theta in IFG and speech tracking was negatively correlated. Another relationship was found between working memory and performance on the speech intelligibility task in individuals who showed low auditory-motor synchronization.

This is an impressive set of related studies drawing on resting-state MEG, MEG during sentence listening and repeating, off-line behavioral measures of speech synchronization, articulation rate, and preferred syllable listening rate. There are a lot of results to report for each of these measures and then a lot of relationships and interactions to explore. Furthermore, the MEG data is drawn from at least 8 cortical areas (left and right STG, HG, SMA and IFG). The authors have done a good job to present their work clearly, though there are inevitably areas where the reader wants to know more. If further explanations or summaries of results could be provided, this would be very helpful. There are a lot of results to work through.

I have several concerns that I would like the authors to address in revision. These are mostly focused on methodological issues, decisions for analyses, and some improvements for presentation of results. I think the authors have done a good job motivating their work and discussing it:

We want to thank the reviewer for the positive evaluation and helpful feedback. All comments are addressed below.

R2 issue 1

Speech motor areas used in the analyses here were the SMA and IFG. More explanation is needed as to why those areas were selected and not, for example, ventral or dorsal premotor areas involved in speech production. Were data from these other areas also analysed?

Previous research on speech tracking and theta auditory-motor coupling during speech comprehension has focused on IFG (Park, Thut, Gross, 2018, Language, Cognition and Neuroscience; Park et al., 2015, Curr Biology), precentral brain areas or PMC (Park et al., 2015, Curr Biology; Keitel, Gross, Kaiser, 2018, Plos Biol), or used a broad speech motor ROI that jointly included IFG, PMC, SMA and other frontal brain areas (Assaneo & Poeppel, 2018, Sci Adv; Assaneo et a., 2019, Nat Neurosci).

Neural models of speech perception, suggest IFG and SMA to be central structures in the *temporal processing* of speech (Tourville & Guenther, 2011, Language and Cognitive Processes; Hertrich, Dietrich, Ackerman, 2016, Neurosci Biobehav Rev; Hickok, Vencia, Teghipco, 2023, Brain; Bullock et al., 2025, iScience), involved even during passive speech perception (Perron et al., 2024, Hum Brain Map). The SMA receives input from subcortical loops serving mostly inhibitory input and the IFG is more closely linked to auditory cortex processing in the temporal lobe (Hertrich, Dietrich, Ackerman, 2016, Neurosci Biobehav Rev). Both the IFG and SMA can be subdivided, with parts involved in control of complex sequencing, the timing of supra-second intervals and the integration of prosody and syntax (pre-SMA and IFG BA45 area) and parts involved in the timing of sub-second intervals providing and articulatory - phonological network (SMA-proper) (Hertrich, Dietrich, Ackerman, 2016, Neurosci Biobehav Rev). Given the coarse MEG measure, we did not further differentiate the subareas of the IFG and SMA. As the reviewer points out, the ventral and dorsal premotor cortex (vPMC, dPMC) (and primary motor cortex) are also considered as crucial speech motor areas involved in articulation and phoneme and syllable timing during speech perception (Hickok et al., 2023, Brain). The premotor cortex may be involved at an intermediate processing level, while IFG likely conducts higher-level processing, such as sequencing.

Given their relevance for the temporal processing of speech and their differential involvement during speech comprehension, in the current study we focused on IFG and SMA as speech motor ROIs. Other speech motor areas have been left out to streamline the already extensive analyses.

Another reason for leaving out premotor cortex is that the AAL atlas contains no dedicated ROI for the premotor cortex. Therefore, the previous literature characterized the endogenous brain rhythms only in a large precentral ROI rather than in premotor cortex, making the results for premotor cortex harder to compare (Keitel & Gross, 2016, Plos Biol; Lubinus et al., 2021, Cereb Cortex).

We revised the manuscript to clarify these points.

> l. 30: "The SMA and IFG take different roles during speech perception, with the SMA receiving input from subcortical loops serving mostly inhibitory input and the IFG more closely linked to auditory cortex processing in the temporal lobe (9,16–18). Based on the literature, on the one hand, and on our goal to focus on higher-order speech processing and sequencing (19–21), on the other, we operationalized speech motor areas as comprising the pars triangularis of the IFG as well as SMA, while not including for these analyses the region of pre central gyrus implicated in speech motor control (21,22)."

R2 issue 2

There is no analysis of a functional asymmetry between left and right ROIs. It would be interesting to know if this was a relevant factor and if not, some discussion of this is warranted. Stronger left hemisphere effects might have been expected. Also, if left/right was not a factor in the analyses, were the data from the ROIs averaged across the two hemispheres? Please clarify.

All neural analyses (tracking, coupling, spectral fingerprints) were performed for the hemispheres separately. Prior to the statistical analysis, we averaged for all these measures across the corresponding left/right hemispheric ROIs to reduce the complexity of the model. This has now been made clearer in the manuscript.

> l. 201: "To simplify the complexity of the statistical model, GCMI values were averaged across hemispheres. "

> l. 202: "GCMI values were averaged across hemispheres."

> l. 235: " From single-subject clusters, we extracted each participant's theta frequency for these brain regions and averaged these values across hemispheres (Fig 4A,C). "

Additionally, following the reviewer's suggestion to dive deeper into potential hemispheric differences we now added two analyses. First, in order to compare our findings to the previous literature, we computed a hemispheric asymmetry index (right hemisphere GCMI minus left hemisphere GCMI) for the speech tracking separately for the STG and HG ROI and high and low synchronizers. Non-parametric t-tests and FDR correction were used to test for differences in hemispheric asymmetry in high compared to low synchronizers, separately at the different syllabic rates. A figure has been added to the supplement (Supplementary Figure 4). Previous research has reported decreased right hemispheric asymmetry for high compared to low synchronizers (Assaneo, Rimmele et al, 2019, Front Integr Neurosci) and related this to stronger left-hemispheric motor top-down effects in high synchronizers. In the current study, we also find hemispheric asymmetry in speech tracking with increased speech tracking at the right hemisphere. Visual inspection suggests stronger hemispheric asymmetry at slower compared to faster syllabic scales. However, the asymmetry index was significantly larger than zero at all syllabic rates. High compared to low synchronizers showed no differences in hemispheric asymmetry. The results are discussed in the manuscript and the figure is added to the supplement (Supplementary Figure 4).

As a second novel analysis that we added to the revision, we included hemisphere and its interaction with the endogenous auditory and motor frequency and auditory-motor coupling as predictor in our neural LLM analysis general models. The results suggest overall increased right compared to left-hemispheric tracking (fixed effect of hemisphere; Supplementary Figure 5), however, no interaction with the endogenous frequency or coupling was observed. Furthermore, we added these predictors in the general model that included the auditory-motor synchronization (PLV) as predictor. Here, no interaction with auditory-motor synchronization (PLV) was observed. In summary, although we find stronger right hemispheric speech tracking, the effects of endogenous frequencies and auditory-motor coupling on speech tracking did not vary across hemispheres or synchronizer groups. This information was added to the manuscript:

> l. 330: "**Effects of auditory-motor parameters show no hemispheric asymmetry**
> Additional analyses were computed to investigate hemispheric asymmetry in speech tracking and its interaction with the auditory-motor parameters. The hemispheric asymmetry index (65) indicates stronger right hemispheric speech tracking, with strongest

effects at slower syllabic rates. Asymmetry was significantly greater than zero at most frequencies in both STG and HG (all FDR-corrected $p \leq 0.03$, strongest at 5 Hz, Supplementary Table 15, Supplementary Figure 4), whereas no significant differences were observed between high and low synchronizers at any rate (Wilcoxon rank-sum tests; all FDR-corrected $p > 0.48$). Furthermore, using LMMs, the STG general model (N = 57, Supplementary Table 113) and the HG general model (N=57, Supplementary Table 12) were computed with the additional predictors hemisphere and the interactions of hemisphere with endogenous auditory frequency, endogenous motor frequency and auditory-motor coupling. In both the STG and the HG model fixed effects of hemisphere were observed (STG: hemisphere [r], b = 0.40, SE = 0.08, statistic = 4.79, p_FDR <0.001, Supplementary Table 11; HG: hemisphere [r], b = 0.43, SE = 0.09, statistic = 4.62, p_FDR <0.001, Supplementary Table 12), indicating *stronger speech tracking in the right hemisphere* (Supplementary Figure 5). None of the interactions survived the multiple comparison control (all p > 0.138). Additionally, to assess whether effects of auditory-motor parameters varied across high and low synchronizers the STG and HG general model with the auditory-motor synchronization (PLV) interaction effects was computed with the additional predictor of the interaction of hemisphere and auditory-motor synchronization (PLV). No significant interaction with the hemisphere was observed (Supplementary Tables 13, 14). Here besides the fixed effects of the hemisphere (STG model: hemisphere [r] b = 0.4, SE = 0.08, statistic = 4.79, p_FDR <0.001, Supplementary Table 13; HG model: hemisphere [r] b = 0.45, SE = 0.09, statistic = 4.75, p_FDR <0.001, Supplementary Table 14), no interaction effects of hemisphere and auditory-motor synchronization were observed (STG: PLV × hemisphere [r]: b = 0.07, SE = 0.08, statistic = 0.93, p_FDR = 0.691; HG: PLV × hemisphere [r]: b = 0.04, SE = 0.08, statistic = 0.43, p_FDR = 0.861). "

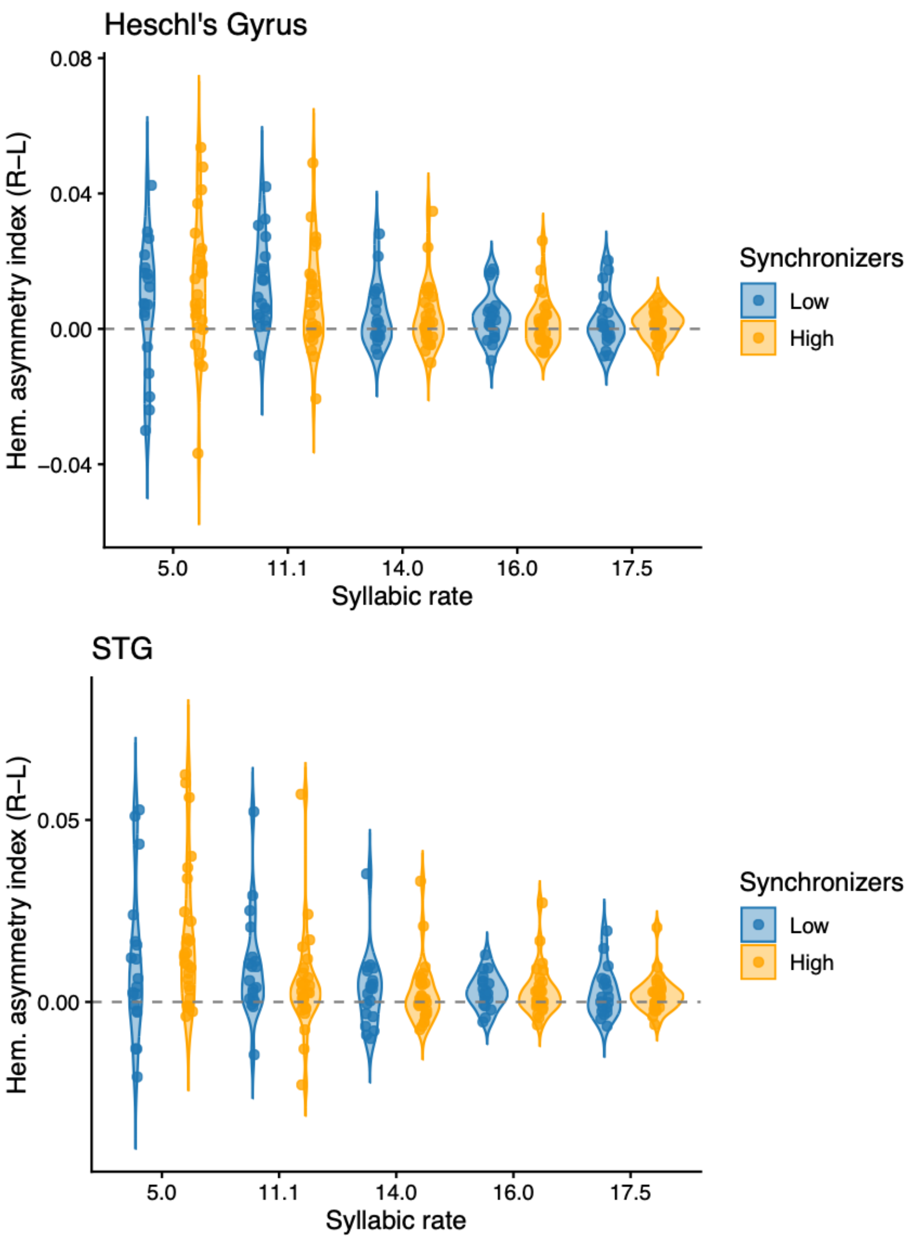

Supplementary Figure 4 Hemispheric asymmetry in speech tracking. Hemispheric asymmetry index (HAI) values are shown for high (orange) and low (blue) synchronizers across syllabic rates in Heschl's gyrus (HG; left) and posterior superior temporal gyrus (pSTG; right). Positive values indicate stronger right-hemispheric speech tracking.

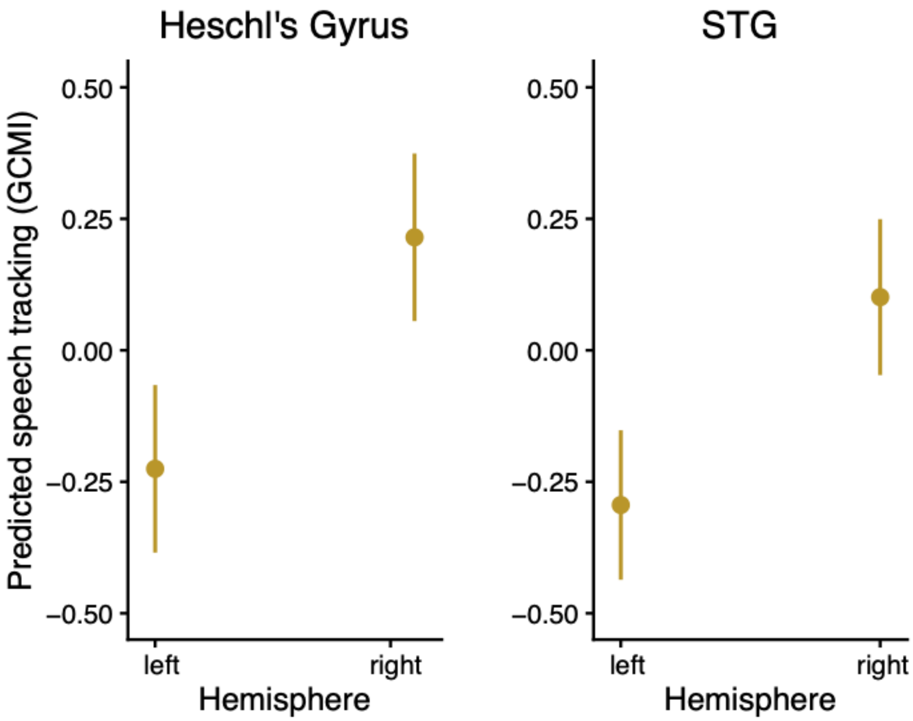

Supplementary Figure 5 Stronger speech tracking in the right HG and pSTG. Additional mixed models were computed, including hemisphere as categorical predictor. In both HG and pSTG, speech tracking was significantly stronger in the right, than the left hemisphere (see also Supplementary models 11,12).

R2 issue 3

Line 26/28 and throughout - replace motor/auditory cortices with cortical motor/auditory areas or specify the areas. For the motor areas, this encompasses primary motor cortex, premotor cortex (dorsal/ventral), supplementary motor cortex, and pre-SMA, which are in quite different locations and have varied roles in motor control. Sometimes in this paper when speech motor cortex is mentioned, it includes IFG - why not just say IFG? For auditory areas, it seems only STG and HG are referred to so just say this and be specific.

We now replaced motor/auditory cortices with cortical motor/auditory areas throughout the manuscript, whenever we are not more specific.

R2 issue 4

The authors state that quantification of individual endogenous brain rhythms is difficult - I assume they mean quantification in individuals. They identify spectral fingerprinting as a promising approach. The introduction would benefit from a brief explanation of this method and why it is promising.

To clarify this issue, we have added the following information to the manuscript:

> l. 62: " While it is relatively straightforward to identify individual peak frequencies in some cases (e.g., posterior alpha peak frequency) because those can be detected in average power spectra, this is typically more challenging in most other frequency bands and areas. We here use spectral-fingerprinting of resting state brain activity (46) to comprehensively identify individual auditory spectral peaks in a completely data-driven way. Specifically, we use time-resolved clustering procedures of normalized data that result in several frequency peaks per individual, which reflect endogenous rhythmic activity. "

R2 issue 4

I didn't understand the sentence on line 79-81 and wondered if there was something missing, specifically what is meant by "higher spontaneous preferred auditory rates" - is "higher spontaneous speech production" intended or just "preferred auditory rates"?

We clarified the wording:

> l. 96: "Our behavioral findings replicate and extend our previous study (41), by showing that higher preferred auditory rates and stronger auditory-motor synchronization predict speech comprehension."

R2 issue 5

Line 101 - 10 participants excluded for being ~50% probability of being high synchronizers. I didn't understand this sentence though I can see from the distribution a cluster of people that fall in the middle. But their values are less than 0.50 PLV if this is what 50% refers to. Please clarify and perhaps highlight the bars in the distribution that were excluded in Figure 1B.

The reviewer raised an important aspect, and we wish to clarify and elaborate on this.

Figure 1B displays the full sample of participants included in the general analysis. As correctly noted by the reviewer, participants located in the middle of the histogram, between the blue and orange distributions, represent individuals who were excluded from the group analysis. To clarify this point, we have now overlaid a box highlighting the three bars affected by these exclusions.

Importantly, a distinction must be made between the raw PLV values (range [0,1]) and the derived probability of belonging to the high- or low-synchronizer group (see Assaneo et al., 2019, Nat Neurosci; Lizcano-Cortéz et al., 2022, STAR protoc). Group assignment—and consequently exclusion from the group comparison—was based on the probabilistic classification, not directly on PLV values. Participants with a probability between 45–55% of being high synchronizers were considered to lack clear group membership and were therefore excluded from the group analysis. In the present sample, this probability range corresponded approximately to PLV values around 0.4.

l. 119: "Consistent with previous work (3,41,57), auditory-motor synchronization, measured using the SSS-test, was consistent with a bimodal distribution (Fig 1B). In this sample, N = 27 participants were classified as high synchronizers and N = 21 as low synchronizers. N = 10 participants were excluded from consecutive Group analyses because they were not clearly classified as High/Low synchronizers, while all participants were entered in the General Models. "

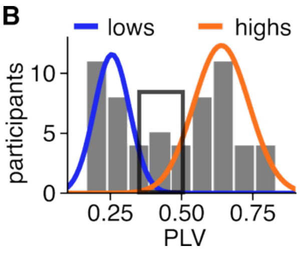

Figure 1 B. The histogram illustrates audio-motor synchronization (measured as PLV). Colored lines represent fitted normal distribution, obtained by a Gaussian mixture model. The black box indicates participants that were excluded from the group analysis, as no unique group affiliation could be determined (i.e. probability of 0.5).

Noise in the figure legend is indicated as a horizontal gray bar but I didn't understand which part of the figure it referred to. Several parts of figures contain gray sections.

We thank the reviewer for pointing this out and adapted both figure legend and caption to disambiguate. The relevant part of the caption now reads as follows:

L. 126: "**A.** We measured auditory-motor synchronization using the (explicit) SSS-test (63), wherein participants whisper the syllable /te/ (green speech signal) while listening to a syllable train (purple speech signal) embedded in noise (grey signal). They are instructed to synchronize their motor output to the auditory input. **B.** The histogram illustrates audio-motor synchronization (measured as PLV). Colored lines represent fitted normal distribution, obtained by a Gaussian mixture model. The black box indicates participants that were excluded from the group analysis, as no unique group affiliation could be determined (i.e. probability of 0.5). **C.** To quantify the motor rate, we conducted a speech motor production task. Upon being prompted with a question/statement, participants spoke freely (green speech signal) for 30s. To control for auditory feedback, noise was presented while participants spoke (grey signal)"

R2 issue 6

The figure legend to Figure 1 contains labeling errors - please correct.

Thank you for pointing this out. The labelling errors in the figure caption were corrected.

R2 issue 7

The sentence repetition test is described as a measure of "Speech Comprehension", which I found misleading, since it is simply a measure of the percentage of words repeated correctly rather than a test of any understanding of meaning. I suggest "intelligibility" is used instead of "comprehension" throughout.

The “sentence repetition” task, also termed “word identification task” has been typically used to measure speech intelligibility (Beukelton & Yorkston, 1979, J Commun Disord; Schiavetti et al., 1984, J Speech Hear Res). The performance in the sentence repetition task has been shown to be highly correlated with “information transfer tasks” that are used to measure comprehension (i.e. content questions regarding a heard sentence are asked) (Schiavetti et al., 1984, J Speech Hear Res). The “sentence repetition” task is assumed to measure complex sentence processing, while comprehension is considered a requirement, the task also taps into working memory processes. An advantage of this task is that it provides a more nuanced measure of intelligibility, compared to a binary measure. Therefore, it has been widely used as proxy for comprehension, for example in the diagnostics of specific language impairment (Klem et al., 2914, Dev Sci; Leclercq et al., 2025, HAL) or in second-language studies.

We revised the manuscript to now more cautiously describe what our task measures and that we use it as proxy for comprehension:

> l. 87: “Here, we use a behavioral and MEG approach in a relatively large sample, combining spectral fingerprinting of individuals’ MEG resting-state brain activity with MEG data recorded during a speech intelligibility task (see Fig. 3 A). As a speech intelligibility task, we used a *sentence repetition task* to estimate individual’s speech comprehension performance."
>
> l. 611: “To measure speech comprehension, participants performed an intelligibility task (89,90; Fig 4A). The performance in the sentence repetition task has been shown to be highly correlated with “information transfer tasks” that are used to measure comprehension (i.e. content questions regarding a heard sentence) (90). Therefore, it has been widely used as proxy for comprehension (e.g., 91)."

R2 issue 8

Line 144 - please explain the motor production rate x preferred auditory rate interaction effect in high synchronizers.

We have added the following explanation to the manuscript:

> l. 174: "Thereby, individuals with high motor production rates showed high performance independent of the preferred auditory rate. In contrast, in individuals with low motor production rates, higher preferred auditory rates were beneficial."

R2 issue 9

What is Gaussian-Copula Mutual Information and what benefits does it offer the analyses presented here? What does it mean that it was normalized using surrogate data?

To clarify, in this work, both speech tracking and auditory-motor coupling were quantified using Gaussian-Copula Mutual Information (GCMI). GCMI is an information-theoretic measure that estimates the mutual information between two continuous variables after applying a Gaussian copula transformation. This approach ranks the data (to make them marginally uniform), maps them to a Gaussian distribution, and then computes mutual information under the assumption of Gaussian dependencies. GCMI is robust to differences in marginal distributions and provides a lower-bound estimate of mutual information for continuous, potentially nonlinearly related variables (Ince et al. 2017, Hum Brain Mapp).

After calculating GCMI, these values are often normalized to account for baseline differences using surrogate data. Surrogate data refers to artificially generated datasets that preserve certain statistical properties of the original data (e.g., amplitude distribution or autocorrelation structure) but destroy the specific dependencies or relationships under investigation. In our case, surrogate data were created by segmenting the concatenated trials of each condition, shuffling the segments for each (N=500) iteration and estimated GCMI between ROI source time-series and shuffled speech time-series (for speech tracking) or motor ROI time series shuffled auditory ROI time series (for auditory-motor coupling.

We clarified the explanation of GCMI in the methods sections:

> l. 803: "Speech tracking was estimated using information theory (105,117,118). Specifically, we computed gaussian-copula mutual information (GCMI, 64) to assess statistical dependencies between speech envelopes and source-localized neural activity in auditory and motor ROIs. GCMI capitalizes on the concept of gaussian copulas which is a statistical description of the relationship of two variables regardless of their respective marginal distributions. Mutual information (MI) can be computed as the negative entropy of a statistical copula. This approach ranks the data (to make it marginally uniform), maps them to a Gaussian distribution, and then computes mutual information under the assumption of Gaussian dependencies. This facilitates estimation of MI because 1) no assumptions of the marginal distributions of the variables are needed and 2) the computation is efficient as a Gaussian parametric estimation can be employed. A further advantage of the GCMI approach is that it lends itself naturally to dealing with multivariate data (64)."

l. 838: " **Mutual information – surrogate data**

We tested whether speech tracking and auditory-motor coupling exceeded chance-level within each condition by comparing the observed MI values to the 99th percentile of surrogate data. Surrogate data refers to artificially generated datasets that preserve key statistical properties of the original data (e.g., amplitude distribution or autocorrelation structure) while disrupting the specific dependencies or relationships of interest.

Surrogate data for both analyses (speech tracking and auditory-motor coupling) were generated by segmenting the concatenated trials of each condition, shuffling segments across iterations (N=500), and computing GCMI between one intact and one shuffled timeseries. For speech tracking, GCMI was estimated between each source time series and the shuffled speech time series. For auditory–motor coupling, GCMI was computed specifically between the motor ROI source time series and the shuffled auditory ROI time series. Segment length was condition-specific and set to 80% of the cycle length corresponding to each syllabic rate (5 Hz = 160.0 ms; 11 Hz = 72.72 ms; 14 Hz = 57.1 ms; 16 Hz = 50 ms; 17.5 Hz = 45.7 ms).

We used the surrogate distribution to create z-transformed GCMI values by subtracting the mean of the surrogate distribution from the observed GCMI values and dividing by the surrogate's standard deviation. The normalization was performed within participants for each parcel, condition, and syllabic rate. GCMI values varied systematically as a function of delay and condition; we extracted GCMI for each condition at the delay that maximized the GCMI values."

R2 issue 10

Line 192 - describe what is meant by the "spectrum showed an offset" and why this led to using the non-normalized GCMI values.

For the auditory-motor coupling analysis, normalizing the GCMI values by the surrogate data introduced a large difference between the syllabic rate conditions that was not present in the non-normalized data. Specifically, the GCMI values for the 5 syll/s condition were higher than the values for the other conditions (i.e. showed an offset), with increasing differences for higher frequencies. Because the normalization changed the shape of the GCMI spectrum drastically (See Supplementary Fig. 2 pasted below), we chose to perform all subsequent analysis on the non-normalized data. Please note that the normalization is not a required step but rather a way to improve the signal to noise ratio.

To visualize the offset, we provided both the normalized and non-normalized GCMI spectra in the Supplementary Materials (see Supplementary Figure 2).

This has been clarified in the manuscript:

l. 224: " However, the normalized GCMI spectrum showed an offset (i.e., a large difference in GCMI values between the syllabic rate conditions that was not present in the non-

normalized data) in the 5 syllables/s condition relative to the faster conditions (see Supplementary Fig 2B). Because the normalization changed the shape of the GCMI spectrum drastically, we treat the normalized data cautiously and conduct subsequent analyses on the non-normalized GCMI values for both speech tracking and auditory-motor coupling."

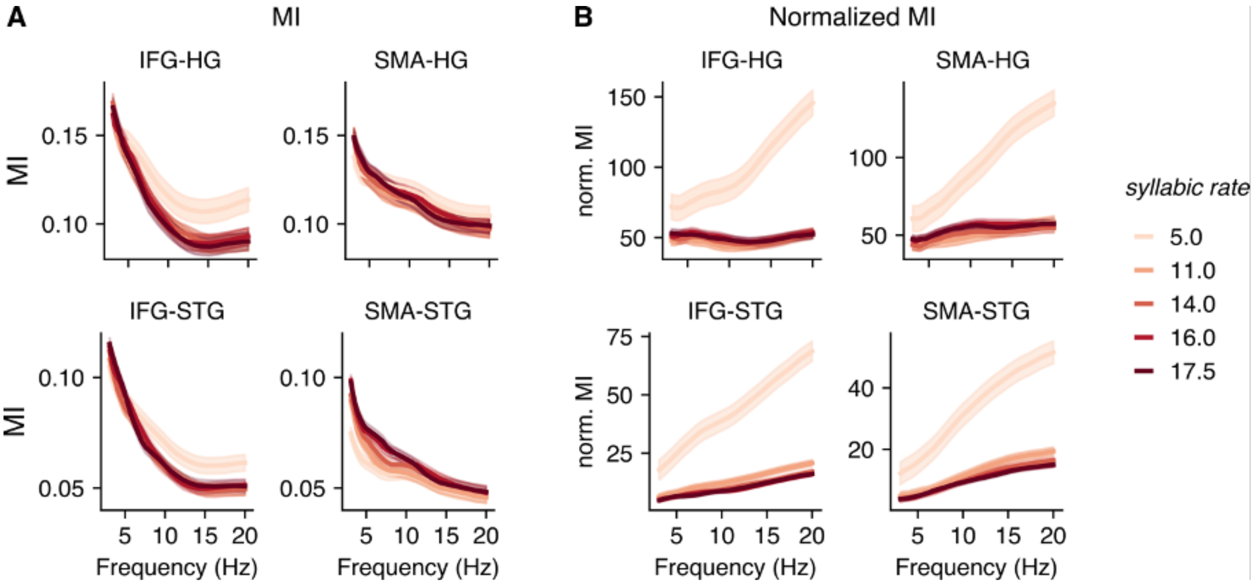

Supplementary Figure 2. Raw MI spectra for auditory-motor coupling. Line plots illustrate raw (A) and normalized (B) auditory-motor coupling (MI) between different auditory and motor brain areas as a function of frequency: IFG-HG, SMA-HG (top), IFG-STG , SMA-STG (bottom). Lines are color-coded according to the syllabic rate of sentence stimuli and shaded error bars represent standard error of the mean across participants. HG = Heschl's gyrus, STG = superior temporal gyrus; IFG = interior frontal gyrus; SMA = supplementary motor area.

R2 issue 11

Those with lower endogenous auditory theta frequency showed better speech tracking - is that because speech is typically lower than 5Hz?

This is a relevant question, we now included an interpretation in the discussion:

l. 462: "It is possible that for individuals who more strongly rely on the auditory system for speech processing and recruit the motor system less, a lower endogenous theta frequency is advantageous because it is close to the natural syllabic rate of speech (~5 Hz). For individuals who strongly recruit the motor system instead, because cortical auditory and motor areas more strongly interact, whether lower or higher endogenous theta frequencies are advantageous may depend on the frequency of the endogenous theta motor rhythm (as suggested by the interaction effect of auditory and motor frequency)."

R2 issue 12

What drove the choice of syllable rates? 11-17.5 syllables/s is VERY fast and the step up from 5 to 11 is curious - why nothing in between? Also, people tend to learn to understand compressed speech very quickly. Was there a training/exposure period to avoid learning effects during the test?

The selection of syllabic rates was informed by our previous study (Lubinus et al., 2023, *Proceedings of the Royal Society B*) and a pilot study conducted in preparation for it. In the pilot, we tested syllabic rates ranging from 5.0 to 12.0 syll/s but found that comprehension remained near ceiling even at the highest rates. Since the motor system's influence may be most pronounced under more challenging listening conditions (Stokes et al., 2019, Psychonomic Bulletin & Review), we adjusted the syllabic rates in both studies to better capture the full range of effects.

Participants performed a brief training prior to the main task. However, this training likely did not suffice to fully avoid trainings effects. To control for such effects statistically, we introduced trial order as random effect into all our models. This was clarified in the manuscript.

> l. 607: "The selection of syllabic rates (with distributions around five syllabic rates: 5.00, 11.00, 14.00, 16.00, 17.50 syllables/s) was informed by our previous study to capture the full range of variance in comprehension (41)."

R2 issue 13

Preferred auditory rate was 3-8.5 syllables. How many trials was this based on? etc.

We clarified this in the methods:

> l. 638: "On each trial, we presented two versions of the same sentence, randomly ordered, differing only in syllabic rate. After stimulus presentation, participants indicated which of the two stimuli they preferred via button press. Stimuli were presented at twelve syllabic rates from 3.0 to 8.5 syllables/s (in steps of 0.5 syllables/s). Each syllabic rate was paired against every other rate, resulting in 132 unique trials. Trials were presented in blocks of 30 trials, with self-paced breaks in between. Crucially, the trial sequence was randomized, without grouping by syllabic rate."

R2 issue 14

For the motor localizer - was there movement of the articulators or was it imagined? I understand there was no vocalizing so no auditory feedback, but would there be somatosensory feedback?

Yes, for the motor localizer participants performed the movement such that somatosensory feedback would have been present. Specifically, participants were instructed to perform the

movement required to speak the syllable without vocalizing. However, we wish to highlight that the motor localizer was not used in any of the analyses and was only mentioned to disclose the complete protocol. It was not used in this manuscript, given that previous fingerprinting studies have not specified spectral profiles for motor localizers (Keitel & Gross, 2016, PLOS Biology; Lubinus et al., 2021, Cerebral Cortex).

> l. 676: "Third, participants performed a motor localizer task (~12 min) during which they repeatedly articulated syllables (i.e. performed the movement) without vocalizing. On each trial, one out of three syllables (/pa/, /ta/, /sa/) was articulated for 3 s and each syllable was repeated in 50 trials. Note that the motor localizer data was not analysed in this study. "

R2 issue 15

Please report the degrees of freedom for the statistics and effect sizes.

Computing the degrees of freedom (dfs) in LMMs is not as straight forward as in linear regression or analysis of variance. In mixed models, the sampling distribution of fixed-effect estimates depends on estimated random-effect variances. Because these variance components are themselves estimated from the data, denominator degrees of freedom cannot be determined analytically and must be approximated. We use the Satterthwaite-approximation to estimate the dfs of the fixed effects. The dfs were added to the supplementary tables. Several effect size metrics have been suggested for LMMs. Here we estimate the partial eta-squared (ηp^2) values. We report ηp^2 for fixed effects because it provides an interpretable estimate of the magnitude of effects and allows comparison with prior ANOVA-based literature

This has been revised in the manuscript:

> L 917: "Statistical inference was conducted using the lmerTest package, using the Satterthwaite's method to approximate the denominator degrees of freedom. Model coefficients (beta), standard errors (std. Error), t-values, p-values (p, FDR corrected p-values, p_{FDR}), and estimated degrees of freedom (df) are reported in the supplementary tables. As measure of effect size, partial eta-squared (ηp^2) values are reported using the *eta_squared()* function."

R2 issue 16

I struggled to understand the interactions. Were the control variables included in the full models? What is "occurrence late in the experiment" (e.g. line 133) and why was it included here? Does it refer to a practice effect or fatigue?

Yes, control variables were included in the full models.

This has been clarified in the methods and is stated in the tables in the supplementary material.

l. 161: “[...] and time on task (i.e. suggesting performance improvement throughout the experiment, $b = 0.05$, $SE = 0.00$, $p_{FDR} < .001$).”

R2 issue 17

For the figures, it would be good to see the raw data in scatterplots. I appreciate that the ones split by preferred motor or auditory rate will be a bit complex but for graphs showing the simple linear regressions would be helpful if we could see the raw data.

We now revised figure 2A and figure 5 to show the single subject raw data in scatterplots overlayed.

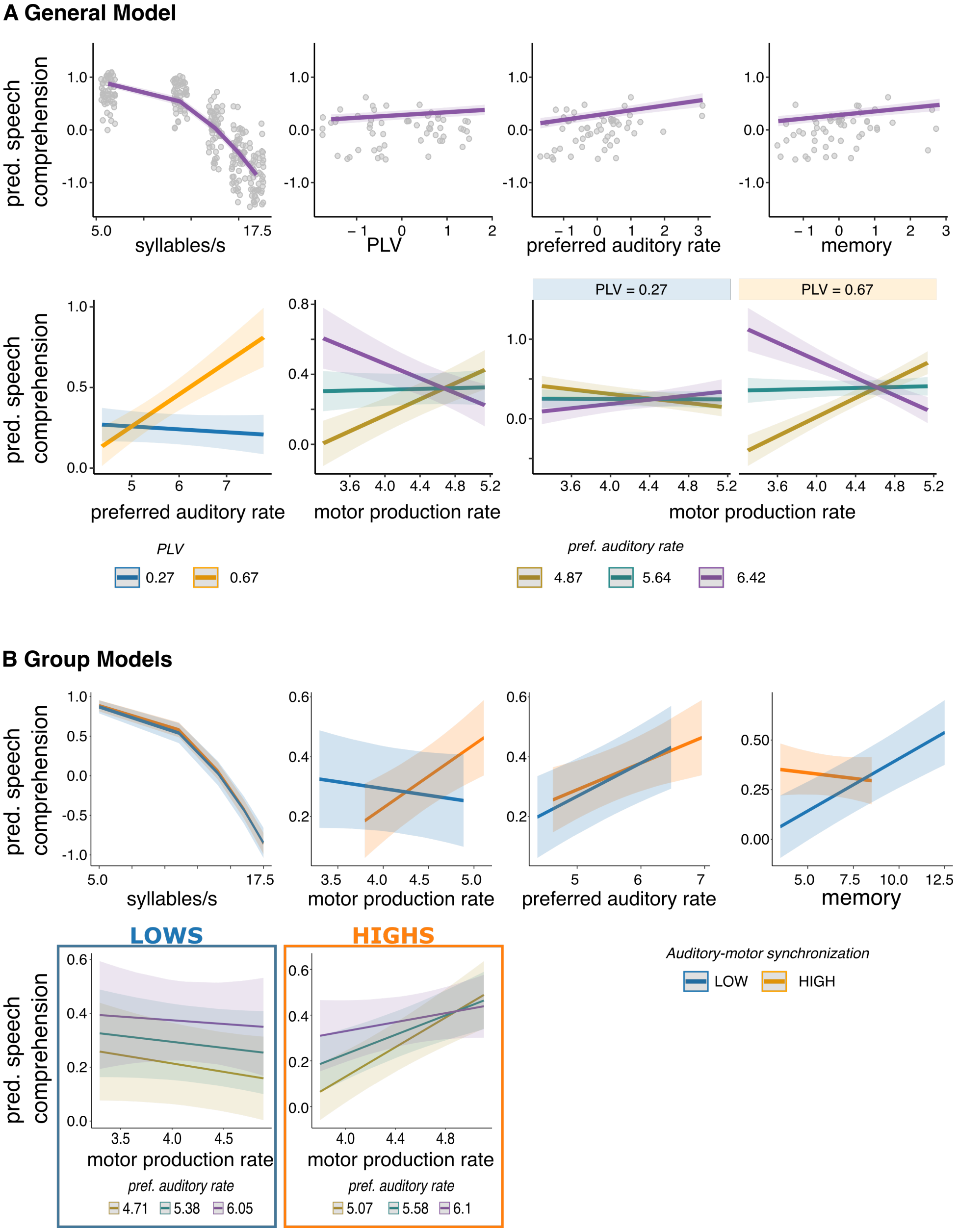

Figure 2. Main and interaction effects predicting speech comprehension in all participants (A) and in high versus low synchronizers (B). […] The raw data for each participant (dots) were scaled and plotted with the model predictions. For the speech comprehension performance values were averaged across trials for each participant […]

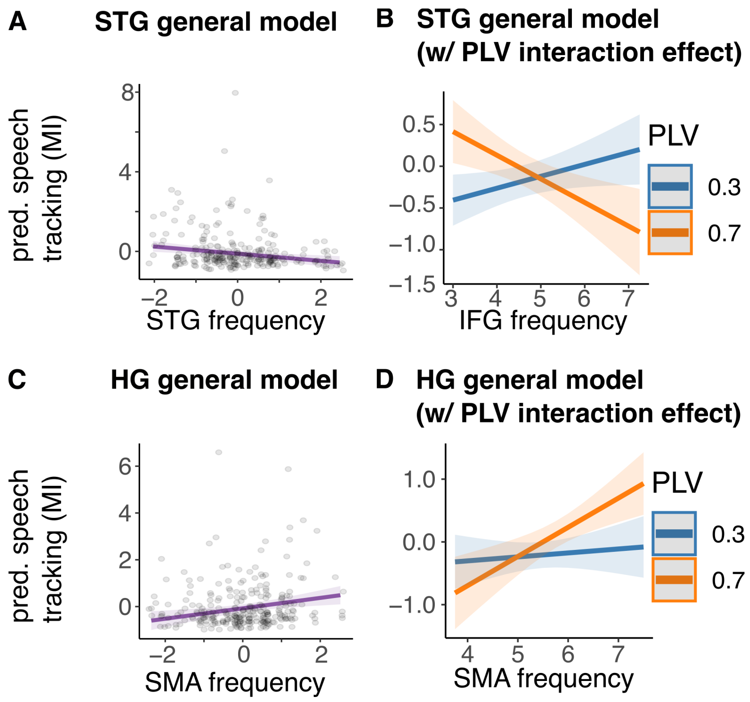

Figure 5 Speech tracking in STG and HG is predicted by distinct auditory and motor variables. […] The raw data for each participant (dots) were scaled and plotted with the model predictions.

Reviewer #3:

This paper by Lubinus et al. investigates endogenous theta oscillations in auditory and motor areas, investigated with MEG and with behavioral experiments. Behaviorally, the paper reports relationships between perceptual factors like speech comprehension, motor production rate, working memory, and auditory motor-synchronization. With MEG, relationships are reported between the peaks of spectral clusters in auditory and motor areas, neural tracking of speech, and auditory-motor coupling. Investigating both behavioral and physiological parameters is a strength of the paper. The experiments are run with a large sample, and I applaud the others for taking on such an expansive study.

We want to thank the reviewer for the positive evaluation and helpful feedback. All comments are addressed below.

<u>R3 issue 1</u>

There are a few key aspects of the study, however, that dampened my enthusiasm. The thing I was most excited about when I first read the title and abstract is that there was a correlation between resting state oscillatory activity and speech perception. Unfortunately, I was mistaken.

There was no correlation between the behavioral and MEG measures. This is not obvious from the title and is not mentioned in the abstract. It does not come up until the last paragraph of the results section (297-309), and then once more in the last few lines of the discussion (426-420). The neurophysiological measurements "predict speech tracking during comprehension," (7-8) but do not actually predict comprehension or any other behavioral measures. The next two sentences (9-12) talk about how behavioral data "align" with the neural data and "provide compelling support for oscillatory accounts of auditory-motor interactions during speech perception." I did not find this support in the paper, even though I thought I would from the title and abstract.

We now revised the analysis of the relationship between our neural auditory-motor estimates and the behavioral speech comprehension performance to provide a more extensive picture. Using LMMs, we predict comprehension performance in the speech intelligibility task by the speech tracking (STG/HG), the endogenous frequencies of cortical auditory (STG/HG) and motor areas (IFG, SMA), the auditory-motor coupling (STG/HG with, IFG and SMA), as well as the syllabic rate and random structures. LMMs were computed separately for STG and HG. This has been revised in the manuscript as follows.:

> l. 357 ff.: “**Relationship of neural auditory-motor parameters and comprehension**
> For both behavioral and neural analyses the effects exhibited similar patterns (Figures 2B and 6), with effects related to endogenous motor brain rhythms observed predominantly in individuals displaying stronger behavioral auditory-motor synchronization (high synchronizers). To directly test the relationship between the neural estimates and speech comprehension, LMM analyses were conducted separately for STG (N=48) and HG (N=52), with performance in the speech intelligibility task predicted by the syllabic rate, the neural tracking in auditory brain areas (HG, STG), the frequencies of auditory (HG, STG) and speech motor areas (IFG, SMA), as well as the auditory-motor coupling. Additionally, we performed LMM analyses with the additional predictor auditory-motor synchronization (PLV) and its interaction with the other parameters. The only significant effects that were observed were effects of the syllabic rate, with higher syllabic rates related to lower comprehension (HG: linear: b = -13.68, SE = 0.5, statistic = -27.18, p_FDR <0.001; quadratic: b = -6.58., SE = 0.32, statistic = -20.81, p_FDR <0.001; STG: linear: b = -12.8, SE = 0.52, statistic = -24.48, p_FDR <0.001; quadratic: b = -6.26, SE = 0.32, statistic = -19.79, p_FDR <0.001; Supplementary Tables 16, 18; for models with PLV included similar effects were observed, Supplementary Tables 17, 19).
>
> Additionally, to further explore the relationship between the neural estimates and comprehension performance, the analyses were inspected as exploratory analyses without FDR correction. For the HG model findings revealed a positive effect of SMA coupling (b = 0.05, SE = 0.02, statistic = 2.05, p = 0.042) and a negative effect of HG speech tracking, with higher coupling and lower tracking related to higher comprehension (b = - 0.1, SE = 0.05, statistic = - 2.11, p = 0.036, Supplementary Table 16; similar results were observed for the model with auditory-motor synchronization (PLV) predictors included, Supplementary Table 17). For the STG model, a positive effect of SMA coupling was

observed (b = 0.06, SE = 0.02, statistic = 0.1, FDR = 0.027, Supplementary Table 18). We found no interactions with auditory-motor synchronization (PLV, Supplementary Table 19)."

l. 408.: "The behavioral findings paralleled the neural results by showing similar patterns of results for high and low synchronizers. Although we found no evidence for a significant correlation between neural estimates and comprehension performance, an exploratory analysis suggests such a relationship may be present, albeit weak."

l. 444: "Previous studies (36) reported that comprehension was predicted by neural tracking. Others, however, did not report a correlation, pointing towards a more complex link between comprehension and tracking (67,69–71). Although comprehension and neural tracking both declined at higher rates, we find no significant correlation between them. An exploratory analysis (without multiple comparison control) reveals reduced neural tracking in Heschl's Gyrus was related to higher speech comprehension. However, these findings need to be interpreted cautiously. Thus overall, the relationship between neural tracking and comprehension seems to be complex, likely due to both measures being affected by various factors differently (72)."

l. 547: "We found no direct relationship between the auditory-motor neural estimates and speech comprehension. An exploratory analysis (without control for multiple comparisons) however, suggests that lower speech tracking in Heschl's Gyrus was related to higher performance in the intelligibility task. Furthermore, overall higher auditory-motor coupling between SMA and Heschl's Gyrus and SMA and STG was related to higher comprehension."

R3 issue 2

Another set of issues is with the spectral analysis of supposedly endogenous resting state activity. First, with how spectra were calculated (719-728). The windowing does not make sense. Fourier spectra can't be computed with logarithmic frequency resolution. The Fourier transform will have linear frequency spacing with a resolution of 1 / duration Hz (1.25 Hz for 0.8 s, or 0.5 Hz if zero-padding to 2 s windows, but that padding does not actually add resolution, simply interpolates in the frequency domain). The DPSS the tapers are windows and should be applied to data of the same length, not 0.8 s of data surrounded by 0.6 s zeros on either side. In this manner you are not actually using DPSS tapers—you're using DPSS tapers multiplied by a rectangular window of shorter duration, which destroys their properties. What's more, 0.8 s seems far too short for analyzing oscillations anyway—that's only a few cycles at the theta frequencies of interest. These spectra are then ratio-normalized to all the other spectra, which could allow spectral peaks to appear where none were before.

We appreciate this insightful comment but believe it reflects a misunderstanding stemming from how we originally formulated that section. To clarify, we followed the procedure introduced by Keitel & Gross (2016, PLOS Biol). Specifically, we used Fourier transforms of DPSS taper-weighted

time segments as implemented in the fieldtrip toolbox (version 20220617; ft_freqanalysis function). In this function, zero-padding is applied to the taper-weighted segments after tapering but before the FFT. This is a standard procedure that preserves the DPSS tapers. We have now revised the manuscript to make this more explicit.

About the other issue - “Fourier spectra can't be computed with logarithmic frequency resolution” We have clarified that specific frequencies from the Fourier spectra were selected (again using fieldtrip functions) in a logarithmic manner.

The manuscript now reads the following way (ll.):

> l. 877: “We used Fourier transforms of DPSS taper-weighted time segments (DPSS multitaper, 2 tapers, +/- 2 Hz spectral smoothing). DPSS multitaper were applied for spectral smoothing. Complex Fourier spectra of the DPSS taper weighted segments were computed in sensor space for individual epochs (0.8s epochs were zero-padded to a duration of 2s after the DPSS taper were applied). Zero padding was performed to retrieve frequency bins of the Fourier transform output with 0.5 Hz spacing. Frequencies at 1-120 Hz with a logarithmic frequency spacing were selected for further analyses in the spectral profile clustering analysis.”

We used a window of 0.8 seconds for the spectral profile approach. The original paper (Keitel & Gross, 2016, PLOS Biology) used a 1 second window. However, their findings were replicated by Lubinus et al. (2021, Cerebral Cortex) with a window of 0.8 seconds. We adopted the settings from Lubinus et al. The reasoning behind the shorter window is a tradeoff between the amount of trials and the window length. Both Lubinus et al. and our current study used slightly shorter overall resting state recording durations compared to Keitel & Gross (2016), rendering the shorter window length suitable. Note that in Lubinus et al. compared to Keitel & Gross less neural power in the lower delta-band (< 1 Hz) was observed, which can be explained by the lower spectral resolution. For our 5 syllables per second condition the 0.8 seconds allows for ~4 cycles, whereas 3 cycles has been typically recommended as minimum length (Tallon-Baudry, Bertrand, 1999, Trends Cogn Sci). Thus, while this choice limits our frequency resolution it increases our temporal resolution."

Regarding the concern that the normalization could introduce new peaks, this is highly unlikely. The spectra are normalized using the average spectrum of all other spectra across brain areas, which are predominantly flat. A new peak would result from an average spectrum that shows a pronounced negative peak, i.e. a trough, which does not occur in the data. It is highly unlikely that this introduced new peaks. What can happen occasionally is that a flat minor trough is introduced in the alpha range (e.g. see for example the lowest black spectrum in Figure 4A left, which shows a little alpha trough), but as we only analyzed peaks, this did not affect our results.

R3 issue 3

The second issue is with the spectral data clustering. All of the normalized spectral shapes from the 0.8 s segments above were clustered using cosine distance and k-means. The peak (or largest

peak if there were multiple) of the component that fell within the defined theta range was chosen. Only the theta clusters are shown in most of the figures, except for a couple others showing peaks seemingly in the delta, alpha, and beta range in Figure 4A, which give the impression that the oscillations are distinct separable. What did the other clusters look like? Were they also orderly peaks or were they more complex, and what would the interpretation of that be? What did the overall normalized power look for all segments? Was there any indication of distinct oscillations there? Why pick one cluster when there were multiple in the theta range instead of combining them? Would that change the results?

We thank the reviewer for this thoughtful comment and fully agree that examining the full set of spectral profiles is important for putting the clustering results into context. To support this, we had included the complete set of normalized spectral clusters across the full frequency range in the original submission (see Supplementary Figure 3, below for convenience). This figure illustrates that, in line with previous findings (Keitel & Gross, 2016, Plos Biol, Lubinus et al., 2021, Cereb Cortex), the spectral profiles were not only characterized by theta clusters but also clusters with peaks in other frequency bands (e.g., delta, alpha, beta), as well as more complex spectral patterns.

Normalized power spectrum:

We agree with the reviewer that combining multiple theta bands would be an alternative strategy. However, the purpose of this study was to assess whether the ability of one's theta rhythms to track fast speech was related to the rhythm's endogenous frequency. Where there was more than one theta cluster present, the cluster with the highest amplitude was selected, under the assumption that the rhythm with the highest amplitude reflects the most dominant theta rhythm, the least prone to noise). Combining multiple rhythms with different peak frequencies would have altered the frequency estimation. Nevertheless, we acknowledge that alternative strategies to handle multiple peaks might be possible and could be explored in future research.

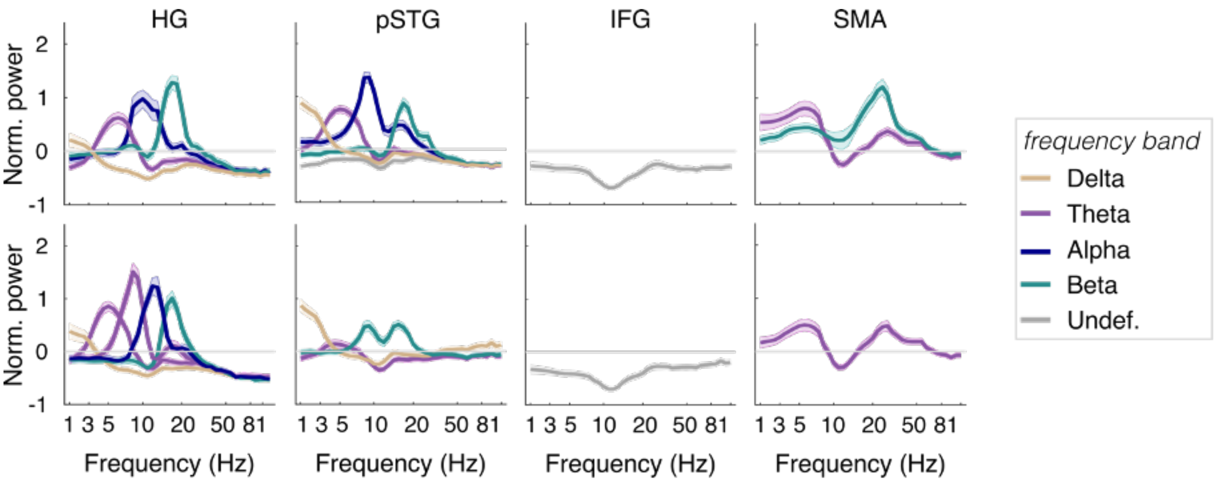

Supplementary Figure 3. Group-level spectral fingerprints in the left (top) and right (bottom) hemisphere. Clusters are color-coded according to their peak frequency (legend on the right). Shaded error bars reflect standard error of

the mean across participants. HG = Heschl's gyrus; STG = posterior superior temporal gyrus; IFG = interior frontal gyrus; SMA = supplementary motor area.

R3 issue 4

The endogenous oscillations that are central to the paper are a result of a many-steps-long, complex analysis chain. It's not clear to me that if random, non-neural data with the same overall power spectrum were put through this chain that endogenous oscillations wouldn't also result from that, which would complicated the interpretation of this study's findings quite a bit.

The spectral profiles derived from the clustering approach are specific to certain brain areas, as they enable the classification of brain areas. This finding has been replicated across multiple studies, including the present work, where our spectral profiles closely match those previously reported. This consistency suggests that the approach reliably captures the distinctive profiles of brain rhythms of a brain area.

The clustering procedure distinguishes different aspects of the power spectrum by clustering across frequencies and time. Thus, the spectral profiles reflect brain rhythms that may or may not be complex. Importantly, endogenous brain rhythms can vary in their characteristics and do not need to be strictly periodic, making the distinction between "true oscillations" and other brain rhythms non-trivial.

Regarding random non-neural data: As the procedure clusters across both frequency and time, and because spectral profiles reflect only those brain rhythms that occur consistently over time, random noise with an identical power spectrum would be expected to produce markedly different spectra.

R3 issue 5

One final issue is the way some of the statistics are reported. False discover rate (FDR) correction was performed on the statistical tests, and p_FDR is reported when it was significant. However, in some places, the result is described as meaningful and an uncorrected p is given to support this. In the first instance this is followed by a correcting sentence pointing out that the effect actually was not significant (234-236), but in the second case it is not (256, and the result is then expounded upon through line 259). As a reader I should not have to zoom in on the fine print to properly understand the findings of a scientific paper—the description of the findings should align with the statistics.

We removed all reporting and interpretation of effects that were non-significant with FDR correction. Now only effects are reported that survived the FDR control. In the supplementary tables both p-values, FDR corrected and un-corrected, are reported.

However, we state one exploratory analysis of the neural-behavior relationship where no FDR is applied. This is clearly marked as an exploratory analysis.
