# Supplementary material for: Endogenous auditory and motor brain rhythms predict individual speech tracking
Source: PLoS Biol. 2026 Jul 31;24(7):e3003924. doi: 10.1371/journal.pbio.3003924 (PMC13446743; doi:10.1371/journal.pbio.3003924)
Supplement: Supplementary file 36 [file pbio.3003924.s037.pdf]

We want to thank the Editors for their time and effort.
All requests (including the technical ones) are answered in the manuscript and below in the response letter. In summary, we clarified that we found no detectable relationship between the neural data and behavioral readouts. We revised the competing interest statement to state our PLOS Biology editor/freelancer work. We revised our data availability statement. All relevant data and code are now available at OSF, with the link provided. We revised the manuscript with respect to the technical requirements (this included updating an icon in Figure 1, adding an extra ethics statement section, citing the supplementary tables at the end of the manuscript)

* The sentences starting from line 11 in the abstract need to be revised, because the current experimental evidence does not support the claims that "these findings align with participants' behavioral data...". Please make it clear to the reader that, based on your statistical analysis, there is no detectable relationship between the neural data and behavioral readouts.
This has been revised.

> l. 12: "Behaviorally, higher auditory-motor synchronization was related to higher comprehension, with effects of the spontaneous speech motor production rate only in high synchronizers. Working memory capacity predicted speech comprehension performance only in individuals with low auditory–motor synchronization profiles. No significant relationship between the neural data and behavioral readouts was observed."

* The same for lines 96-100 in the introduction.
This has been revised.

> l. 102: "Our behavioral findings replicate and extend our previous behavioral study (41), by showing that higher preferred auditory rates and stronger auditory-motor synchronization predict speech comprehension. In contrast, higher spontaneous speech motor production rates only predicted comprehension in parts of the population (high audio-motor synchronizers), with low synchronizers instead showing predictive effects of their working memory capacity. No detectable relationship between the neural data and behavioral readouts was observed."

* The sentence in lines 107-109 ("Although we found no evidence...") could simply be removed from here. This is also true for the sentences in lines 418-422.
The sentences have been removed.
We assume the latter (i.e. lines 418-422) refers to lines 414-416 (the sentence "The behavioral findings paralleled the neural results by showing similar patterns of results for high and low synchronizers." was removed)? If another sentence was meant, we are happy to revise this.

* Please also rephrase the sentences in lines 566 to 570.
This has been revised by removing the sentence part about working memory performance.

> l. 589: "Our findings are consistent with an oscillatory model of auditory-motor interactions during speech comprehension. While some individuals recruit the speech motor system (SMA, IFG), likely providing temporal predictions to enhance comprehension, others seem to predominantly rely on auditory processing."

Note that we have revised another instances of a similar statement:
l. 379: "Relationship of neural auditory–motor parameters and comprehension"

* Please add the links to the funding agencies in the Financial Disclosure statement in the manuscript details.
The following links have been added to the Financial Disclosure statement.
MRC - https://www.ukri.org/councils/mrc/
RSE: https://rse.org.uk/
MPIEA: https://www.aesthetics.mpg.de/en.html

* Please mention in the competing interest statements that David Poeppel is a member of PLOS Biology's editorial board and that Johanna Rimmele has done freelance work and acted as a Guest Editor for PLOS Biology.

The competing interest section now reads:
"David Poeppel is a member of PLOS Biology's editorial board. Johanna Rimmele has done freelance work and acted as a Guest Editor for PLOS Biology. Otherwise, the authors declared no competing interests."

* DATA POLICY:
You may be aware of the PLOS Data Policy, which requires that all data be made available without restriction: http://journals.plos.org/plosbiology/s/data-availability. For more information, please also see this editorial: http://dx.doi.org/10.1371/journal.pbio.1001797
Note that we do not require all raw data. Rather, we ask that all individual quantitative observations that underlie the data summarized in the figures and results of your paper be made available in one of the following forms:

1) Supplementary files (e.g., excel). Please ensure that all data files are uploaded as 'Supporting Information' and are invariably referred to (in the manuscript, figure legends, and the Description field when uploading your files) using the following format verbatim: S1 Data, S2 Data, etc. Multiple panels of a single or even several figures can be included as multiple sheets in one excel file that is saved using exactly the following convention: S1_Data.xlsx (using an underscore)

2) Deposition in a publicly available repository. Please also provide the accession code or a reviewer link so that we may view your data before publication.

Regardless of the method selected, please ensure that you provide the individual numerical values that underlie the summary data displayed in the following figure panels as they are essential for readers to assess your analysis and to reproduce it: 1DF, 4C, S4 and S5.

All relevant data (including those to reproduce figure 1DF, 4C, S4, S5) are available at OSF. The link is provided in the data avaiability statement below and the data can be inspected.

"Data availability statement
All relevant code and data are available in an Open Science Framework (OSF) repository: https://doi.org/10.17605/OSF.IO/SNDPE"

NOTE: the numerical data provided should include all replicates AND the way in which the plotted mean and errors were derived (it should not present only the mean/average values). Please also ensure that figure legends in your manuscript include information on where the underlying data can be found, and ensure your supplemental data file/s has a legend.

This has been added for each data figures.

For example, Figure 1: “The underlying numerical data shown in B, D, F are available at https://doi.org/10.17605/OSF.IO/SNDPE”
Figure 2:

“The underlying numerical data are available at https://doi.org/10.17605/OSF.IO/SNDPE”

All supplemental figures and tables have a legend. (Our data are provided in OSF and therefore have no legend).

Please ensure that your Data Statement in the submission system accurately describes where your data can be found.

The information is stated in the data availability statement:
“**Data availability statement**
All relevant code and data are available in an Open Science Framework (OSF) repository: https://doi.org/10.17605/OSF.IO/SNDPE”

* CODE POLICY

Per journal policy, if you have generated any custom code during the course of this investigation, please make it available without restrictions. Please ensure that the code is sufficiently well documented and reusable, and that your Data Statement in the Editorial Manager submission system accurately describes where your code can be found. More information on our Code Policy, what and how to share can be found here: https://journals.plos.org/plosbiology/s/code-availability
Please note that we cannot accept sole deposition of code in GitHub, as this could be changed after publication. However, you can archive this version of your publicly available GitHub code to Zenodo. Once you do this, it will generate a DOI number, which you will need to provide in the Data Accessibility Statement (you are welcome to also provide the GitHub access information). See the process for doing this here: https://docs.github.com/en/repositories/archiving-a-github-repository/referencing-and-citing-content

Our code has been made available in the OSF repository, see data availability statement.

As you address these items, please take this last chance to review your reference list to ensure that it is complete and correct. If you have cited papers that have been retracted, please include the rationale for doing so in the manuscript text, or remove these references and replace them with relevant current references. Any changes to the reference list should be mentioned in the cover letter that accompanies your revised manuscript.

No changes to the reference list have been made.

Technical Requests
(Note that a response to all technical requests has also been copied and send per Email)

1) Thank you for stating "The study was approved by the local ethics committee of the University Hospital of the Goethe-University Frankfurt (number: 2021-509) in accordance with the Declaration of Helsinki.Prior to each session, participants provided written informed consent"
Please insert an Ethics Statement at the beginning of your Methods section, under a subheading 'Ethics Statement' and include the details.

It must include:
i) The name(s) of the Institutional Review Board(s) or Ethics Committee(s)
ii) The approval number(s), or a statement that approval was granted by the named board(s)
iii) A statement that formal consent was obtained (must state whether verbal/written) OR the reason consent was not obtained (e.g. anonymity). NOTE: If child participants, the statement must declare that formal consent was obtained from the parent/guardian.]

An "Ethics Statement" section has been added:
l. 597: "**Ethics Statement.** The study was approved by the local ethics committee of the University Hospital of the Goethe-University Frankfurt (Ethikkomission des Fachbereichs Medizin der Goethe-Universität, approval number: 2021-509) in accordance with the Declaration of Helsinki. Prior to each session, participants provided written informed consent."

2) We have noticed that you have uploaded (Supplementary Table 1 to Supplementary Table 26) as supporting information but you have not included them in the list of legends. Please add a citation of these Supplementary Tables into the list of legends of your supporting information files.

The Table citations have been added to the list of legends of the supporting information at the end of the main manuscript.

3) In your Data Availability Statement, please ensure you have provided all necessary access information in complete sentences. Access information should include the name of any data repositories used as well as digital object identifiers (DOIs), accession numbers or codes, or other persistent identifiers for all relevant data. If all data has been provided within your submission files, please use the statement "All relevant data is available within the manuscript and Supporting Information files." The Data Availability Statement will appear on the published version of your manuscript.
The data availability statement has been revised:
"**Data availability statement**
All relevant code and data are available in an Open Science Framework (OSF) repository: https://doi.org/10.17605/OSF.IO/SNDPE"

4) Please refer to the following article for PLOS Biology's best-practice recommendations for data presentation: https://journals.plos.org/plosbiology/article?id=10.1371/journal.pbio.1002128. Guidelines for statistical reporting, if relevant, may be found here: https://journals.plos.org/plosbiology/s/best-practices-in-research-reporting#loc-statistical-reporting.

Our study meets these recommendations.

5) Some material included in your submission may be copyrighted. According to PLOS's copyright policy, authors who use figures or other material (e.g., graphics, clipart, maps) from another author or copyright holder must demonstrate or obtain permission to publish this material under the Creative Commons Attribution 4.0 International (CC BY 4.0) License used by PLOS journals. Please closely review the details of PLOS's copyright requirements here: PLOS Licenses and Copyright. If you need to request permissions from a copyright holder, you may use PLOS's Copyright Content Permission form.
Please respond directly to this email and provide any known details concerning your material's license terms and permissions required for reuse, even if you have not yet obtained copyright permissions or are unsure of your material's copyright compatibility. Once you have responded and addressed all other outstanding technical requirements, you may resubmit your manuscript within

Editorial Manager.
Potential Copyright Issues:
[Figure 1 (Hand icon, Headphone icon), Figure 3 (Headphone icon), Figure7 (Lip,ear icon),Supplementary Figure 7]: Please confirm whether you drew the images / clip-art within the figure panels by hand. If you did not draw the images, please provide (a) a link to the source of the images or icons and their license / terms of use; or (b) written permission from the copyright holder to publish the images or icons under our CC BY 4.0 license. Alternatively, you may replace the images with open source alternatives. See these open source resources you may use to replace images / clip-art:
- https://commons.wikimedia.org
- https://openclipart.org/

The only icon that was drawn is the "spectral profile" icon in Figure 7. All other icons are taken from *The Noun Project* under the Creative Commons Attribution 3.0 license and are cited in the legend of each figure. We revised the legends, as the citation of one icon (the "Ear") was missing, and the link to the license was missing.

Figure 1 legend:
"All icons, the "Headphones" by Dong Gyu Yang, "speaking" by Ainul Muttaqin and the "Click" by Wahyu Prihantoro, are taken from The Noun Project used under the Creative Commons Attribution 3.0 license https://creativecommons.org/licenses/by/3.0/."

(Note that the hand icon is called "Click". Furthermore, we realized that one of the hand icons was still an old icon, that we now replaced with the icon "Click". The revised figure 1 is submitted)

Figure 3 legend:
"The brain plot was generated using the Fieldtrip toolbox and plotting the template brain (ROI_MNI_V4.nii using: ft_readatlas) that had been interpolated to a brain surface mesh template (surface_pial_both.mat; using ft_sourceinterpolate) and plotted (using: ft_sourceplot), the ROI has been drawn. All other icons, the "Headphones" by Dong Gyu Yang, "speaking" by Ainul Muttaqin, are taken from The Noun Project used under the Creative Commons Attribution 3.0 license https://creativecommons.org/licenses/by/3.0/. The underlying numerical data (i.e. in B, D, E) are available at https://doi.org/10.17605/OSF.IO/SNDPE"

Figure 7 legend:
"The "mouth" icon by little_dipper_studio, the "Ear" icon by Gregor Cresnar, the "Brain" icon by Lewen Design and the "speaking" icon by Ainul Muttaqin are taken from The Noun Project used under the Creative Commons Attribution 3.0 license https://creativecommons.org/licenses/by/3.0/."

Supplementary Figure 7 legend:
"The underlying statistic data, displayed in this figure are available in Supplementary Table 26. The brain plots were generated using the Fieldtrip toolbox by mapping the R-values onto the respective ROIs of the AAL atlas template brain (ROI_MNI_V4.nii using: ft_readatlas) that had been interpolated to a brain surface mesh template (surface_pial_both.mat; using ft_sourceinterpolate) and plotted (using: ft_sourceplot)."
