# Supplementary material for: Endogenous auditory and motor brain rhythms predict individual speech tracking
Source: PLoS Biol. 2026 Jul 31;24(7):e3003924. doi: 10.1371/journal.pbio.3003924 (PMC13446743; doi:10.1371/journal.pbio.3003924)
Supplement: Supplementary file 37 [file pbio.3003924.s036.docx]

We sincerely thank the Editors and Reviewers for their time and effort in providing insightful comments and suggestions that helped improve our manuscript.

In summary, we now revised the manuscript to tone down our claims about the two-route interpretation and the non-significant behavior-neural analysis. We revised Fig. 7 to improve its clarity. Furthermore, following the request by Reviewer 1 we added an exploratory analysis. We correlated the frequency of the spectral profile theta peaks of a larger set of brain areas (15 frontal, central, temporal and parietal ROIs, selected by the presence of theta clusters and their relevance to speech processing) with the speech tracking in STG, HG, as well as the behavioral performance. The analyses support our main findings and are added (tables and a brain map figure) to the supplementary material.

**Reviewer #1**

Reviewer #1 (Sander van Bree signed his report): The revised manuscript is improved. First, I think the core result is generally on firm grounds: endogenous auditory and motor frequencies predict speech tracking, and motor-related effects appear more strongly in high synchronizers. I have remaining concerns, but my general impression is that the manuscript is now in a good state.

I thank the authors for addressing the participant-exclusion issue/clarification, adding some theoretical context, streamlining the terminology, and taking on board a few other suggestions I made.

My main concern is and was that the interpretation of the two pathways remains speculative, not only because of the correlational nature of the work, but also because some effects one might expect if that interpretation is correct are not present in a convincing form. Most notably, the revised analyses still do not provide compelling evidence for a relationship between the auditory-motor neural estimates and speech comprehension; the remaining signals appear to be exploratory and uncorrected. So I still think the proposed two-route account feels a bit ahead of the evidence the speech-tracking results are stronger than the larger comprehension story. Yet at the same time I think this work is a valuable contribution to the field.

Thank you for the overall positive evaluation of our revised manuscript. We believe that our findings provide initial evidence for differential processing routes, with some individuals relying more on motor processing. However, we agree that the “two-route” speculation (particularly the working memory part) requires future research. We now revised the discussion of the two-route interpretation to point out that this is a speculation and instead focused more on the effects of the endogenous brain rhythms as the main result of our study.

In the abstract:

l. 14: “The findings highlight differential speech processing preferences across individuals, with an auditory–motor route, related to enhanced comprehension performance.”

In the introduction:

l. 109: “Our study provides evidence for a neural oscillatory account that highlights alternative speech processing routes across individuals.”

In the discussion section:

l. 420: “In contrast, low auditory-motor synchronizers rely more on auditory oscillatory populations and possibly may more heavily recruit working memory processes, as should be further explored by future research.”

l. 516: “This may be an explanation for this finding, leading to the speculation of different speech processing routes, with high synchronizers relying more on predictions from the motor system, related to enhanced comprehension performance, and low synchronizers relying more on auditory processing and an auditory working-memory contribution.”

I understand that a broader brain-wide behavior-brain analysis was not feasible, it is unfortunate because many researchers may read this study because they want to consult what correlations exist, they might not be interested in this specific story -- but I understand the practical limitation. However, there are less computationally intensive plots that would be useful, without LMM. For example, a map of Pearson correlations between theta frequency (spectral fingerprint peak) and speech tracking, or between theta frequency and comprehension.

Following the reviewer’s suggestions, we now extracted from the resting state data the theta frequency peaks of various frontal, central, temporal and parietal brain regions.

Regions of interest (ROIs) for this analysis were selected based on the following reasoning: Keitel and Gross (2016, PLOS Biol) report a distribution of peak frequencies of the brain rhythms with most power across the cortex (Suppl. figure S12). They show that theta is particularly present in superior temporal and prefrontal cortex. Therefore, we pre-selected all brain areas from the frontal cortex and temporal cortex and choose those areas that consistently showed theta cluster across the studies Keitel & Gross (2016, PLOS Biol) and Lubinus et al. (2021, Cereb Cortex). Additionally, we selected central brain areas and two parietal areas with consistent theta clusters across these studies, because these areas are relevant to speech-motor planning (Supramarginal Gyrus and Inferior Parietal cortex). If, for these brain areas, in our data a theta cluster was present in a larger number of participants the area was chosen as ROI for the correlational analysis. We then used Spearman rank correlation to correlate all these brain rhythms (averaged across hemispheres) with the speech tracking in STG and HG and with the performance in the intelligibility task, separately. FDR correction was used within each of the analyses. A supplementary figure S7 and the corresponding tables (Supplementary tables 21-22) were added to the supplement. The analyses show correlations of tracking in STG with the endogenous peak frequencies of SMA, STG and IFG (orbital part), whereas only the correlation with the peak frequency of the endogenous STG brain rhythm survived multiple comparison control. The effects and direction of effects are consistent with our main results. Correlations with the behavioral performance were present for peak frequencies in STG and IFG, but those did not survive multiple comparison control (Supplementary table 23). Additionally, we added tables where left and right hemispheres were correlated separately, just for readers with a broader interest. The effects are consistent with the results from the data that were averaged across the hemispheres (Supplementary tables 24-26).

In the manuscript:

L. 464: “[…] (see also: Supplementary Fig 7, Tables 22-26).”

In the supplementary material:

**Supplementary Figure 7. Correlations between the theta brain rhythm of various regions of interest and speech tracking in STG and HG and performance in the intelligibility task.** We pre-selected all brain areas from the frontal, central and temporal cortex and choose those areas that consistently showed theta cluster across the studies Keitel & Gross (2016) and Lubinus et al. (2021). Additionally, two parietal areas were chosen. If for these brain areas a theta cluster was present in a larger number of participants, the area was chosen as region of interest (ROI) for the correlational analysis. The following 15 ROIs from the AAL atlas were analyzed: Frontal_Inf_Tri_, Supp_Motor_Area_, Heschl_, Temporal_Sup_, Parietal_Inf_, Precentral_, Insula_, Rolandic_Oper_, Supra_Marginal_Gyrus_, Frontal_Inf_Oper_, Frontal_Sup_Medial_, Frontal_Mid_, Frontal_Sup_, Frontal_Inf_Orb_, Cingulum_Mid_. Peak frequencies of the theta cluster with maximum amplitude were selected for each participant. Peak frequencies were averaged across the left and right hemisphere. Spearman correlations between the peak frequencies of the endogenous brain rhythms in each ROI and the speech tracking in STG (upper row; averaged across hemispheres and syllabic rate conditions), speech tracking in HG (middle row; averaged across hemispheres and syllabic rate conditions) and performance (bottom row) were computed. False-discovery (FDR) control for multiple comparisons was applied. R-values are color coded. Although, several ROIs show significant effects, only the negative correlations of STG peak frequencies with STG speech tracking survive multiple comparison control.

**Supplementary Table 21. Spearman rank correlations between spectral peak measures in various ROIs and speech tracking in Superior Temporal Gyrus (STG).** Peak frequencies of the theta cluster of endogenous brain rhythms with maximum amplitude were selected for each participant. Peak frequencies were averaged across the left and right hemisphere. Spearman correlations between the peak frequencies in each region of interest (ROI, AAL labels are displayed) and the speech tracking in STG (averaged across hemispheres and syllabic rate conditions) were computed. Correlation coefficients (Spearman’s ρ, *rho*) and corresponding two-tailed p-values are reported. P-values are additionally reported corrected for multiple comparisons using the false discovery rate (FDR) procedure (p_FDR_). Significance is indicated by asterix (* < .05; ** < .01).

| ROI | **rho** | **p** | **p_FDR_** |
| --- | --- | --- | --- |
| Frontal_Inf_Tri_ | -0.0079033 | 0.95347203 | 0.95347203 |
| Supp_Motor_Area_ | 0.31069408 | 0.01865964 * | 0.1399473 |
| Heschl_ | -0.1459404 | 0.27870744 | 0.61447408 |
| Temporal_Sup_ | -0.3857494 | 0.00304215 ** | 0.04563231 * |
| Parietal_Inf_ | -0.0872407 | 0.51873752 | 0.78573122 |
| Precentral_ | -0.1520786 | 0.25876514 | 0.61447408 |
| Insula_ | 0.06824721 | 0.6139602 | 0.78573122 |
| Rolandic_Oper_ | -0.0697992 | 0.60590505 | 0.78573122 |
| Supra_Marginal_Gyrus_ | 0.03996368 | 0.76787728 | 0.82272566 |
| Frontal_Inf_Oper_ | 0.06545149 | 0.62858498 | 0.78573122 |
| Frontal_Sup_Medial_ | 0.20077209 | 0.13426087 | 0.50347825 |
| Frontal_Mid_ | 0.14354612 | 0.28675457 | 0.61447408 |
| Frontal_Sup_ | -0.1094411 | 0.41771332 | 0.78321248 |
| Frontal_Inf_Orb_ | 0.28079285 | 0.03436671 * | 0.17183354 |
| Cingulum_Mid_ | -0.0412608 | 0.7605635 | 0.82272566 |

**Supplementary Table 22. Spearman rank correlations between spectral peak measures in various regions of interest (ROIs) and speech tracking in Heschl’s Gyrus (HG).** Peak frequencies of the theta cluster of endogenous brain rhythms with maximum amplitude were selected for each participant. Peak frequencies were averaged across the left and right hemisphere. Spearman correlations between the peak frequencies in each region of interest (ROI, AAL labels are displayed) and the speech tracking in HG (averaged across hemispheres and syllabic rate conditions) were computed. Correlation coefficients (Spearman’s ρ, *rho*) and corresponding two-tailed p-values are reported. P-values are additionally reported corrected for multiple comparisons using the false discovery rate (FDR) procedure (p_fdr). Significance is indicated by asterix (* < .05; ** < .01).

| ROI | **rho** | **p** | **p_FDR_** |
| --- | --- | --- | --- |
| Frontal_Inf_Tri_ | 0.01349734 | 0.92062257 | 0.9483241 |
| Supp_Motor_Area_ | 0.22111404 | 0.09834719 | 0.4003309 |
| Heschl_ | -0.0937815 | 0.48775722 | 0.73163583 |
| Temporal_Sup_ | -0.2492304 | 0.06154189 | 0.4003309 |
| Parietal_Inf_ | -0.140549 | 0.29704076 | 0.55695143 |
| Precentral_ | -0.2462069 | 0.06486727 | 0.4003309 |
| Insula_ | 0.00877882 | 0.9483241 | 0.9483241 |
| Rolandic_Oper_ | -0.0690504 | 0.60978563 | 0.83152586 |
| Supra_Marginal_Gyrus_ | 0.03170996 | 0.81486088 | 0.94022409 |
| Frontal_Inf_Oper_ | 0.11117653 | 0.41032296 | 0.68387159 |
| Frontal_Sup_Medial_ | 0.21589586 | 0.10675491 | 0.4003309 |
| Frontal_Mid_ | 0.16560511 | 0.21828054 | 0.54570134 |
| Frontal_Sup_ | -0.1458671 | 0.27895164 | 0.55695143 |
| Frontal_Inf_Orb_ | 0.16781824 | 0.21210372 | 0.54570134 |
| Cingulum_Mid_ | -0.0473809 | 0.72634672 | 0.9079334 |

**Supplementary Table 23. Spearman rank correlations between spectral peak measures in various regions of interest (ROIs) and speech comprehension performance.** Peak frequencies of the theta cluster of endogenous brain rhythms with maximum amplitude were selected for each participant. Peak frequencies were averaged across the left and right hemisphere. Spearman correlations between the peak frequencies in each region of interest (ROI, AAL labels are displayed) and the performance in the speech intelligibility task (averaged across syllabic rate conditions) were computed. Correlation coefficients (Spearman’s ρ, *rho*) and corresponding two-tailed p-values are reported. P-values are additionally reported corrected for multiple comparisons using the false discovery rate (FDR) procedure (p_fdr). Significance is indicated by asterix (* < .05; ** < .01).

| ROI | **rho** | **p** | **p_FDR_** |
| --- | --- | --- | --- |
| Frontal_Inf_Tri_ | -0.0655353 | -0.0655353 * | 0.9483241 |
| Supp_Motor_Area_ | -0.2583413 | -0.2583413 | 0.4003309 |
| Heschl_ | -0.1917058 | -0.1917058 | 0.73163583 |
| Temporal_Sup_ | 0.02165519 | 0.02165519 * | 0.4003309 |
| Parietal_Inf_ | -0.0600818 | -0.0600818 | 0.55695143 |
| Precentral_ | -0.0937698 | -0.0937698 | 0.4003309 |
| Insula_ | 0.06619882 | 0.06619882 | 0.9483241 |
| Rolandic_Oper_ | -0.2041887 | -0.2041887 | 0.83152586 |
| Supra_Marginal_Gyrus_ | -0.1104629 | -0.1104629 | 0.94022409 |
| Frontal_Inf_Oper_ | 0.03500062 | 0.03500062 | 0.68387159 |
| Frontal_Sup_Medial_ | -0.2734312 | -0.2734312 | 0.4003309 |
| Frontal_Mid_ | -0.173316 | -0.173316 | 0.54570134 |
| Frontal_Sup_ | -0.272233 | -0.272233 | 0.55695143 |
| Frontal_Inf_Orb_ | -0.0638269 | -0.0638269 | 0.54570134 |
| Cingulum_Mid_ | -0.257469 | -0.257469 | 0.9079334 |

The new Figure 7 is a useful addition. It helps by making the scope of the paper more explicit and by summarizing the main idea of the results. However, I find the execution not quite optimal. The colour scheme is difficult to follow because the same colours seem to carry different meanings across the behavioural and neural halves of the figure. In the neural panel, colours appear to map onto regions or neural variables, whereas in the behavioural panel the same colours refer to different things (e.g., red refers to motor production behaviour on the left, but red also refers to SMA neural frequency on the right). The neural panel is split into a low and high half, which could be misinterpreted as low vs high frequencies clustering to different parts of the brain. It also contains many details. I would redesign under the question: what is the essential information, and what is the most straightforward way to communicate it?

We agree that the color scheme was confusing. We have now removed the color scheme from the behavioral data and focused on the neural data for the auditory/motor distinction. Additionally, we adjusted the labels high and low motor synchronizers to clarify that this is not referring different parts of the brain (the spectral profiles and brain symbols are now above the high/low synchronizer labels to avoid confusion).

**Figure 7** **Schematic summary of the results.** The left part of the figure illustrates the effects of the behavioral auditory-motor parameters on speech comprehension (black speaker icon). Individuals with high auditory-motor synchronization (Highs) show effects of the spontaneous speech production rate (arrow, mouth icon) on speech comprehension and effects of the preferred auditory rate (arrow, ear icon). In contrast, low auditory-motor synchronizers (Lows) show effects of working memory (arrow, WM icon). The direction of the effects is indicated (+/- ). The right part of the figure illustrates the effects of the neural auditory-motor parameters on speech comprehension. In Highs, speech tracking in HG was predicted by the frequency of the endogenous speech-motor rhythm in IFG (pink arrow, pink region of interest, ROI), with lower rates related to higher tracking. Speech tracking in STG was predicted by the frequency of the endogenous speech-motor rhythm in SMA (red arrow/ROI), with higher frequencies related to higher tracking. In the Lows, higher tracking in STG was predicted by lower frequencies of the endogenous auditory brain rhythm in STG (blue arrow/ROI). The behavioral results parallel the neural results with effects of the spontaneous production rate and the endogenous motor brain rhythms only observed in high auditory-motor synchronizers. In an exploratory analysis (with no control for multiple comparisons), higher speech comprehension was predicted by lower tracking in HG (transparent arrow), the findings however require further research. The “mouth” icon by little_dipper_studio, the “Brain” icon by Lewen Design and the “speaking” icon by Ainul Muttaqin were used under the Creative Commons Attribution 3.0 license via The Noun Project. The “Brain” icon by Lewen Design was used under the Creative Commons Attribution 3.0 license via The Noun Project.

**Reviewer #3**

Reviewer #3: I thank the authors for clarifying several points, especially with regards to the signal processing. My comments there have been addressed.

I appreciate the efforts at tempering the language around the insignificant results, but in my opinion they do not go far enough. The new text states that the exploratory analysis "reveals" or "suggests" relationships at several points throughout. I do not think uncontrolled p=0.04 does either of those and would be much more cautious about making these claims (even 0.04 with MC corrections is borderline in my view). If you still want to talk about it, it might be more appropriate to say that exploratory analysis was not significant and provided no evidence of such a relationship, but indicated it as a potential topic for future study.

As this is a stylistic issue, I leave it to the editor to decide how they wish to handle it.

Thank you for the positive evaluation of our revised manuscript. We have further revised the language in the manuscript to remove claims of significance with regard to the exploratory analysis and clarifying the interpretation of the exploratory analysis.

Abstract, l. 11:

“These findings align with participants’ behavioral data and provide support for oscillatory accounts of auditory–motor interactions during speech perception.”

In the results section:

l. 386: “Note that given that no FDR correction was applied, these findings are not considered significant, however, future research may further investigate the nature of the relationship.”

In the discussion section:

l. 417: “An exploratory analysis, however, indicated the relationship as a potential topic for future research.”

l. 453: “Although comprehension and neural tracking both declined at higher rates, we find no significant correlation between them.”

l. 542: “Neurophysiological findings parallel the behavioral findings”

l. 553: “We found no direct relationship between the auditory-motor neural estimates and speech comprehension. Although, an exploratory analysis hints at a possible relationship. Lower speech tracking in Heschl’s Gyrus may be related to higher performance in the intelligibility task, and higher auditory-motor coupling between SMA and Heschl’s Gyrus and SMA and STG to higher comprehension. These findings were not significant, however, may inspire future research. Speech comprehension is intricate and shaped by numerous interacting variables.”

We furthermore removed the exploratory findings from figure 7.
