# Supplementary material for: Endogenous auditory and motor brain rhythms predict individual speech tracking
Source: PLoS Biol. 2026 Jul 31;24(7):e3003924. doi: 10.1371/journal.pbio.3003924 (PMC13446743; doi:10.1371/journal.pbio.3003924)
Supplement: Peer review history

# Author comment: Author response to Decision Letter 0

**DOI:** 10.1371/journal.pbio.3003924.r001

26 Feb 2026

Attachment Submitted filename: PLOSBiol_Response_letter_revision.pdf

# Editor report: Decision Letter 0

**DOI:** 10.1371/journal.pbio.3003924.r002

**Authors:** Christian Schnell, PhD

10 Mar 2026

Dear Johanna,

Thank you for submitting your revised manuscript entitled "Endogenous auditory and motor brain rhythms predict individual speech tracking" for consideration as a Research Article by PLOS Biology. Please allow me first to apologize for the long delay in getting back to you. The discussions with the academic editor took a bit longer for the reasons outlined below.

Your manuscript has now been evaluated by the PLOS Biology editorial staff as well as by an academic editor with relevant expertise and I am writing to let you know that we would like to send your submission back for external peer review, despite some concerns whether the reviewers' concerns have been fully addressed, in particular those about novelty and whether there is now sufficient experimental evidence for your claims.

However, before we can send your manuscript to reviewers, we need you to complete your submission by providing the metadata that is required for full assessment. To this end, please login to Editorial Manager where you will find the paper in the 'Submissions Needing Revisions' folder on your homepage. Please click 'Revise Submission' from the Action Links and complete all additional questions in the submission questionnaire.

Once your full submission is complete, your paper will undergo a series of checks in preparation for peer review. After your manuscript has passed the checks it will be sent out for review. To provide the metadata for your submission, please Login to Editorial Manager (https://www.editorialmanager.com/pbiology) within two working days, i.e. by Mar 12 2026 11:59PM.

During the process of completing your manuscript submission, you will be invited to opt-in to posting your pre-review manuscript as a bioRxiv preprint. Visit http://journals.plos.org/plosbiology/s/preprints for full details. If you consent to posting your current manuscript as a preprint, please upload a single Preprint PDF.

Feel free to email us at plosbiology@plos.org if you have any queries relating to your submission.

Kind regards,

Christian

Christian Schnell, PhD

Senior Editor

PLOS Biology

cschnell@plos.org

# Author comment: Author response to Decision Letter 1

**DOI:** 10.1371/journal.pbio.3003924.r003

11 Mar 2026

Attachment Submitted filename: PLOSBiol_Response_letter_revision_auresp_1.pdf

# Peer review: Decision Letter 1

**DOI:** 10.1371/journal.pbio.3003924.r004

**Authors:** Christian Schnell, PhD

10 Apr 2026

Dear Johanna,

Thank you for your patience while we considered your revised manuscript "Endogenous auditory and motor brain rhythms predict individual speech tracking" for consideration as a Research Article at PLOS Biology. Your revised study has now been evaluated by the PLOS Biology editors, the Academic Editor and two of the original reviewers

In light of the reviews, which you will find at the end of this email, we are pleased to offer you the opportunity to fully address the remaining points from the reviewers (including tempering the language around the insignificant results) in a revision that we anticipate should not take you very long. We will then assess your revised manuscript and your response to the reviewers' comments with our Academic Editor aiming to avoid further rounds of peer-review, although we might need to consult with the reviewers, depending on the nature of the revisions.

In addition to these revisions, you may need to complete some formatting changes, which you will receive in a follow up email. A member of our team will be in touch with a set of requests shortly. If you do not receive a separate email within a few days, please assume that checks have been completed, and no additional changes are required.

We expect to receive your revised manuscript within 1 month. Please email us (plosbiology@plos.org) if you have any questions or concerns, or would like to request an extension.

At this stage, your manuscript remains formally under active consideration at our journal; please notify us by email if you do not intend to submit a revision so that we withdraw the manuscript.

**IMPORTANT - SUBMITTING YOUR REVISION**

Your revisions should address the specific points made by each reviewer. Please submit the following files along with your revised manuscript:

1. A 'Response to Reviewers' file - this should detail your responses to the editorial requests, present a point-by-point response to all of the reviewers' comments, and indicate the changes made to the manuscript.

*NOTE: In your point-by-point response to the reviewers, please provide the full context of each review. Do not selectively quote paragraphs or sentences to reply to. The entire set of reviewer comments should be present in full and each specific point should be responded to individually.

You should also cite any additional relevant literature that has been published since the original submission and mention any additional citations in your response.

2. In addition to a clean copy of the manuscript, please also upload a 'track-changes' version of your manuscript that specifies the edits made. This should be uploaded as a "Revised Article with Changes Highlighted " file type.

*Resubmission Checklist*

When you are ready to resubmit your revised manuscript, please refer to this resubmission checklist: https://plos.io/Biology_Checklist

To submit a revised version of your manuscript, please go to https://www.editorialmanager.com/pbiology/ and log in as an Author. Click the link labelled 'Submissions Needing Revision' where you will find your submission record.

Please make sure to read the following important policies and guidelines while preparing your revision:

*Published Peer Review*

Please note while forming your response, if your article is accepted, you may have the opportunity to make the peer review history publicly available. The record will include editor decision letters (with reviews) and your responses to reviewer comments. If eligible, we will contact you to opt in or out. Please see here for more details:

https://blogs.plos.org/plos/2019/05/plos-journals-now-open-for-published-peer-review/

*PLOS Data Policy*

Please note that as a condition of publication PLOS' data policy (http://journals.plos.org/plosbiology/s/data-availability) requires that you make available all data used to draw the conclusions arrived at in your manuscript. If you have not already done so, you must include any data used in your manuscript either in appropriate repositories, within the body of the manuscript, or as supporting information (N.B. this includes any numerical values that were used to generate graphs, histograms etc.). For an example see here: http://www.plosbiology.org/article/info%3Adoi%2F10.1371%2Fjournal.pbio.1001908#s5

*Blot and Gel Data Policy*

We require the original, uncropped and minimally adjusted images supporting all blot and gel results reported in an article's figures or Supporting Information files. We will require these files before a manuscript can be accepted so please prepare them now, if you have not already uploaded them. Please carefully read our guidelines for how to prepare and upload this data: https://journals.plos.org/plosbiology/s/figures#loc-blot-and-gel-reporting-requirements

*Protocols deposition*

To enhance the reproducibility of your results, we recommend that if applicable you deposit your laboratory protocols in protocols.io, where a protocol can be assigned its own identifier (DOI) such that it can be cited independently in the future. Additionally, PLOS ONE offers an option for publishing peer-reviewed Lab Protocol articles, which describe protocols hosted on protocols.io. Read more information on sharing protocols at https://plos.org/protocols?utm_medium=editorial-email&utm_source=authorletters&utm_campaign=protocols

Thank you again for your submission to our journal. We hope that our editorial process has been constructive thus far, and we welcome your feedback at any time. Please don't hesitate to contact us if you have any questions or comments.

Sincerely,

Christian

Christian Schnell, PhD

Senior Editor

PLOS Biology

cschnell@plos.org

----------------------------------------------------------------

REVIEWS:

Reviewer #1 (Sander van Bree signed his report): The revised manuscript is improved. First, I think the core result is generally on firm grounds: endogenous auditory and motor frequencies predict speech tracking, and motor-related effects appear more strongly in high synchronizers. I have remaining concerns, but my general impression is that the manuscript is now in a good state.

I thank the authors for addressing the participant-exclusion issue/clarification, adding some theoretical context, streamlining the terminology, and taking on board a few other suggestions I made.

My main concern is and was that the interpretation of the two pathways remains speculative, not only because of the correlational nature of the work, but also because some effects one might expect if that interpretation is correct are not present in a convincing form. Most notably, the revised analyses still do not provide compelling evidence for a relationship between the auditory-motor neural estimates and speech comprehension; the remaining signals appear to be exploratory and uncorrected. So I still think the proposed two-route account feels a bit ahead of the evidence the speech-tracking results are stronger than the larger comprehension story. Yet at the same time I think this work is a valuable contribution to the field.

I understand that a broader brain-wide behavior-brain analysis was not feasible, it is unfortunate because many researchers may read this study because they want to consult what correlations exist, they might not be interested in this specific story -- but I understand the practical limitation. However, there are less computationally intensive plots that would be useful, without LMM. For example, a map of Pearson correlations between theta frequency (spectral fingerprint peak) and speech tracking, or between theta frequency and comprehension.

The new Figure 7 is a useful addition. It helps by making the scope of the paper more explicit and by summarizing the main idea of the results. However, I find the execution not quite optimal. The colour scheme is difficult to follow because the same colours seem to carry different meanings across the behavioural and neural halves of the figure. In the neural panel, colours appear to map onto regions or neural variables, whereas in the behavioural panel the same colours refer to different things (e.g., red refers to motor production behaviour on the left, but red also refers to SMA neural frequency on the right). The neural panel is split into a low and high half, which could be misinterpreted as low vs high frequencies clustering to different parts of the brain. It also contains many details. I would redesign under the question: what is the essential information, and what is the most straightforward way to communicate it?

Reviewer #3: I thank the authors for clarifying several points, especially with regards to the signal processing. My comments there have been addressed.

I appreciate the efforts at tempering the language around the insignificant results, but in my opinion they do not go far enough. The new text states that the exploratory analysis "reveals" or "suggests" relationships at several points throughout. I do not think uncontrolled p=0.04 does either of those and would be much more cautious about making these claims (even 0.04 with MC corrections is borderline in my view). If you still want to talk about it, it might be more appropriate to say that exploratory analysis was not significant and provided no evidence of such a relationship, but indicated it as a potential topic for future study.

As this is a stylistic issue, I leave it to the editor to decide how they wish to handle it.

# Author comment: Author response to Decision Letter 2

**DOI:** 10.1371/journal.pbio.3003924.r005

11 May 2026

Attachment Submitted filename: response_letter_revision_2.docx

# Editor report: Decision Letter 2

**DOI:** 10.1371/journal.pbio.3003924.r006

**Authors:** Christian Schnell, PhD

29 May 2026

Dear Johanna,

Thank you for your patience while we considered your revised manuscript "Endogenous auditory and motor brain rhythms predict individual speech tracking" for publication as a Research Article at PLOS Biology. This revised version of your manuscript has been evaluated by the PLOS Biology editors and the Academic Editor.

Based on our Academic Editor's assessment of your revision, we are likely to accept this manuscript for publication, provided you implement a few textual revisions and address the following data and other policy-related requests:

* The sentences starting from line 11 in the abstract need to be revised, because the current experimental evidence does not support the claims that "these findings align with participants’ behavioral data...". Please make it clear to the reader that, based on your statistical analysis, there is no detectable relationship between the neural data and behavioral readouts.

* The same for lines 96-100 in the introduction.

* The sentence in lines 107-109 ("Although we found no evidence...") could simply be removed from here. This is also true for the sentences in lines 418-422.

* Please also rephrase the sentences in lines 566 to 570.

* Please add the links to the funding agencies in the Financial Disclosure statement in the manuscript details.

* Please mention in the competing interest statements that David Poeppel is a member of PLOS Biology's editorial board and that Johanna Rimmele has done freelance work and acted as a Guest Editor for PLOS Biology.

* DATA POLICY:

You may be aware of the PLOS Data Policy, which requires that all data be made available without restriction: http://journals.plos.org/plosbiology/s/data-availability. For more information, please also see this editorial: http://dx.doi.org/10.1371/journal.pbio.1001797

Note that we do not require all raw data. Rather, we ask that all individual quantitative observations that underlie the data summarized in the figures and results of your paper be made available in one of the following forms:

1) Supplementary files (e.g., excel). Please ensure that all data files are uploaded as 'Supporting Information' and are invariably referred to (in the manuscript, figure legends, and the Description field when uploading your files) using the following format verbatim: S1 Data, S2 Data, etc. Multiple panels of a single or even several figures can be included as multiple sheets in one excel file that is saved using exactly the following convention: S1_Data.xlsx (using an underscore).

2) Deposition in a publicly available repository. Please also provide the accession code or a reviewer link so that we may view your data before publication.

Regardless of the method selected, please ensure that you provide the individual numerical values that underlie the summary data displayed in the following figure panels as they are essential for readers to assess your analysis and to reproduce it: 1DF, 4C, S4 and S5.

NOTE: the numerical data provided should include all replicates AND the way in which the plotted mean and errors were derived (it should not present only the mean/average values).

Please also ensure that figure legends in your manuscript include information on where the underlying data can be found, and ensure your supplemental data file/s has a legend.

Please ensure that your Data Statement in the submission system accurately describes where your data can be found.

* CODE POLICY

Per journal policy, if you have generated any custom code during the course of this investigation, please make it available without restrictions. Please ensure that the code is sufficiently well documented and reusable, and that your Data Statement in the Editorial Manager submission system accurately describes where your code can be found. More information on our Code Policy, what and how to share can be found here: https://journals.plos.org/plosbiology/s/code-availability

Please note that we cannot accept sole deposition of code in GitHub, as this could be changed after publication. However, you can archive this version of your publicly available GitHub code to Zenodo. Once you do this, it will generate a DOI number, which you will need to provide in the Data Accessibility Statement (you are welcome to also provide the GitHub access information). See the process for doing this here: https://docs.github.com/en/repositories/archiving-a-github-repository/referencing-and-citing-content

As you address these items, please take this last chance to review your reference list to ensure that it is complete and correct. If you have cited papers that have been retracted, please include the rationale for doing so in the manuscript text, or remove these references and replace them with relevant current references. Any changes to the reference list should be mentioned in the cover letter that accompanies your revised manuscript.

In addition to these revisions, you may need to complete some formatting changes, which you will receive in a follow up email. A member of our team will be in touch with a set of requests shortly. If you do not receive a separate email within a few days, please assume that checks have been completed, and no additional changes are required.

We expect to receive your revised manuscript within two weeks.

To submit your revision, please go to https://www.editorialmanager.com/pbiology/ and log in as an Author. Click the link labelled 'Submissions Needing Revision' to find your submission record. Your revised submission must include the following:

- a cover letter that should detail your responses to any editorial requests, if applicable, and whether changes have been made to the reference list

- a Response to Reviewers file that provides a detailed response to the reviewers' comments (if applicable, if not applicable please do not delete your existing 'Response to Reviewers' file.)

- a track-changes file indicating any changes that you have made to the manuscript.

NOTE: If Supporting Information files are included with your article, note that these are not copyedited and will be published as they are submitted. Please ensure that these files are legible and of high quality (at least 300 dpi) in an easily accessible file format. For this reason, please be aware that any references listed in an SI file will not be indexed. For more information, see our Supporting Information guidelines:

https://journals.plos.org/plosbiology/s/supporting-information

*Published Peer Review History*

Please note that you may have the opportunity to make the peer review history publicly available. The record will include editor decision letters (with reviews) and your responses to reviewer comments. If eligible, we will contact you to opt in or out. Please see here for more details:

https://plos.org/published-peer-review-history/

*Press*

Should you, your institution's press office or the journal office choose to press release your paper, please ensure you have opted out of Early Article Posting on the submission form. We ask that you notify us as soon as possible if you or your institution is planning to press release the article.

*Protocols deposition*

To enhance the reproducibility of your results, we recommend that if applicable you deposit your laboratory protocols in protocols.io, where a protocol can be assigned its own identifier (DOI) such that it can be cited independently in the future. Additionally, PLOS ONE offers an option for publishing peer-reviewed Lab Protocol articles, which describe protocols hosted on protocols.io. Read more information on sharing protocols at https://plos.org/protocols?utm_medium=editorial-email&utm_source=authorletters&utm_campaign=protocols

Please do not hesitate to contact me should you have any questions.

Sincerely,

Christian

Christian Schnell, PhD

Senior Editor

cschnell@plos.org

PLOS Biology

# Author comment: Author response to Decision Letter 3

**DOI:** 10.1371/journal.pbio.3003924.r007

20 Jun 2026

Attachment Submitted filename: response_letter_revision3.pdf

# Editor report: Decision Letter 3

**DOI:** 10.1371/journal.pbio.3003924.r008

**Authors:** Christian Schnell, PhD

14 Jul 2026

Dear Johanna,

Thank you for the submission of your revised Research Article "Endogenous auditory and motor brain rhythms predict individual speech tracking" for publication in PLOS Biology. On behalf of my colleagues and the Academic Editor, Andrea Martin, I am pleased to say that we can in principle accept your manuscript for publication, provided you address any remaining formatting and reporting issues. These will be detailed in an email you should receive within 2-3 business days from our colleagues in the journal operations team; no action is required from you until then. Please note that we will not be able to formally accept your manuscript and schedule it for publication until you have completed any requested changes.

Please take a minute to log into Editorial Manager at http://www.editorialmanager.com/pbiology/, click the "Update My Information" link at the top of the page, and update your user information to ensure an efficient production process.

PRESS

We frequently collaborate with press offices. If your institution or institutions have a press office, please notify them about your upcoming paper at this point, to enable them to help maximize its impact. If the press office is planning to promote your findings, we would be grateful if they could coordinate with biologypress@plos.org. If you have previously opted in to the early version process, we ask that you notify us immediately of any press plans so that we may opt out on your behalf.

We also ask that you take this opportunity to read our Embargo Policy regarding the discussion, promotion and media coverage of work that is yet to be published by PLOS. As your manuscript is not yet published, it is bound by the conditions of our Embargo Policy. Please be aware that this policy is in place both to ensure that any press coverage of your article is fully substantiated and to provide a direct link between such coverage and the published work. For full details of our Embargo Policy, please visit http://www.plos.org/about/media-inquiries/embargo-policy/.

Thank you again for choosing PLOS Biology for publication and supporting Open Access publishing. We look forward to publishing your study.

Sincerely,

Christian

Christian Schnell, PhD

Senior Editor

PLOS Biology

cschnell@plos.org
